# Highly sensitive strain sensors based on piezotronic tunneling junction

**DOI:** 10.1038/s41467-022-28443-0

**Published:** 2022-02-09

**Authors:** Qiuhong Yu, Rui Ge, Juan Wen, Tao Du, Junyi Zhai, Shuhai Liu, Longfei Wang, Yong Qin

**Affiliations:** 1 School of Advanced Materials and Nanotechnology, Xidian University, 710071 Xi’an, Shaanxi China; 2 Institute of Nanoscience and Nanotechnology, School of Materials and Energy, Lanzhou University, 730000 Lanzhou, Gansu China; 3 Beijing Institute of Nanoenergy and Nanosystems, Chinese Academy of Sciences, 101400 Beijing, China; 4 College of Nanoscience and Technology, University of Chinese Academy of Sciences, 100049 Beijing, China

**Keywords:** Nanosensors, Sensors

## Abstract

Piezotronics with capacity of constructing adaptive and seamless interactions between electronics/machines and human/ambient are of value in Internet of Things, artificial intelligence and biomedical engineering. Here, we report a kind of highly sensitive strain sensor based on piezotronic tunneling junction (Ag/HfO_2_/n-ZnO), which utilizes the strain-induced piezoelectric potential to control the tunneling barrier height and width in parallel, and hence to synergistically modulate the electrical transport process. The piezotronic tunneling strain sensor has a high on/off ratio of 478.4 and high gauge factor of 4.8 × 10^5^ at the strain of 0.10%, which is more than 17.8 times larger than that of a conventional Schottky-barrier based strain sensor in control group as well as some existing ZnO nanowire or nanobelt based sensors. This work provides in-depth understanding for the basic mechanism of piezotronic modulation on tunneling junction, and realizes the highly sensitive strain sensor of piezotronic tunneling junction on device scale, which has great potential in advanced micro/nano-electromechanical devices and systems.

## Abstract (web summary)

Strain-induced piezoelectric polarization can be used to modulate the interface electrical transport. Here, the authors achieved a piezotronic tunneling strain sensor at device scale with optimized performance based on the structure of Ag/HfO2/n-ZnO.

## Introduction

Strain/pressure sensors that transduce a mechanical stimulus into an electrical signal have played a critical role and are of increasing demand in many existing and emerging applications, including Internet of Things, human–machine interfaces, wearable electronics, robotics, micro/nano-electromechanical systems (MEMS/NEMS), and biomedical engineering^1–7^. Up to now, based on different basic working mechanisms, the strain/pressure sensors are mainly divided into piezoresistive, capacitive, piezoelectric, and piezotronic types, etc. Among them, the ones based on piezoresistive, capacitive and piezoelectric effect have been widely used in industrial production for their excellent stability and mature circuit system^8^. Recently, with the rapid development of material science and nanotechnology, a large number of new principle-based strain/pressure sensors have emerged^9–11^. Piezotronic sensor is one of the emerging sensors that has attracted considerable interest particularly in interface engineering and is still focusing on fundamental science with strong expectancy of achieving high sensitivity^12,13^. Due to the coupling of piezoelectricity and charge carriers transport property of semiconductor, the piezotronic sensors utilize the strain-induced piezoelectric polarization charges and corresponding piezo-potentials at interfaces to linearly modulate the interface barrier height and exponentially control the carrier transport across the interface between metal-semiconductor, semiconductors, or semiconductor with other materials, thus to directly transduce a mechanical signal into an electrically control signal^14^. Because of the natural exponential relationship between input signal and output signal, strain sensor based on piezotronic effect has inherent advantages in detecting mechanical stimuli, and exhibits excellent performance in adaptive electronics and optoelectronics^14^, such as tactile imaging^2^, nano-LED pressure imaging^15^, high-electron-mobility transistor^16^, optofluidic logic computation^17^ and so on.

In recent years, the significant progress of piezotronic sensor has been made in both breadth and depth to enhance its performance^18^. On the one hand, the structure of piezotronic device has been expanded. The physical models of piezotronic sensor with different semiconductor structures (metal-semiconductor Schottky barrier^19,20^, p–n junction^21^, bicrystal interface^22^, two-dimensional electron gas^23^, etc.) have been explored. On the other hand, some effective strategies have been proposed to improve the device performance. The universal methods are: seeking piezoelectric semiconductor with high piezoelectric coefficients^24^ or special geometric characteristics^25^ to improve the efficiency of piezoelectric polarization charge generation; applying strategies such as introducing alloy structure^26^ and internal holes^27^ to weaken the electrostatic shielding effect of free carriers on the piezoelectric polarization charge; optimizing the device structure (e.g., double-channel structure^28^ and ion-gel-gating structure^29^) to improve the effectiveness and efficiency of the device’s use of the piezoelectric polarization charges. These studies mainly focused on the piezotronic modification of interface barrier height and improved the piezotronic effect on mechanical sensing. However, to further improve performance, not only material modification and device structure optimization are required, but also innovations in the piezotronic regulation mechanism are needed. In our previous work, by coupling piezotronic effect and quantum tunneling effect, a piezotronic tunneling junction (PTJ) with giant switching and fast response is verified in Pt/Al_2_O_3_/p-GaN with an atomic force microscopy (AFM)^30^. Unlike the working mechanism of only tuning the interface barrier height in traditional piezotronic devices, the high performance of PTJ results from simultaneous modulations of both the height and the width of tunneling barriers via the piezotronic effect. Previous research has reported a basic exploration of principle of piezotronic tunneling junction based on AFM, and whether it can be used for high-performance devices at macroscopic scale is a matter of great concern in piezotronics. Except packing, there are still many challenges need to be conquered for realizing a macroscopic piezotronic tunneling junction. For example, on the one hand, without AFM, the stress/strain can’t be precisely controlled, the piezotronic devices need to be able to withstand macroscopic dynamic stress/strain; on the other hand, the insulating layer of piezotronic tunneling junction is extremely thin, which is very easy to be damaged under uncontrollable strain during the fabrication of devices and can also easily cause the tunneling junction to be damaged under dynamic stress/strain. Therefore, the realization of high-performance piezotronic tunneling junction at the device scale is of great significance, which can provide scientific and technical support for the development of piezotronic devices with high performance.

In this article, a piezotronic tunneling strain sensor (PTSS) with optimized performance is achieved based on the structure of Ag/HfO_2_/n-ZnO. The characteristics of piezotronic modification on the interface tunneling transport is found to be divided into two stages: ultra-low strain (<0.01%) and relatively high strain (0.01–0.10%), corresponding to the dominant role of piezotronic regulation on barrier width and barrier height, respectively. Meanwhile, the experimental results show that the output signal of the PTSS increases by two orders of magnitude with applied strain ranged from 0.00 to 0.10%, which is 300.5 times that of the strain sensor based on Ag–ZnO Schottky barrier. The statistical results of 20 devices show that the PTSS can achieve a high on/off ratio of 478.4 and high gauge factor of 4.8 × 10^5^ at a strain of 0.10%, which shows potential in strain sensing. This work not only achieves the high performance of piezotronic tunneling junction at the device scale, but also provides in-depth understandings on the basic characteristics of the piezotronic modulation on the tunneling effect, which can expand the practical application of quantum tunneling effect in mechanical sensors.

## Results

### Working mechanism of PTSS

**Fig. 1:**
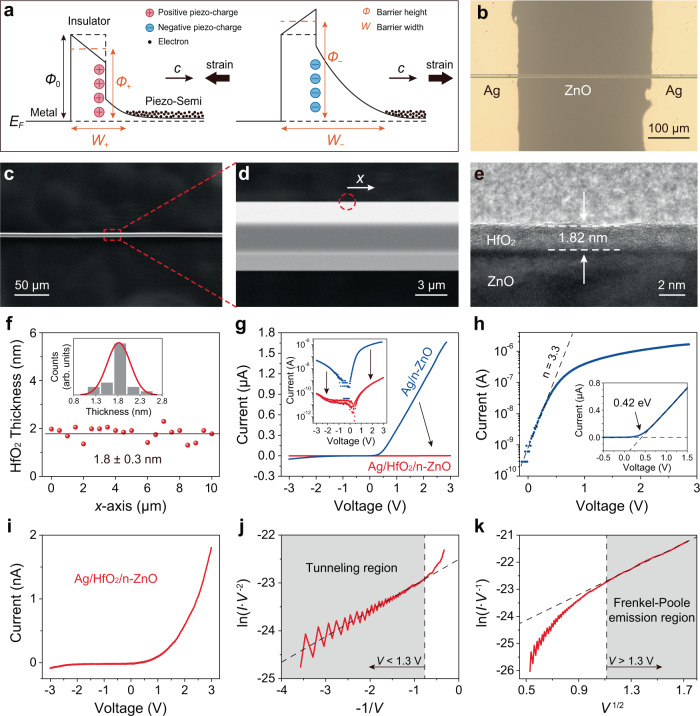
The working principle, structure and electrical transport of strain sensor based on piezotronic tunneling junction. **a** Schematics of the metal/insulator/piezoelectric semiconductor structure of piezotronic tunneling junction and corresponding conduction energy band profiles for the positive (left) and negative (right) piezoelectric polarizations at interfaces. A n-type semiconductor is taken as an example. Strain-induced positive piezoelectric polarization charges simultaneously decrease the tunneling barrier height (${\phi }_{+}$) and width (${W}_{+}$), which corresponds to the low resistance state of strain sensor; while, strain-induced negative piezoelectric polarization charges simultaneously increase the tunneling barrier height (${\phi }_{-}$) and width (${W}_{-}$), which corresponds to the high resistance state of strain sensor. The energy band diagrams with and without piezoelectric charges are shown by the black solid and dashed lines, respectively. **b** Optical image of the piezotronic tunneling strain sensor, an individual n-ZnO nanowire coated with atomic thickness of HfO_2_ insulator, clamping at its two ends by Ag electrodes. **c**, **d** SEM images of the ZnO nanowire with a diameter of about 8 μm, showing a typical hexagonal geometry and a clean surface. **e**, **f** High-resolution TEM image of HfO_2_ deposited on ZnO surface and the distribution of HfO_2_ thickness along the *x*-axis in **d**, showing the HfO_2_ thickness of about 1.8 (±0.3) nm. The inset shows the statistical distribution of the insulating thickness. **g**–**k** Carrier transports of the Ag/HfO_2_/n-ZnO piezotronic tunneling junction (red curves) and the Ag/n-ZnO Schottky junction (blue curves) without strain. The semilogarithmic *I*–*V* curve of Ag/n-ZnO (**h**) shows a Schottky barrier height of about 0.42 eV and an ideal factor of about 3.3. The *I*–*V* (**i**), ln(*I* ∙ *V*^−2^) versus -1/*V* (**j**), and ln(*I* ∙ *V*^−1^) versus *V*^1/2^ (**k**) curves of Ag/HfO_2_/n-ZnO indicate that tunneling and Frankel-Poole emission correspond to carrier transport mechanisms at low and high bias voltages, respectively.

The working mechanism of PTSS employing a metal/insulator/piezoelectric semiconductor structure is depicted schematically in Fig. 1a, using an n-type semiconductor as an example. If the piezoelectric polar *c*-axis points right away from the insulator, a compressive strain will induce positive piezoelectric charges at the insulator/semiconductor interface (Fig. 1a, left), which lowers the barrier height (*ϕ*_+_) on the one hand, and reduces the barrier width (*W*_+_) by driving the n-type semiconductor surface into less depletion of majority carriers on the other hand. According to the theory of quantum mechanics, the tunneling transmittance depends exponentially on the barrier width as well as on the barrier height. An enhanced strain-controlled carriers’ transport of PTSS can be expected as the barrier width is significantly tuned in parallel with the barrier height. Thus, the PTSS is in a low resistance state. As the strain is reversed, as shown in Fig. 1a (right), negative polarization charges generated at interface will increase the barrier height (*ϕ*_−_) and barrier width (*W*_−_) simultaneously, so that electrons need more energy to move through the junction. Moreover, the tunneling electrons have to experience an extra barrier in the depleted space charge region owing to the band bending induced by piezoelectric polarization in the barrier. The tunneling transmittance can be seriously decreased by this extra barrier. Thus, the PTSS changes into a high resistance state.

### Device fabrication and structure of PTSS

The detailed fabrication processes are described in the Methods. The PTSS with a structure of Ag/HfO_2_/n-ZnO consists of an individual n-ZnO nanowire coated with atomic thickness of HfO_2_ insulator and two Ag electrodes clamped at the nanowire’s ends (Fig. 1b). The ZnO nanowires were grown by chemical vapor deposition and transferred to a flexible substrate PET. Scanning electron microscopy (SEM) images, transmission electron microscopy (TEM) images and X-ray diffraction spectra show that the uniform ZnO nanowire with a typical hexagonal geometry has a clean surface and a good wurtzite crystallinity (Fig. 1c, d and Supplementary Note 1 and Supplementary Figs. 1 and 2). Its piezoelectric property and polarization *c*-axis can be obtained and determined through the piezoresponse force microscopy (PFM) and the nanogenerator measurement, respectively (Supplementary Note 2 and Supplementary Figs. 3 and 4). The insulating layer HfO_2_ was precisely grown on the ZnO nanowire by atomic layer deposition (ALD). High-resolution transmission electron microscopy (HTEM) image of HfO_2_ (Fig. 1e) deposited on ZnO surface and the statistical distribution of HfO_2_ thickness (Fig. 1f) along the *x*-axis labeled in Fig. 1d, indicate the HfO_2_ is uniform with a thickness of about 1.8 (±0.3) nm.

### Electrical transport of PTSS

Prior to the piezotronic modification of *I*–*V* measurements, we firstly studied the electrical transport of Ag/HfO_2_/n-ZnO PTSS and Ag/n-ZnO Schottky-junction-based strain sensor (SSS) without strain. As shown in Fig. 1g, the insertion of the HfO_2_ insulating layer between Ag and ZnO changes the energy band barrier of the original Ag-ZnO contact, resulting in a significantly different *I*–*V* curve of PTSS (red curves) from that of SSS (blue curves). Taking the logarithm of the current of Ag/n-ZnO device (Fig. 1h), the slope of the obtained curve under a small bias (ideal factor) is about 3.3, demonstrating the existence of surface states at the Schottky barrier^31^. Also, we can simply estimate the Schottky barrier height of approximately 0.42 eV from the inset of Fig. 1h. Furthermore, we analyzed the *I*–*V* characteristics (Fig. 1i) of Ag/HfO_2_/n-ZnO PTSS in detail. By plotting the ln(*I*∙*V*^−2^) versus −1/*V* (Fig. 1j) curve and ln(*I*∙*V*^−1^) versus *V*^1/2^ (Fig. 1k) curve, we found that the carrier transport mechanism of PTSS at low and high bias voltages, respectively corresponds to tunneling and Frankel-Poole emission^31^. This in turn confirms the successful construction of the Ag/HfO_2_/n-ZnO tunneling junction.

### Strain-dependent *I*–*V* characteristics of PTSS and SSS

**Fig. 2:**
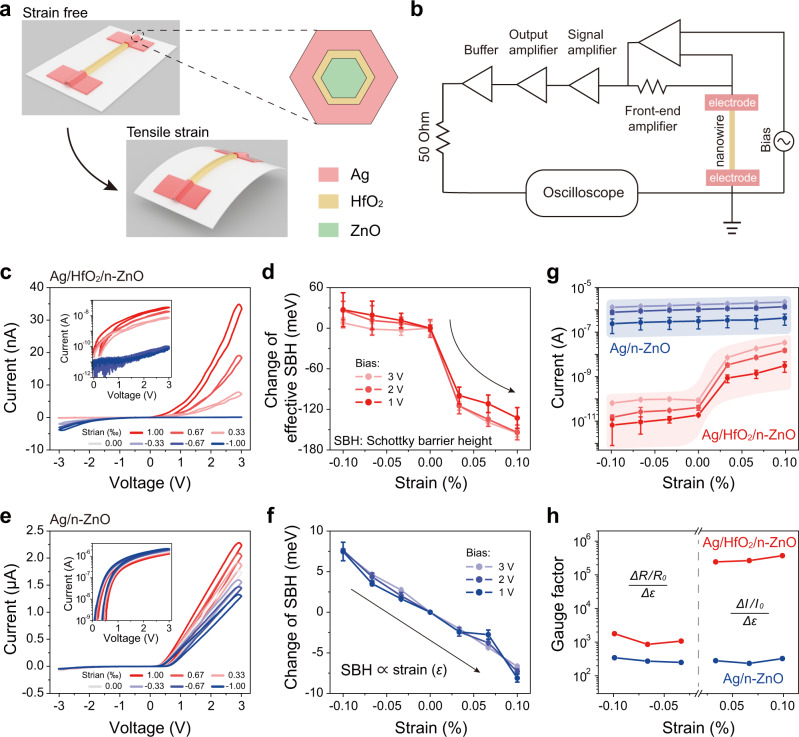
The interface gating effect of strain sensor based on piezotronic tunneling junction. **a** Cyclic bending of the flexible substrate introduces a strain to the piezotronic tunneling strain sensor (PTSS). The strain of the nanowire can be approximately equivalent to the strain of the outer surface of the flexible substrate. **b** Schematic diagram of the measurement setup. Using this equipment, a sweeping bias between −3 and +3 V was applied and the current was measured simultaneously. **c** Strain-dependent *I*–*V* characteristics of Ag/HfO_2_/n-ZnO PTSS. The inset is the semilogarithmic forward curves. As the tensile strain increases, the current increases drastically. **d** Change of effective Schottky barrier height (SBH) derived from the *I*–*V* curves in **c**. The rapidly changed SBH between the strain of 0.00–0.033% indicates that a small tensile strain enables the occurrence of carrier tunneling. **e** Strain-dependent *I*–*V* characteristics of Ag/n-ZnO Schottky-junction-based strain sensor (SSS). The inset is the semilogarithmic forward *I*–*V* curves. **f** Change of effective SBH derived from the *I*–*V* curves in **e**. A linear correlation between the change of SBH with applied strain follows the classic piezotronic theory. **g** ln(*I*) versus strain curves of Ag/HfO_2_/n-ZnO device (red) and Ag/n-ZnO device (blue). **h** Gauge factor derived from **g** as a function of strain to assess the performance of PTSS and SSS. The error bars in **d**, **f**, and **g** are the standard derivations.

As shown in Fig. 2a, the Ag/HfO_2_/n-ZnO PTSS can be periodically strained in tension or compression. With consideration of the extremely small diameter of the ZnO nanowire in comparison with the thickness of PET substrate, the axial strain along the length of the ZnO nanowire can be precisely calculated from the bending degree of the substrate (Supplementary Note 3 and Supplementary Fig. 5). Figure 2b shows an electrical measurement system that is used to apply a sweeping voltage to the sensor and simultaneously measure the electrical characteristics. We measured the strain-dependent *I*–*V* curves of Ag/HfO_2_/n-ZnO PTSS in Fig. 2c. When no strain is applied, the forward current is smaller than the reverse current, indicating that the tunneling junctions at two terminals of the device are different (Supplementary Note 4 and Supplementary Fig. 6). As the applied strain changed from compressive strain to tensile strain, the forward current increases, while the reverse current decreases. This strain-controlled asymmetric modulation indicates that the dominant mechanism of PTSS is the piezotronic effect, rather than some other effects, such as piezoresistive effect^2,14^ or influence of interface traps with strain (detailed in Supplementary Note 5 and 6 and Supplementary Figs. 7–11). It is worth noting that the forward current monotonically increases by two orders of magnitude with increasing the tensile strain from 0.00 to 0.10%, which is 300.5 times that of SSS, preliminary indicating that PTSS has a much larger on/off current ratio compared with SSS. To further analyze the piezotronic modification of carrier tunneling transport, we calculated the change of effective Schottky barrier height (SBH) as a function of applied strain (Fig. 2d). A rapid change of effective SBH can be found with the strain increases from 0.00 to 0.033%, indicating that a small tensile strain enables a greatly increased tunneling transmittance and the occurrence of carrier tunneling. If the tensile strain is further increased from 0.033 to 0.10%, although the effective SBH will continue to decrease, the magnitude of its change will be slower than that of range from 0.00 to 0.033%, which further shows that the rapid increase of tunneling current mainly occurs under a small strain.

As a contrast, we also performed the *I*–*V* characteristics of Ag/n-ZnO SSS in Fig. 2e, in which the current changes slowly and slightly in comparison of the Ag/HfO_2_/n-ZnO PTSS. In Fig. 2f, by calculating the change of SBH, which is lower than the actual value due to the side contact between ZnO nanowire and the electrode (Supplementary Note 7 and Supplementary Fig. 12), a linear relationship between the change of SBH and the applied strain is found to follow the piezotronic theory in Schottky barrier case (Supplementary Note 8 and 9 and Supplementary Figs. 13 and 14). These results further confirm the mechanism of the piezotronic tunneling junction in PTSS.

The plots of current versus strain in Fig. 2g are also extracted from Fig. 2c, e, showing the consistency of the variation trends of the change of SBH and the change of current with applied strain. In order to preliminarily estimate the performance of PTSS, we derived the gauge factor, defined as the ratio of relative change in current or resistance to the mechanical strain (Supplementary Note 10), of PTSS and SSS in Fig. 2h. The highest gauge factor of Ag/HfO_2_/n-ZnO PTSS reaches 3.8 × 10^5^, which is much higher than that of Ag/n-ZnO SSS (2.9 × 10^2^, consistent to the magnitude reported in ref. ^32^). It shows the advantages of piezotronic tunneling junction in strain sensor in comparison of metal-semiconductor Schottky junction.

### Piezotronic modification on tunneling barrier height and width

**Fig. 3:**
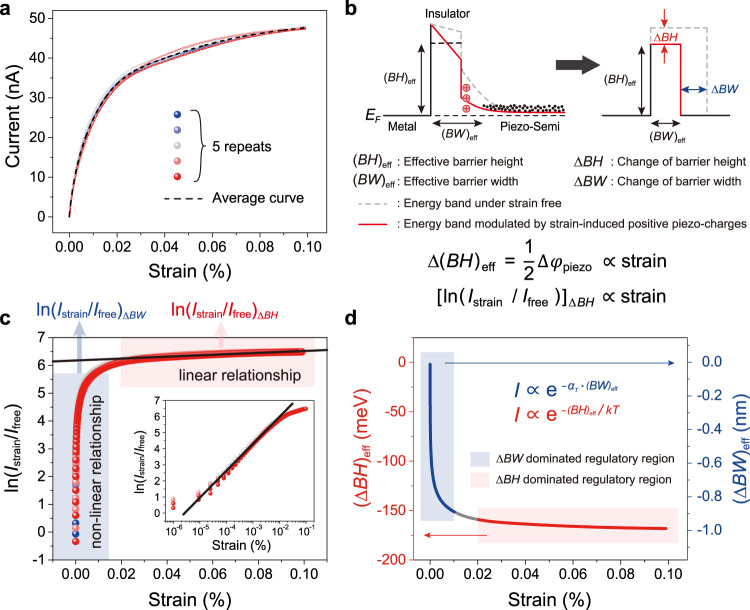
Piezotronic modification on the tunneling barrier height and width. **a** Five cycles of current response to continuously changed tensile strain. The currents first increase rapidly and then slowly with increasing strain. **b** Energy band profile of tunneling junction. The metal/insulator/piezoelectric semiconductor tunneling barrier equivalent to a rectangular barrier, whose effective height and width are (BH)_eff_ and (BW)_eff_, respectively, and their corresponding strain-induced changes are denoted as (ΔBH)_eff_ and (ΔBW)_eff_. The part in ln(*I*_strain_/*I*_free_) that has a linear relationship with strain is induced by the change of barrier height (ΔBH)_eff_. **c** ln(*I*_strain_/*I*_free_) as a function of strain. The regions that change drastically (blue region, strain <0.01%) and slowly (red region, strain >0.02%) with strain correspond to different dominant mechanisms: the former is dominated by (ΔBW)_eff_, and the latter is dominated by (ΔBH)_eff_, both of which caused by the piezotronic effect. **d** Change of the effective barrier height and width derived from **c** as a function of applied strain.

To further investigate the piezotronic effect on the barrier height and width of the tunneling junction of PTSS, we measured five cycles of dynamic currents response to continuously changed tensile strain, as shown in Fig. 3a. We can find the currents (including the average current) first increase rapidly and then slowly with increasing strain. Considering that both the height and width of a barrier have a natural exponential relationship with the current, as described in the following formulas^31^,

1 $$I\propto {{{{{{\rm{e}}}}}}}^{-\frac{{\left({{{{{{\mathrm{BH}}}}}}}\right)}_{{{{{{\rm{eff}}}}}}}}{{kT}}},$$

2 $$I\propto {{{{{{\rm{e}}}}}}}^{-{\alpha }_{T}{({{{{{{\mathrm{BW}}}}}}})}_{{{{{{\rm{eff}}}}}}}},$$

where ${\alpha }_{{{{{{\rm{T}}}}}}}$ is a constant with unit of m^−1^, *k* is Boltzmann constant, and *T* is the absolute temperature. Here, we simply equate the metal/insulator/piezoelectric semiconductor tunneling barrier to a rectangular barrier (Fig. 3b), whose effective height and width are (BH)_eff_ and (BW)_eff_, respectively, and their corresponding strain-induced changes are denoted as (ΔBH)_eff_ and (ΔBW)_eff_. Based on piezotronic theory, the (ΔBH)_eff_ can be expressed as

3 $$\triangle {\left({{{{{{\mathrm{BH}}}}}}}\right)}_{{{{{{\rm{eff}}}}}}}=\frac{1}{2}\triangle {\varphi }_{{{{{{\rm{piezo}}}}}}}\propto {{{{{\rm{strain}}}}}},$$

and the current influenced by the change of barrier height satisfies

4 $${[{{{{\mathrm{ln}}}}}({I}_{{{{{{\rm{strain}}}}}}}/{I}_{{{{{{\rm{free}}}}}}})]}_{\varDelta BH}\propto {{{{{\rm{strain}}}}}}$$

Thus, the part (ln(*I*_strain_/*I*_free_)_*ΔBH*_) in ln(*I*_strain_/*I*_free_) that has a linear relationship with strain is induced by the change of barrier height (ΔBH)_eff_. By plotting the ln(*I*_strain_/*I*_free_) as a function with the strain (Fig. 3c), we can find the value of ln(*I*_strain_/*I*_free_) changes approximately linearly with strain in the red region (strain >0.02%), which is corresponding to the change of barrier height regulated by piezotronic effect; whereas changes drastically and nonlinearly with strain in the blue region (strain <0.01%), resulting from the simultaneous piezotronic modification on the tunneling barrier height and width (Supplementary Note 11–13 and Supplementary Figs. 15–19). The enlarged detail of the blue region is shown in the inset of Fig. 3c, showing an approximate linear relationship between the ln(*I*_strain_/*I*_free_) and the exponential form of strain. This relationship differs from the case in Schottky device (including SSS), indicating a complex piezotronic modulation on the piezotronic tunneling junction in PTSS, which requires further experimental and theoretical research.

Using the relation (1) and (2), we can approximate the change in the equivalent barrier height or barrier width as plotted in Fig. 3d. We can find that the (ΔBH)_eff_ is approximately proportional to the strain, in which a strain of 0.07% (from 0.03 to 0.10%) will cause an effective barrier height change of about 6.31 meV. This regulation magnitude is roughly consistent with that of SSS in Fig. 2f. Therefore, it is reasonable to believe that the piezotronic modulation on the piezotronic tunneling junction in PTSS will become similar to that on the Schottky barrier in SSS when the strain is large enough. The main reason is that when the strain is large enough, the PTSS is already turned to a high tunneling current “ON” state, and the strain-induced changes of barrier width is almost negligible (Supplementary Note 12). Whereas, when the strain is small (<0.01%) corresponding to the blue region in Fig. 3d, the rapidly changed current results from the simultaneous piezotronic modification on the tunneling barrier height and width, and the latter plays a dominant role. This result is consistent with the strain-dependent *I*–*V* measurements of PTSS in Fig. 2c. One more thing to note is that the consistency of the five cycles’ measurements of response current in Fig. 3a shows that the piezotronic tunneling junction is not damaged within the strain range of 0.00–0.10% in five cycles.

### Dynamic current of PTSS and SSS response to applied strain

**Fig. 4:**
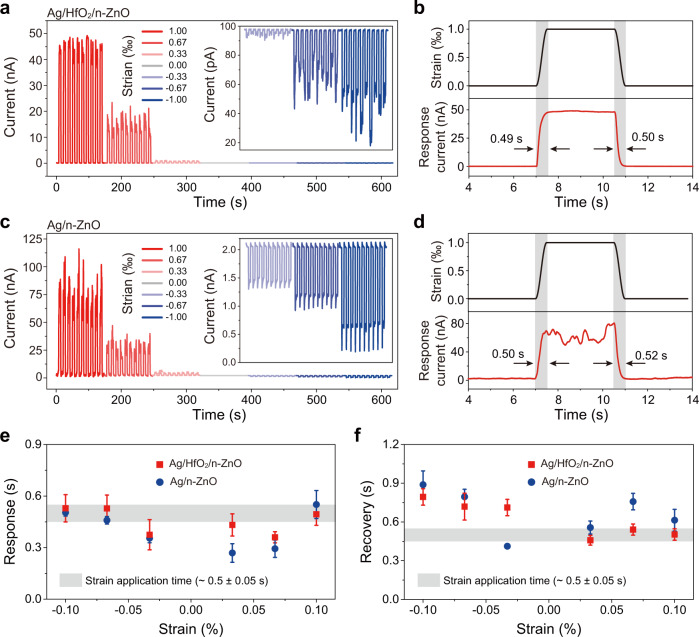
Dynamic current of the Ag/HfO_2_/n-ZnO PTSS and the Ag/n-ZnO SSS response to applied strain. **a** Current response of the Ag/HfO_2_/n-ZnO PTSS to strain from −0.10 to +0.10% at a fixed bias. The inset is the enlarged image of the current response to compressive strain. **b** Detailed response current under a tensile strain of 0.10% for Ag/HfO_2_/n-ZnO PTSS. The corresponding response time and recovery time are 0.49 and 0.50 s, respectively. **c** Current response of the Ag/n-ZnO SSS to strain from −0.10 to +0.10% at a fixed bias of 3 V. The inset is the enlarged image of the current response to compressive strain. **d** Detailed response current under a tensile strain of 0.10% for Ag/n-ZnO SSS. The corresponding response time and recovery time are 0.50 s and 0.52 s respectively. **e**, **f** Statistical response time (**e**) and recovery time (**f**) of the Ag/HfO_2_/n-ZnO PTSS and Ag/n-ZnO SSS strain sensor under different strains, respectively. Time to apply strain is about 0.5 (±0.05) s. The error bars in **e**, **f** denote standard deviations of the mean.

Next, we measured the dynamic current response of the Ag/HfO_2_/n-ZnO PTSS as shown in Fig. 4a. In the experiment, a periodic mechanical strain was performed on the device through a high-precision actuator, and at the same time the dynamic current response to strain was monitored by a synchronous electrical measurement system. The current baseline corresponding to strain free does not fluctuate seriously throughout the test; while the current value changes significantly upon applying strain, particularly the tensile strain, demonstrating the strain sensing performance of PTSS. As can be seen, under actions of ten on/off cycles with different magnitudes of both compressive and tensile strains, the current is highly reversible and exhibits good stability of PTSS. By enlarging the detail of the current response of PTSS (Fig. 4b and Supplementary Fig. 20), we can respectively estimate its response time (rise time) and recovery time (fall time) of about 0.49 and 0.50 s under a tensile strain of 0.10%.

As a comparison, we also performed a same characterization on the Ag/n-ZnO SSS (Fig. 4c). As excepted, the amplitude of response current is larger than that of PTSS, and the response time (Fig. 4d and Supplementary Fig. 21) are similar to that of PTSS. Additionally, by calculating response-recovery time curves of twenty cycles under different strains (Supplementary Figs. 22–25), we found that the response time (Fig. 4e) and the recovery time (Fig. 4f) of PTSS and SSS under the same strain are almost equal, and the distribution of response and recovery time of the two sensors are 0.25–0.65 s and 0.33–0.98 s, respectively.

### Strain-sensing performance of PTSS and SSS

**Fig. 5:**
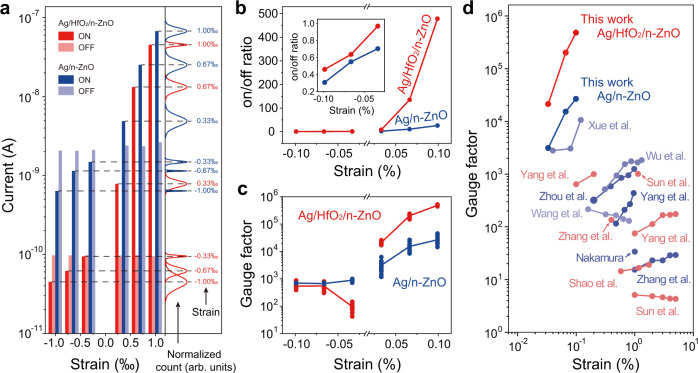
Strain-sensing performance of the Ag/HfO_2_/n-ZnO PTSS and the Ag/n-ZnO SSS. **a** Statistical distributions and histograms of the “ON” state and “OFF” state current of the Ag/HfO_2_/n-ZnO PTSS and Ag/n-ZnO SSS upon compressive and tensile strains. **b**, **c** On/off ratio (**b**) and gauge factor (**c**) of Ag/HfO_2_/n-ZnO PTSS and Ag/n-ZnO SSS as a function of applied strain derived from **a**. **d** Comparison of the gauge factor of this work (including Ag/HfO_2_/n-ZnO PTSS and Ag/n-ZnO SSS) with some existing ZnO nanowire or nanobelt based sensors.

In order to further study the strain-sensing performance of PTSS, we summarized and compared the device performance of PTSS and SSS as well as some existing ZnO nanowires or nanobelts based sensors in Fig. 5. Through statistical analysis of the dynamic current under different strains (Supplementary Figs. 26 and 27), we plotted the statistical distributions and histograms of the current (“ON” state and “OFF” state) of the Ag/HfO_2_/n-ZnO PTSS and Ag/n-ZnO SSS upon compressive and tensile strains in Fig. 5a. The statistical current increases with the increase of tensile strain, and decreases with the increase of compressive strain. This trend is consistent to the *I*–*V* characteristics in Fig. 2c, e. It can also be seen that although the “OFF” current of PTSS is much smaller than that of SSS, the “ON” current of PTSS is almost of the same order as that of SSS under tensile strain. The on/off ratio has been calculated and shown in Fig. 5b, owing to the carrier tunneling in PTSS under a tensile strain, the “ON” state current of PTSS rapidly increases with increasing strain, resulting in a large on/off ratio of 478.4, which is 18.6 times that of SSS. We also measured PTSS with different thicknesses (0.4, 1.1, 1.8, 2.5, 3.6, and 7.3 nm) of HfO_2_ and found that the devices with 1.8 nm HfO_2_ exhibit the highest performance (Supplementary Note 14 and Supplementary Figs. 28–30).

We also calculate the gauge factor of PTSS and SSS in Fig. 5c. Under tensile strain, the curves of gauge factor of PTSS and SSS have the same changing trend, both of which increase approximately exponentially with the increase of strain. Whereas, the statistical results of twenty devices show that the average gauge factor of PTSS is 4.8 × 10^5^ at a tensile strain of 0.10%, which is much larger than that (~2.7 × 10^4^) of SSS. Figure 5d summarizes the gauge factor of PTSS, SSS and some existing ZnO nanowire or nanobelt based sensors^29,32–43^. The PTSS based on the Ag/HfO_2_/n-ZnO shows the obvious advantages than other sensors (including SSS), demonstrating its great potential in strain sensing. The high sensitivity of PTSS can be attributed to the mechanism of piezotronic regulation on the tunneling barrier height and width in parallel (Fig. 3), and thus tuning the tunneling current under small tensile strain.

## Discussion

In summary, a kind of strain sensor based on piezotronic tunneling junction with structure of Ag/HfO_2_/n-ZnO at device scale is developed. The modulating mechanism of piezotronic effect on the tunneling transport is investigated under small strain (<0.01%) and relatively large strain (0.01–0.10%), respectively. Due to the synergistical modulation effect of piezoelectric polarization charge on both the tunneling barrier height and width, piezotronic tunneling strain sensor exhibits high sensitivity with on/off ratio of 478.4 and gauge factor of 4.8 × 10^5^ at a tensile strain of 0.10%, which exhibits greater advantages in comparison with conventional Schottky-barrier based strain sensors and some other existing ZnO nanowire or nanobelt based sensors. This work promotes the in-depth combination of piezotronic effect and tunneling effect, benefiting to the development of high-performance piezotronic devices and strain sensors.

## Methods

### Synthesis of ZnO nano/microwires

ZnO nano/microwires used in the experiment were synthesized using a chemical vapor deposition (CVD) method through a vapor-solid process^44^. First, 2.0 g of ZnO powder and 0.4 g of graphite powder were ground in an agate mortar to obtain a uniform mixture. The obtained mixture was put into an alumina boat and placed at the center of a furnace tube. Then, 240 standard-state cubic centimeters per minute (sccm) inert argon and 12 sccm oxygen were introduced into the furnace tube. Afterwards, the furnace was heated to 1150 °C for 40 min and then to 1250 °C for 40 min. ZnO nano/microwires grown near the outlet of the furnace tube were obtained.

### Fabrication of nanometer-thick HfO_2_ insulator barrier

Ultrathin HfO_2_ layer were grown over ZnO nano/microwire surface in a continuous-flow ALD reactor operated under a base pressure. First, we selected a certain number of single ZnO nano/microwires to put on a glass substrate that has been ultrasonically cleaned with acetone and ethanol for multiple times, and then loaded them into the ALD reaction chamber. Here, the grown process of HfO_2_ layer on the surface of ZnO nano/microwires was in Savannah G2 S200 PEALD system with tetrakis(ethylmethylamino)hafnium (TDMAHf, Hf[N(CH_3_)_2_]_4_) and H_2_O as precursors. The key process parameters were as follows: pressure ~0.34 Torr, temperature ~150 °C, 20 sccm N_2_ carrier gas, 0.015 s cycle-1 H_2_O, 0.1 s cycle-1 TDMAHf, 10 s purge time. The thickness of the final interfacial HfO_2_ layer deposited on ZnO nano/microwires is about 1.8 nm. By changing the number of depositions, HfO_2_ of different thicknesses can be obtained.

### Fabrication of strain sensor with piezotronic tunneling junction

The tailored polyethylene terephthalate (PET) films (typically 3.00 cm × 0.50 cm × 0.15 mm) were used as the supporting substrate of the strain sensor, which were cleaned by acetone, alcohol and deionized water. First, transferred the prepared HfO_2_/n-ZnO nano/microwires in the middle of the PET flexible substrate. Then, we made Ag electrodes on both ends of the nano/microwires by thermal evaporation using RZF-300 thermal evaporation coating system, and fixed them with silver paste, and the copper wires were bonded to the silver electrodes to lead the source and drain electrode. Finally, a thin layer of polydimethylsiloxane (PDMS) was applied to encapsulate the entire device for dielectric protection and prevent the damage of the sensor under repeated manipulation. The ZnO nano/microwire strain sensors (SSS) were fabricated by the same method. After treating the ZnO nano/microwires, they were directly transferred to the PET films, and the devices were packaged after the Ag electrodes were made.

### Material characterization

The morphology of nanowires and the thickness of the HfO_2_ layer deposited on ZnO were characterized utilizing a TEM (JEM-2100F) and a SEM (SU8020, Hitachi). The X-ray diffraction spectra of materials were characterized by Bruker D8 ADVANCE. The piezoelectricity of ZnO nano/microwire was investigated by AFM (Cypher ES, Asylum Research) with PFM (piezoresponse force microscopy) mode.

### Electrical measurements of strain sensor

Low noise preamplifiers (SR570, SR560) were used to measure the output voltage and current, and PCI-6259 (National Instruments) was used for data collection. In the measurements of *I*–*V* characteristics, a sweeping voltage between −3 V and +3 V was applied across the device. And in the periodic *I*-*t* characteristic test, the voltage applied was +3 V. The specific strain of devices was driven by a DC linear motor.

### Reporting summary

Further information on research design is available in the Nature Research Reporting Summary linked to this article.

## Supplementary information

Supplementary Information

Inventory of Supporting Information

Reporting Summary

## Data Availability

The data that support the findings of this study are available from the corresponding author upon reasonable request.
