# Supplementary material for: Highly sensitive strain sensors based on piezotronic tunneling junction
Source: Nat Commun. 2022 Feb 9;13:778. doi: 10.1038/s41467-022-28443-0 (PMC8828782; doi:10.1038/s41467-022-28443-0)
Supplement: Supplementary file 1 — Supplementary Information [file 41467_2022_28443_MOESM1_ESM.pdf]

Supplementary Information for
Highly Sensitive Strain Sensors Based on Piezotronic Tunneling Junction

Qiuhong Yu[1,2†], Rui Ge[1†], Juan Wen[2], Tao Du[1], Junyi Zhai[3,4], Shuhai Liu[2], Longfei Wang[3,4]* and Yong Qin[2]**

[1]School of Advanced Materials and Nanotechnology, Xidian University, Xi'an, Shaanxi 710071, China
[2]Institute of Nanoscience and Nanotechnology, School of Materials and Energy, Lanzhou University, Lanzhou, Gansu 730000, China
[3]Beijing Institute of Nanoenergy and Nanosystems, Chinese Academy of Sciences, Beijing 101400, China
[4]College of Nanoscience and Technology, University of Chinese Academy of Sciences, Beijing 100049, China

†These authors contributed equally to this work.
**Corresponding author E-mail: liushuhai1991@live.cn, lfwang12@binn.cas.cn, qinyong@lzu.edu.cn*

This PDF file includes:

Supplementary Notes 1-14:

Supplementary Note 1 | Material Characterization
Supplementary Note 2 | Piezoelectricity in wurtzite ZnO
Supplementary Note 3 | Calculation of the Strain of the Sensors
Supplementary Note 4 | Asymmetry *I-V* Characteristics of the Tunneling Junctions without Strain
Supplementary Note 5 | Interface Traps/Defects in Tunneling Junctions
Supplementary Note 6 | Different Regulation Mechanisms of Piezoelectric Charges and Interface Traps/Defects on Electrical Transport under Strain
Supplementary Note 7 | Influence of the Ag/n-ZnO Contact Area of the Side Surface on the Performance of the Strain Sensor
Supplementary Note 8 | Electrical Transport of the Ag/n-ZnO Schottky-Junction -Based Strain Sensor (SSS)

Supplementary Note 9 | Calculation of the Change of Schottky Barrier Height and Its Linear Relationship with Strain
Supplementary Note 10 | Calculation of the Gauge Factor and the Current On-Off Ratio
Supplementary Note 11 | Theory of Piezotronic Effect on the Metal-Insulator -Semiconductor Junction

Supplementary Figures 1-31:

Supplementary Fig. 1 | Scanning electron microscopy (SEM) and transmission electron microscopy (TEM) images of ZnO microwire.
Supplementary Fig. 2 | The XRD spectrum of ZnO nanowires.
Supplementary Fig. 3 | Piezoelectricity in wurtzite ZnO.
Supplementary Fig. 4 | Piezoelectric nanogenerators under tensile strain and compressive strain to determine the polarity of ZnO microwire.
Supplementary Fig. 5 | Schematic of the measurement system.
Supplementary Fig. 6 | *I-V* characteristics of Ag/HfO_2/n-ZnO devices.
Supplementary Fig. 7 | Influence of interface traps on *C-V* curves of Ag/HfO_2/n-ZnO tunneling junctions.
Supplementary Fig. 8 | Strain-induced symmetric modulation of electrical transport by interface traps/defects.
Supplementary Fig. 9 | Strain-induced asymmetric modulation of electrical transport by piezotronic effect.
Supplementary Fig. 10 | Experiment results of *I-V* characteristics modulated by strain.
Supplementary Fig. 11 | Comparison of the strain-tuned *I-V* characteristics of two terminal devices with contact of Ag/HfO_2/n-ZnO and Ag/HfO_2/n-Si.
Supplementary Fig. 12 | The Ag/n-ZnO contacts at end/side surfaces and the equivalent circuits.
Supplementary Fig. 13 | Schematic of a metal-semiconductor-metal (MSM) piezotronic transistor and equivalent circuit of MSM piezotronic transistor.
Supplementary Fig. 14 | Ideal metal-semiconductor Schottky contacts with the presence of piezoelectric charges at an applied voltage V=0 (thermal equilibrium).
Supplementary Fig. 15 | Ideal metal-insulator-piezoelectric semiconductor contact with the presence of piezoelectric charges when applying positive voltage to metal.
Supplementary Fig. 16 | Barrier profiles.

Supplementary Fig. 17 | Potential distribution of the tunneling junction.
Supplementary Fig. 18 | Piezotronic modification of *C-V* characteristics of MIS tunneling junction.
Supplementary Fig. 19 | $\ln(I_{\text{strain}}/I_{\text{free}})$ as a function of strain.
Supplementary Fig. 20 | Current response-recovery time curve for Ag/HfO_2/n-ZnO PTSS.
Supplementary Fig. 21 | Current response-recovery time curve for Ag/n-ZnO SSS.
Supplementary Fig. 22 | The statistical distribution of the response time for Ag/HfO_2/n-ZnO PTSS.
Supplementary Fig. 23 | The statistical distribution of the recovery time for Ag/HfO_2/n-ZnO PTSS.
Supplementary Fig. 24 | The statistical distribution of the response time for Ag/n-ZnO SSS.
Supplementary Fig. 25 | The statistical distribution of the recovery time for Ag/HfO_2/n-ZnO PTSS.
Supplementary Fig. 26 | The statistical distribution of the on-state and off-state current for Ag/HfO_2/n-ZnO PTSS.
Supplementary Fig. 27 | The statistical distribution of the on-state and off-state current for Ag/n-ZnO SSS.
Supplementary Fig. 28 | *C-V* characteristics of MIS tunneling junctions with various insulator thicknesses.
Supplementary Fig. 29 | Measured *C-V* characteristics of Ag/HfO_2/n-ZnO tunneling junctions for various insulator thicknesses.
Supplementary Fig. 30 | The on/off ratio of Ag/HfO_2/n-ZnO tunneling junctions with various insulator thicknesses.
Supplementary Fig. 31 | The statistical distribution of the response time of this work (including Ag/HfO_2/n-ZnO PTSS and Ag/n-ZnO SSS) and some other sensors.

Supplementary Tables 1-2:

Supplementary Table 1 | Response Time for Some Strain Sensing Works.
Supplementary Table 2 | Comparison of Strain Sensing Works.

References 1-85.

Supplementary Note 1 | Material Characterization

The ZnO nano/microwires are synthesized simultaneously using a chemical vapor deposition (CVD) method through a vapor-solid process[1]. To avoid the pollution, the ZnO nano/microwires are then store in the inert environment, and are used to make devices within a few days. Supplementary Fig. 1 exhibits the scanning electron microscopy (SEM) image and the transmission electron microscopy (TEM) images of ZnO nano/microwire, which show its surfaces in detail. The clear lattice profiles indicate that the synthesized ZnO nano/microwires used for Metal-Insulator-Semiconductor (MIS) tunneling junctions have a good crystallinity and flat surface. In addition, the X-ray diffraction spectra in Supplementary Fig. 2 also demonstrates the highly crystalline nature of ZnO nano/microwires.

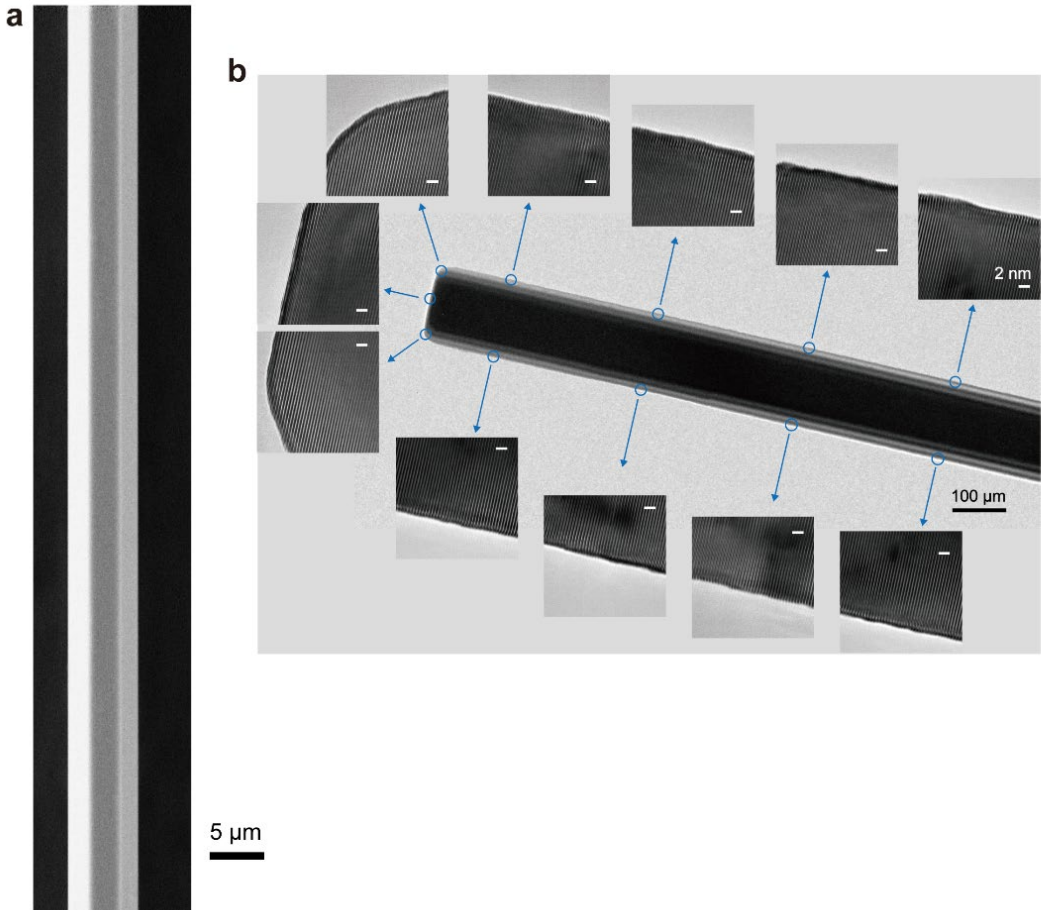

Supplementary Fig. 1 | Scanning electron microscopy (SEM) and transmission electron microscopy (TEM) images of ZnO microwire. a, SEM and **b,** TEM images of ZnO microwire used in this work. The 11 insets in (**b**) exhibit the surface quality of the ZnO nano/microwire at different locations labeled in blue circles. The scale bars of insets represent 2 nm.

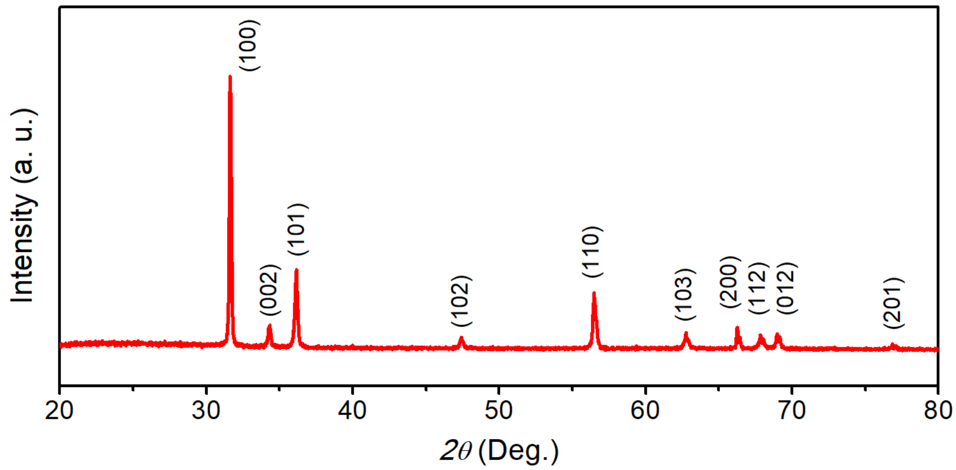

Supplementary Fig. 2 | The XRD spectrum of ZnO nanowires. The corresponding peaks of (100), (002), (101), (102), (110), (112) and (201) planes indicate that the ZnO nano/microwires were highly crystalline in nature.

Supplementary Note 2 | Piezoelectricity in wurtzite ZnO

Piezoelectric semiconductor ZnO has the wurtzite structure, which has a hexagonal unit cell with space group C6mc. The ±(0001) facets of ZnO are terminated with zinc and oxygen, and hence the entire structure lacks of central symmetry. The structure of ZnO can be simply described as numbers of alternating planes composed of tetrahedrally coordinated O^{2-} and Zn^{2+} ions, stacked alternatively along the *c*-axis (Supplementary Fig. 3a, top). When subjected to axial stress/strain, piezoelectric polarization charges are generated at both surfaces within thickness of one to two atomic layers of the crystal along certain orientations. Simply, metal cations Zn^{2+} and anions O^{2-} are tetrahedral coordinated, in which the centers of positives ions and negative ions are overlapped with each other at strain free case. Upon a stress/stain along an apex of the tetrahedron, the centers of Zn^{2+} cations and O^{2-} anions are relatively displaced, which results in an electrical dipole moment (hence a piezoelectric polarization) (Supplementary Fig. 3a, bottom). Since all units in ZnO have such a dipole moment under stress/strain, an electrical potential distribution called the piezopotential (piezoelectric potential) is macroscopically generated along the stress/strain direction (Supplementary Fig. 3b). This is the piezoelectricity of ZnO along *c*-axis which is the growth direction of our ZnO micro/nano-wire (Supplementary Fig. 3c). The piezoelectricity is the key factor

that directly determines the performance of the piezoelectric tunneling junction. Here, we used piezoresponse force microscopy (PFM) to investigate the piezoelectricity of ZnO microwire (Supplementary Fig. 3d). As expected, obvious amplitude changes were observed as the tip voltage increased continuously. From the slope of the PFM amplitude versus the applied voltage curve, the PFM measurements reveal that the piezoelectric coefficient (d_{33}) of ZnO microwire is ~11 pm V^{-1}, which is a relatively high value among some piezoelectric materials[2-8]. Coupling the piezoelectricity and semiconducting property, the ZnO microwire is suitable for the piezotronic strain sensors.

The ZnO used in our experiments were oriented along the *c*-axis. When an external strain (tensile strain or compressive strain) is applied on the device, piezoelectric charges will generate at the ±(0001) facet of ZnO, as shown in Supplementary Fig. 4a. The piezopotential can serve as the driving force for the flow of electrons in the external load once subject to mechanical deformation, which is the fundamental of the piezoelectric nanogenerator. The magnitude and polarity of the piezoelectric charge (or the piezopotential) within the ZnO microwire depend on the crystallographic orientation of the crystal and the magnitude and polarity of the applied strain. Supplementary Fig. 4b-d show the piezoelectric current responses of a single ZnO microwire based nanogenerators under tensile strain and compressive strain, and the related mechanism. The positive current output is first observed with tensile strain applied, and negative output is observed when the strain released (Supplementary Fig. 4b, d). However, the negative current output is first observed with compressive strain applied, and the positive output is observed when the compressive strain is released (Supplementary Fig. 4c). Thus, the sign of the output signal of the nanogenerator under tensile/compressive strain are reversed, so that we can determine the polarity of ZnO microwire and control it based on the signal of the nanogenerators when fabricate the piezoelectric strain sensors. It should be noted that the piezoelectric charge created at the ±(0001) facets plays a dominant role in our work, instead of the average of the multiple interfaces.

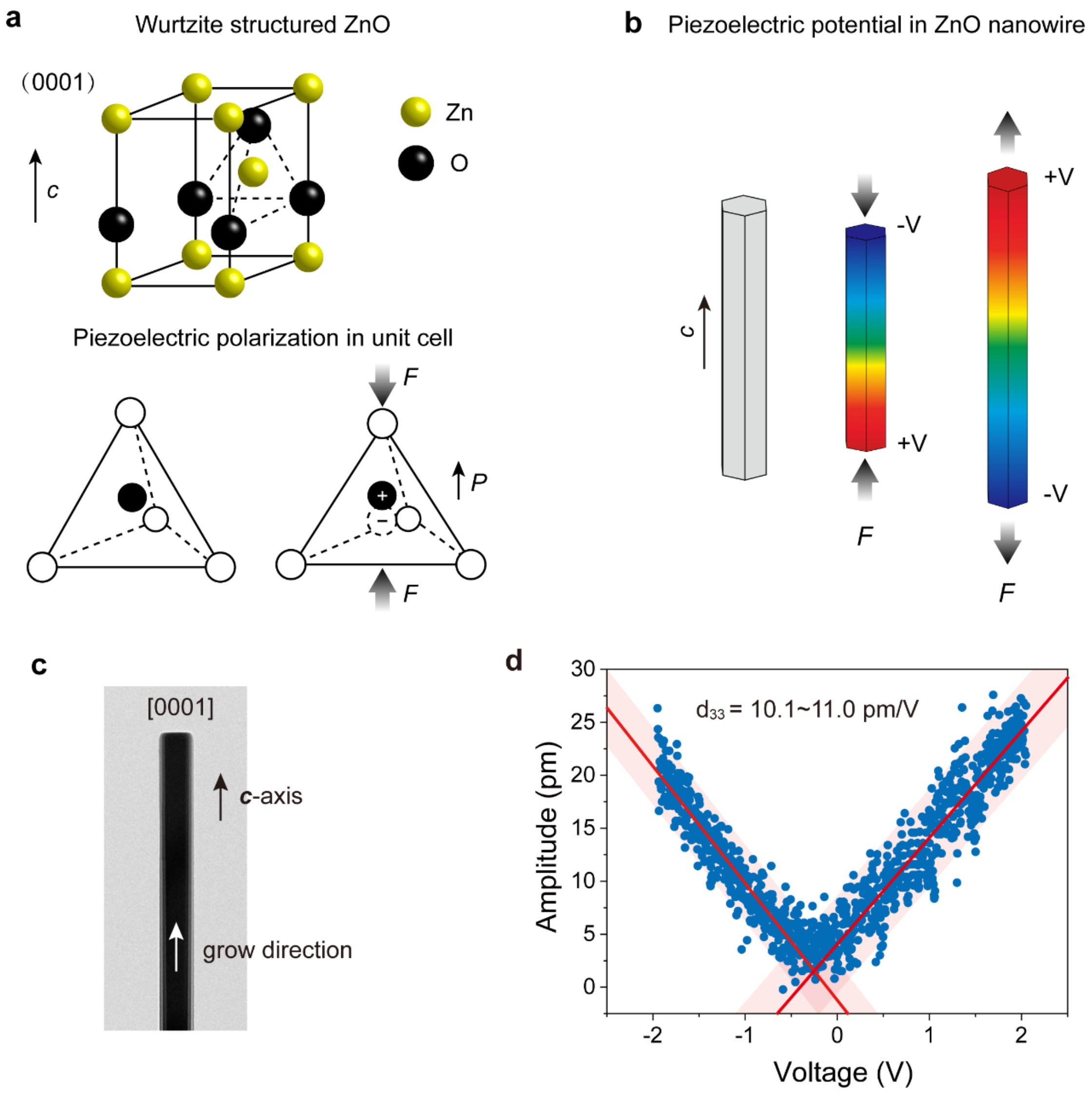

Supplementary Fig. 3 | Piezoelectricity in wurtzite ZnO. a, Atomic model of the wurtzite-structured ZnO and the origin of piezoelectricity. **b,** Piezopotential distributed along ZnO nanowire under axial forces. **c,** Transmission electron microscopy images of ZnO microwire. The white arrow and the black arrow are the growth direction and the piezoelectric polarization axis direction of the micro/nanowire, respectively. **d,** Piezoelectric coefficient (d_{33}) of ZnO micro/nanowire.

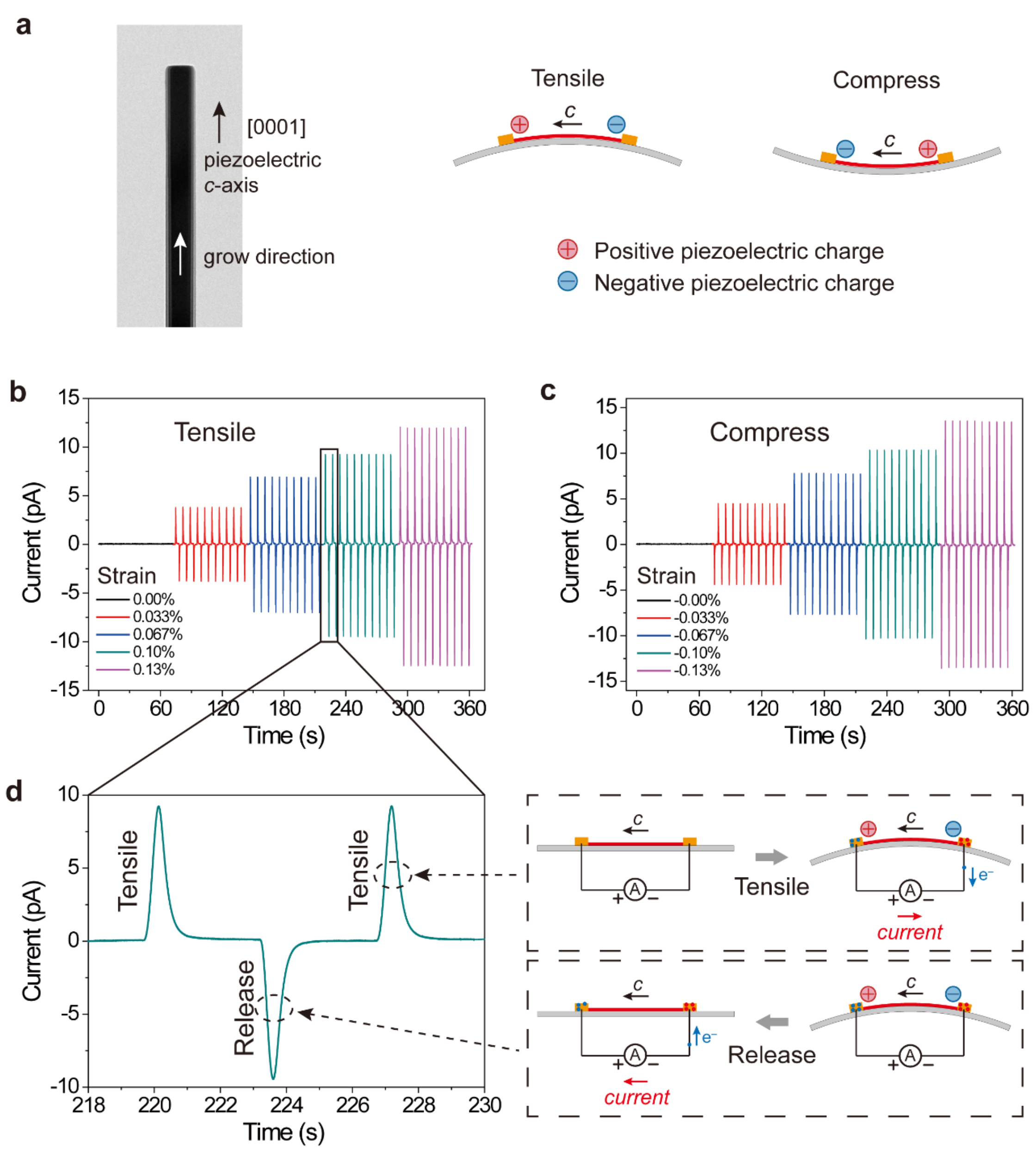

Supplementary Fig. 4 | Piezoelectric nanogenerators under tensile strain and compressive strain to determine the polarity of ZnO microwire. a, Transmission electron microscopy image of ZnO microwire show that the microwire grown along *c*-axis. **b** and **d,** Piezoelectric charges generated at the ±(0001) facets of ZnO nanowire under strain. **c**, Piezoelectric current of nanogenerators under tensile strain and compressive strain and the mechanism.

Supplementary Note 3 | Calculation of the Strain of the Sensors

In our experiment, a thin layer of PDMS was used to encapsulate the device to isolate the air and protect the mechanical properties of the sensors. As shown in Supplementary Fig. 5, one end of the nanowire was fixed, and the other end was free. When the device is strained, the displacement of its free end is M, the distance between the two ends of the device is L, assuming that the bending radius of the strained device is R, which follows the formula[9]:

$$R = (L^2 + 4M^2)/8M \tag{1}$$

Since the Young's modulus of PDMS (360-870 kPa) is much smaller than that of the PET (3-3.5 GPa) flexible substrate, the mechanical properties of PET are not affected by PDMS. After considering that the area and thickness of Ag electrodes and the size of nanowires are all much smaller than those of the PET substrate, we can calculate the strain of the ZnO nanowires based on the strain of the substrate. We set the length of the nanowire to be l, the diameter to be d, and the thickness of the PET substrate to be h. Because $R \gg h$ and $h \gg d$, the strain (ε) of the nanowire can be approximately equivalent to the strain of the outer surface of the substrate, which can be written as:

$$\varepsilon = h/2R = 4Mh/(L^2 + 4M^2) \tag{2}$$

According to the above formula, the strains of the nanowires were calculated to be 0.033%, 0.067% and 0.1% responding to the bending radiuses of 2.25, 1.12 and 0.75 mm, respectively.

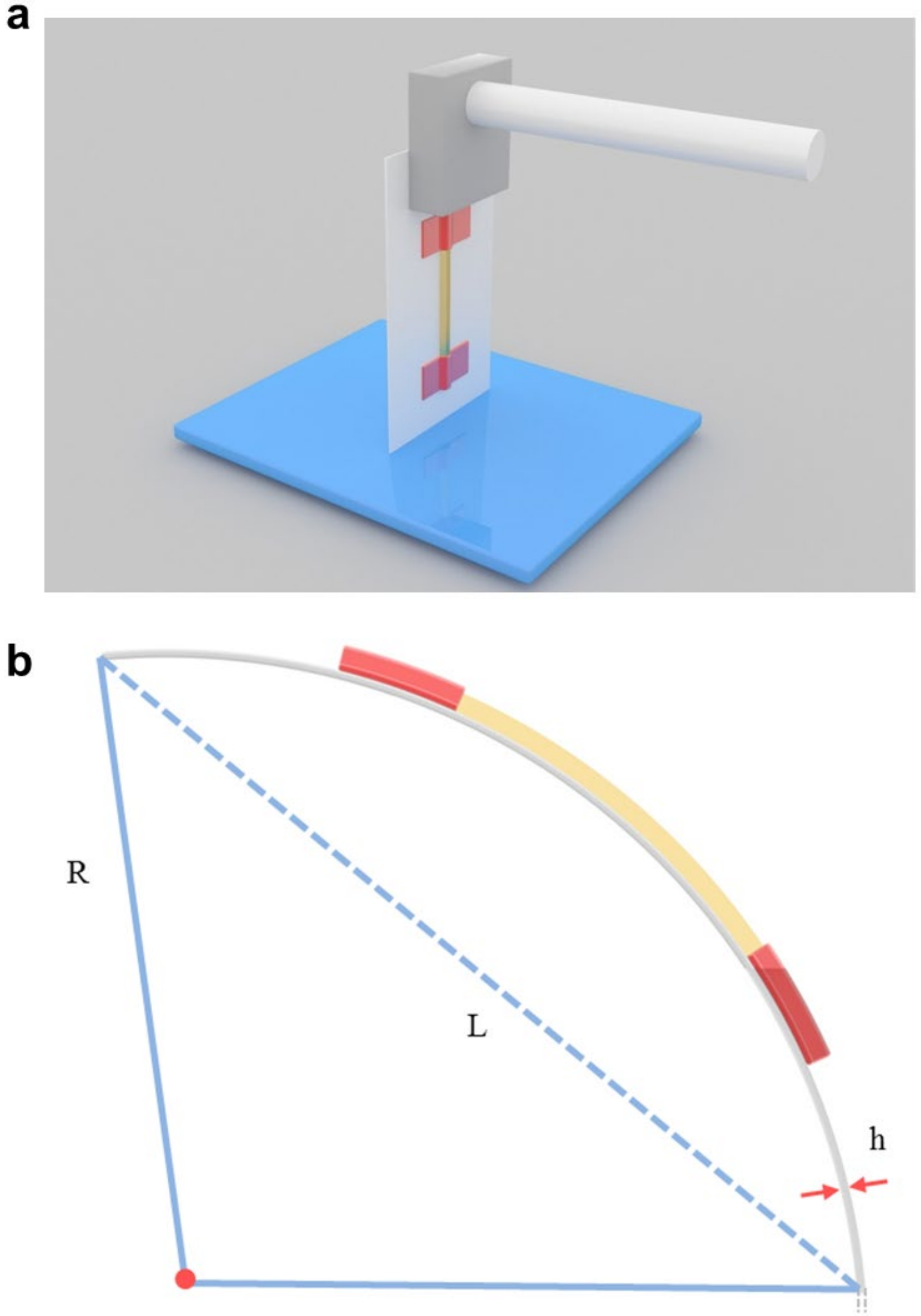

Supplementary Fig. 5 | a, Schematic of the measurement system to characterize the performance of the strain sensor device. **b,** Schematic diagram of the sensor device with a bending radius R.

Supplementary Note 4 | Asymmetry *I-V* Characteristics of the Tunneling Junctions without Strain

The devices with two symmetric Ag electrodes should have the same properties in theory, and hence the current-voltage (*I-V*) characteristics of the device should be symmetrical. However, in the device manufacturing process, it is difficult to ensure that the properties of the electrodes on both sides of the device are completely consistent. Small differences of contacts at two terminals of device will cause asymmetry. This kind of asymmetry have also observed in many literature reports about nanowires[10-14]. This inevitable inconsistency will lead to the asymmetric *I-V* characteristics of the device without strain. Supplementary Fig. 6 gives several *I-V* curves of our devices with the same manufacturing process.

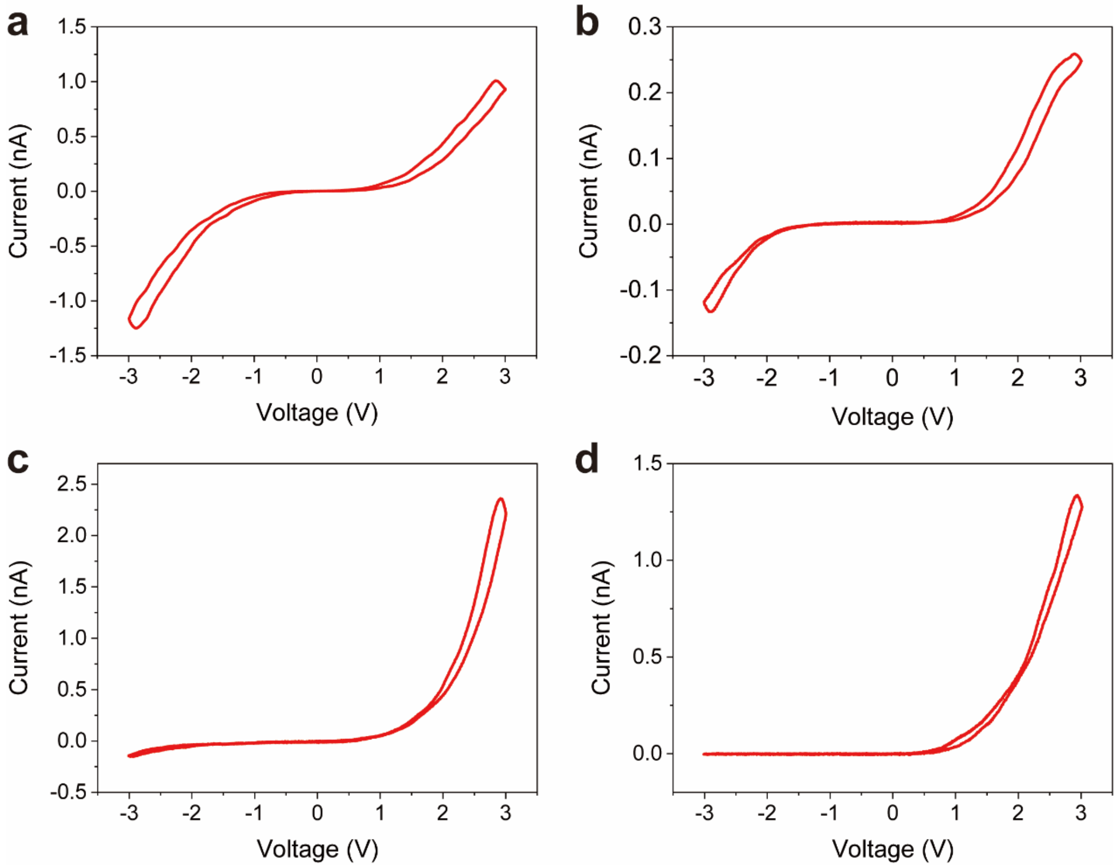

Supplementary Fig. 6 | *I-V* characteristics of Ag/HfO_2/n-ZnO devices. a, Symmetrical *I-V* curve and **b-d,** asymmetrical *I-V* curves of our devices with the same manufacturing process.

Supplementary Note 5 | Interface Traps/Defects in Tunneling Junctions

To verify the influence of defective surface, we carefully characterized the ZnO surface and carried out the capacitance measurement to evaluate the interface trap formed in the tunneling junctions.

To avoid the pollution, the synthesized ZnO nano/microwires are then stored in the inert environment, and are used to make devices within a few days. Supplementary Fig. 1b exhibits the transmission electron microscopy (TEM) images of ZnO nano/microwire, which show its surfaces in detail. The clear lattice profiles indicate that the synthesized ZnO nano/microwires used for MIS tunneling junctions have a good crystallinity and flat surface.

Traditionally, the capacitance measurement can give rapid evaluation of the influence of interface traps on the tunneling property of MIS tunneling junctions[15]. We performed the capacitance-voltage (*C*-*V*) characterizations on 26 devices, as shown in Supplementary Fig. 7. Two typical *C*-*V* curves of Ag/HfO_2/n-ZnO tunneling junctions without strain are observed in the experiments (Supplementary Fig. 7a). According to the research or theory on interface traps (also called interface states, interface defects, surface states and so on in history)[16-19], the interface traps have a very noticeable influence on the *C*-*V* curve, making it to be stretched out in the voltage direction. This is due to the fact that extra charges have to fill the traps, so it takes more total charge or applied voltage to accomplish the same surface potential or band bending. Additionally, the interface traps will also affect the total capacitance of tunneling junction. For a fixed bias, since extra charges will be required to fill the interface traps, the remaining charges to be put in the depletion layer are reduced and this will reduce the band bending or surface potential. So, the basic feature of the *C*-*V* curve with interface traps will shift in the voltage direction and also the capacitance direction, and the curve opening will be widened[15]. Therefore, the blue curve in Supplementary Fig. 7a is close to the ideal *C*-*V* characteristics of MIS tunneling junction with few interface traps[15]; while the red curve is stretched out in the voltage direction, which represents the device with obvious interface traps. Supplementary Fig. 7b counts the characterization results of 26 newly fabricated devices, and it is found that only a few devices (~3.85%) have obvious interface traps.

Combining the above characterization of the surface quality of the ZnO nano/microwires (Supplementary Fig. 1) and the *C*-*V* characteristics of

Ag/HfO_2/n-ZnO devices (Supplementary Fig. 7), the effect of interface trap in our experiment can be negligible.

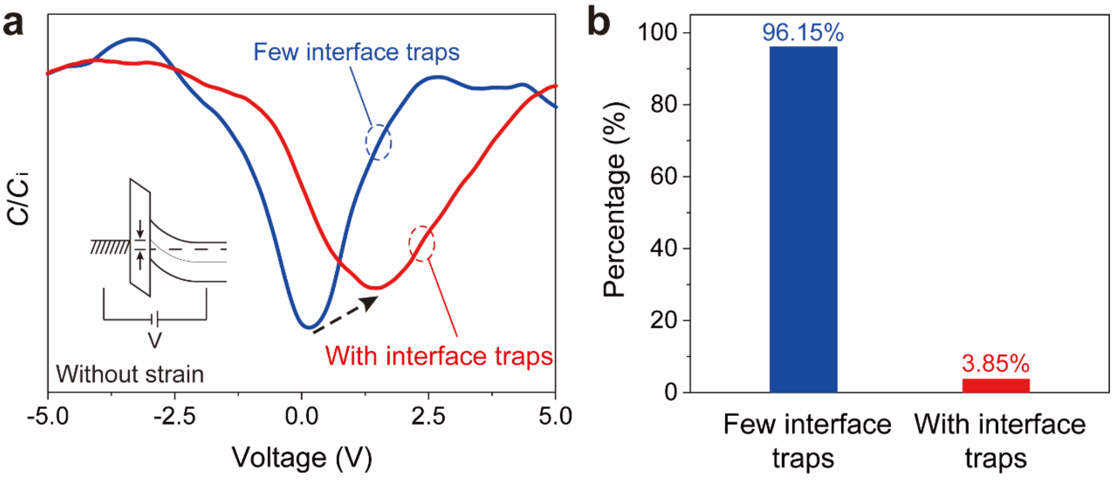

Supplementary Fig. 7 | Influence of interface traps on C-V curves of Ag/HfO_2/n-ZnO tunneling junctions. a, Two typical *C-V* curves of Ag/HfO_2/n-ZnO devices under strain free condition. The voltage is applied to the semiconductor side reference to the metal side. The blue curve is close to the ideal *C-V* characteristics of MIS tunneling junction. The red curve is stretched out in the voltage direction, which represent the device with obvious interface traps. **b,** Percentages of 26 devices with few interface traps and obvious interface traps occurred in experiments.

Supplementary Note 6 | Different Regulation Mechanisms of Piezoelectric Charges and Interface Traps/Defects on Electrical Transport under Strain

We carefully investigate the possible influence of interface traps/defects on the electrical transport of devices, and clarified how to distinguish the piezotronic effect from the influence of interface traps, and finally verified the dominant mechanism of the strain sensor in this work.

(1) Strain-induced symmetric modulation of electrical transport by interface traps/defects

Interface traps have an important influence on the electrical transport of the interface. The introduction of strain will change the interface trap states, such as distribution, thus change the effective resistance and the electrical transport of the interface. In order to clearly show how the interface trap affects the electrical transport of the device under strain, we give schematic diagrams (Supplementary Fig. 8) and discuss the impacts of applied stress on electrical transport under four different cases:

Case 1: (Supplementary Fig. 8a)

In this case, it is assumed that tensile strain increases the interface current, while compressive strain decreases the interface current. When the device is stretched, the contact interfaces on both sides of the device will be subjected to tensile strain. Thus, the current will increase regardless of whether it is forward biased or reverse biased, as illustrated in Supplementary Fig. 8a. It should be noted that even if there is a difference in the contact between the two ends of the device, the forward and reverse currents will still increase, but the magnitude may be different. Under compressive deformation, both the forward and reverse currents will decrease. The simultaneous increase or decrease of forward current and reverse current caused by the strain (tensile or compress) is a symmetrical regulation, which can be easily seen from the *I-V* curves in Supplementary Fig. 8a.

Case 2: (Supplementary Fig. 8b)

It is assumed that tensile strain decreases the interface current, while compressive strain increases the interface current. In this case, the tensile strain will make the forward and reverse currents both decrease, while the compressive strain will make the forward and reverse currents both increase. The strain-controlled *I-V*

curves can also be schematically plotted in Supplementary Fig. 8b. This kind of regulation of strain on the interface trap and the electrical transport of the device is still a symmetric regulation.

Case 3: (Supplementary Fig. 8c)

It is assumed that both the tensile strain and the compressive strain will increase the interface carrier transport. In this case, the current of the device will increase no matter it is subjected to tensile strain or compressive strain. The corresponding *I-V* curves controlled by strain are illustrated in Supplementary Fig. 8c, also indicating a symmetric regulation.

Case 4: (Supplementary Fig. 8d)

It is assumed that both the tensile strain and the compressive strain will decrease the interface carrier transport. Similar to case 3, the forward and reverse currents will both decrease whether the device is tensile strained or compressive strained. The *I-V* curve is also regulated symmetrically by the applied strain, as schematically diagramed in Supplementary Fig. 8d.

Through the above discussion, we can see that strain-induced modulation of electrical transport by interface traps is a symmetric effect.

(2) Strain-induced asymmetric modulation of electrical transport by piezoelectric charges (piezotronic effect)

The piezotronic effect is an interface effect that asymmetrically modulates local contacts at different terminals of the device[20], as schematically illustrated in the following Supplementary Fig. 9. Tunneling junctions will be formed at local contacts at different terminals of the device without strain (Supplementary Fig. 9a, left). Due to the accidental error/uncertainty of device manufacturing, the tunneling junctions (including barrier height and width) at both ends will be slightly different.

When subjecting to a tensile strain (Supplementary Fig. 9a, middle), positive piezoelectric polarization charges are produced at the surface of the ZnO nano/microwire (assuming its piezoelectric *c*-axis points to the left) on the left contact of device, which will reduce the barrier height and width of the tunneling junction; while negative polarization charges are produced on the right contact, leading to an increasement of the barrier height and width of the tunneling junction. When a forward bias (potential of right electrode is higher than that of left electrode, upper middle in Supplementary Fig. 9a) is applied, the electrical transport characteristics are

mainly determined by the tunneling junction on the left terminal, which is reversely biased. Because of the decreased barrier height and width of the left contact, the current flowing through the device will increase and become larger than that of device without strain. Similarly, when the device is reversely biased (potential of right electrode is lower than that of left electrode, lower middle in Supplementary Fig. 9a), under a same bias voltage, the (reverse) current of the device will be smaller than that without strain. Therefore, compared to the case without strain, the forward current of the device with tensile strain will increase; while the reverse current will decrease. With the same principle, we can also analyze the modulation of the current of the device under a compressive strain, as shown in Supplementary Fig. 9a, right. We can see that the compressive strain will cause a decrease of the forward current (upper right in Supplementary Fig. 9a) and an increase of the reverse current (lower right in Supplementary Fig. 9a).

The corresponding *I-V* curves can be schematically plotted in the Supplementary Fig. 9b. The strain-induced modulation of electrical transport by piezoelectric charges (piezotronic effect) is asymmetric. The piezotronic effect modulation (Supplementary Fig. 9) differs from the strain-induced modulation by interface defects/traps (Supplementary Fig. 8), the latter results from a symmetrical change of both ends of device under strain, whereas the piezotronic effect modulation arises as a result of the polarization of nonmobile ions in the piezoelectric crystal[21, 22]. Therefore, the strain-induced modulation of electrical transport by interface defects/traps is a kind of symmetrical effect, whereas the piezotronic effect is an interface effect that asymmetrically regulates local contacts at different terminals of the device because of the polarity of the piezoelectric potential.

Therefore, it is possible to distinguish whether the interface defect/trap effect or the piezotronic effect dominates the electrical transport of device.

(3) Experimental results of strain-controlled I-V curves

In order to determine the dominant mechanism of modulation of device is the piezotronic effect, we measured the strain-controlled *I-V* curves of device under a large strain range (from -2‰ to 2‰) and a small strain range (from -0.1‰ to 0.1‰), as shown in Supplementary Fig. 10.

In the large strain range, an asymmetric modulation of the strain on the *I-V* characteristics of the device can be found whether the direction of the nano/microwire

(device) is reversed (Supplementary Fig. 10a-2) or not (Supplementary Fig. 10a-1). As shown in Supplementary Fig. 10a-1, the forward current of the device increases and the reverse current decreases with the increase of tensile strain; whereas the reverse current of the device increases and the forward current decreases with the increase of compressive strain. With the reverse of the device (Supplementary Fig. 10a-2), we can also see that the strain-controlled *I-V* curves still exhibit asymmetric modulation, indicating that the modulation is related to the orientation of the device (ZnO nano/microwire). This asymmetric modulation corresponding to the polarity of the piezoelectric potential of ZnO nano/microwire is consistent with the result in Fig. 2c of manuscript. Therefore, in a large strain range, piezotronic effect (Supplementary Fig. 9) plays a dominant role in the regulation of electrical transport of device in this work.

Similarly, we also observed the asymmetric modulations of *I-V* curves by the applied strain in a small range in Supplementary Fig. 10b, which indicates that the piezotronic effect is still the dominant control mechanism at small strains, rather than the effect of interface defects/traps.

Furthermore, as a control experiment, we also measured the *I-V* characteristics of Ag/HfO_2/n-Si tunneling junction under small strain ranged from -0.033‰ to 0.033‰. Supplementary Fig. 11 gives the comparison of the *I-V* curves of the Ag/HfO_2/n-ZnO tunneling junction (Supplementary Fig. 11a) and Ag/HfO_2/n-Si tunneling junction (Supplementary Fig. 11b). When under very small strain, the *I-V* curves of the tunneling junction composed of non-piezoelectric semiconductor (n-Si) does not change much with the strain, while the one composed of piezoelectric semiconductor (n-ZnO) changes obviously, which also exhibits asymmetric modulation. Thus, the strain-induced piezoelectric potential plays a vital role in regulation of electrical transport of tunneling junction.

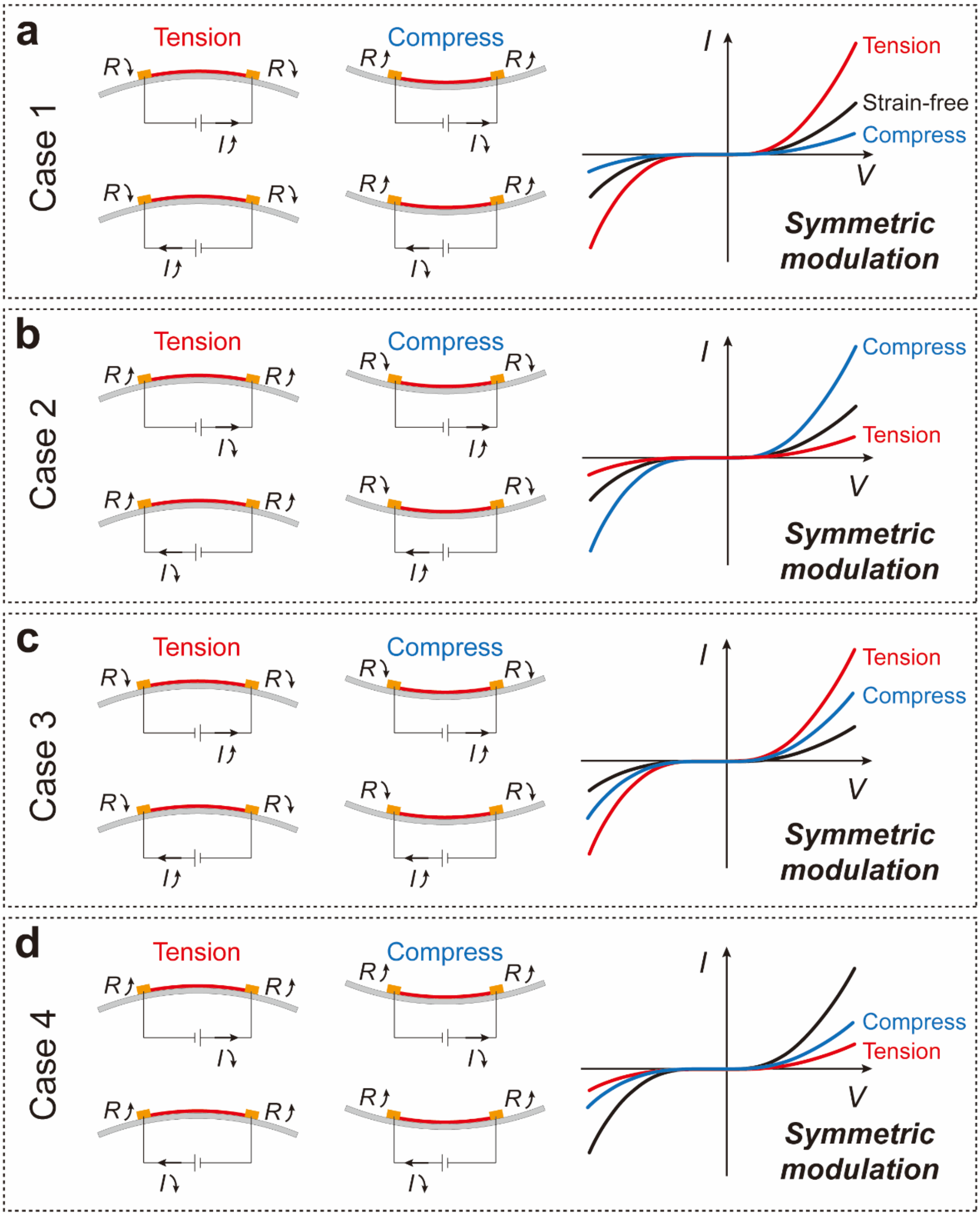

Supplementary Fig. 8 | Strain-induced symmetric modulation of electrical transport by interface traps/defects. a-d, The control mechanisms of strain on *I-V* characteristics under four different situations. Case 1 assumes that the tensile and compressive stress will respectively decrease and increase the interface resistance by changing the state of the interface trap. Case 2 assumes the tensile and compressive stress will respectively increase and decrease the interface resistance. Case 3 assumes the tensile and compressive stress both decrease the interface resistance. Case 4 assumes the tensile and compressive stress both increase the interface resistance.

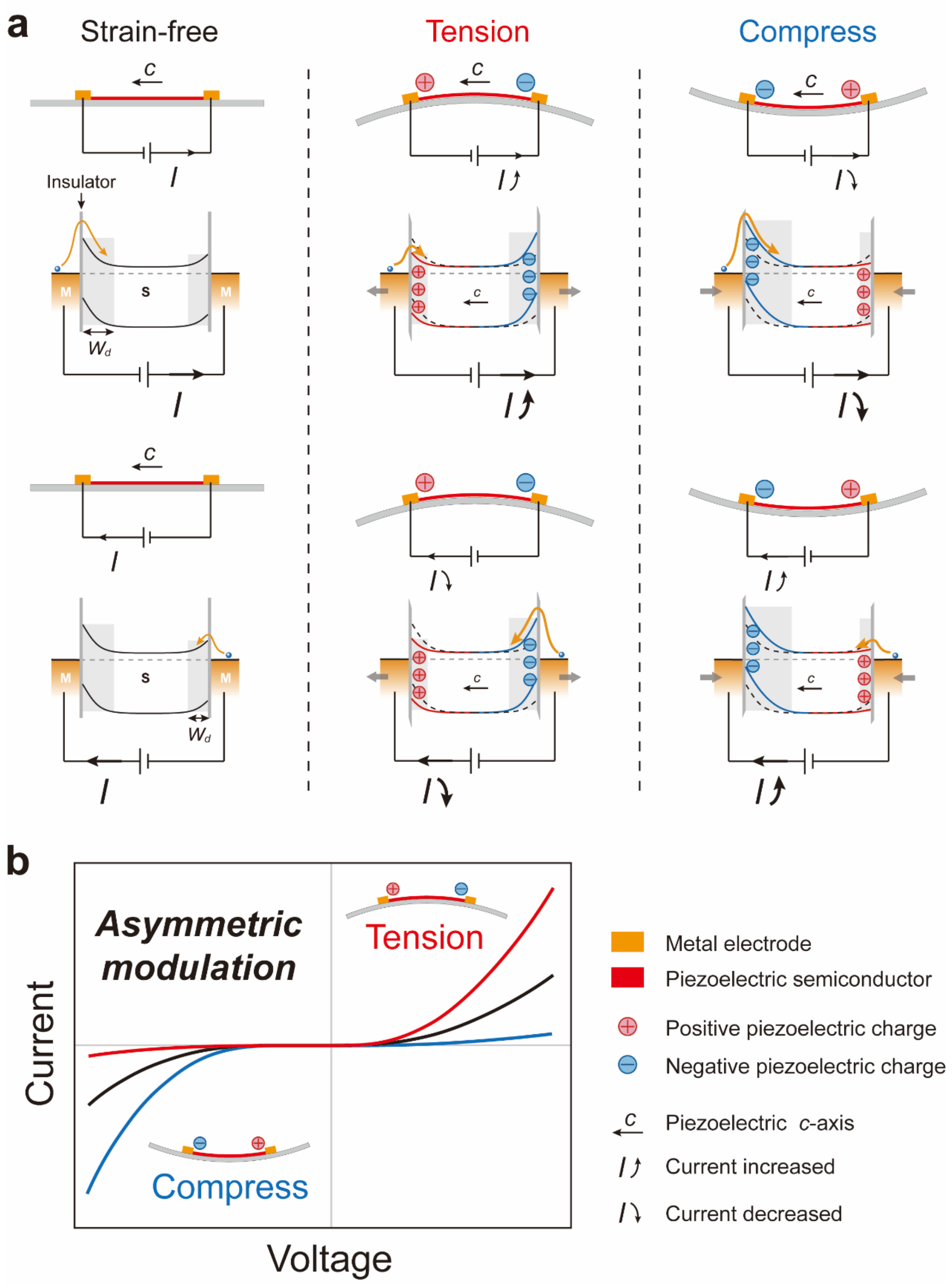

Supplementary Fig. 9 | Strain-induced asymmetric modulation of electrical transport by piezotronic effect. a, Schematic diagrams of bending situations and corresponding energy bands of device with strain-free (left), tensile strain (middle) and compressive strain (right). **b,** Schematic illustrations of the *I-V* curves of the device under no strain (black), tensile strain (red) and compressive strain (blue), which indicate an asymmetric modulation of electrical transport by strain.

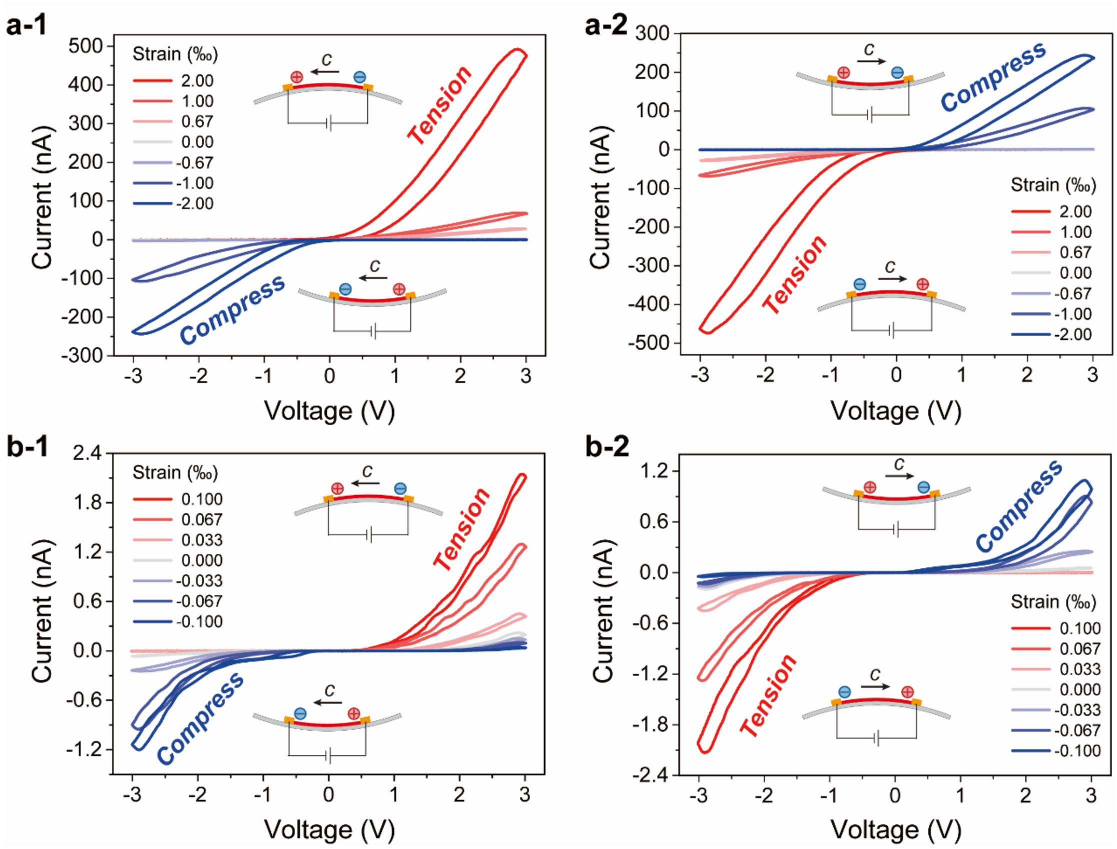

Supplementary Fig. 10 | Experiment results of *I-V* characteristics modulated by strain. a, The *I-V* curves of the devices with piezoelectric *c*-axis (**a-1**) parallel and (**a-2**) opposite to external electric field under a large strain (ranged from -2‰ to 2‰). **b,** The *I-V* curves of the devices with piezoelectric *c*-axis (**b-1**) parallel and (**b-2**) opposite to external electric field under a small strain (ranged from -0.1‰ to 0.1‰).

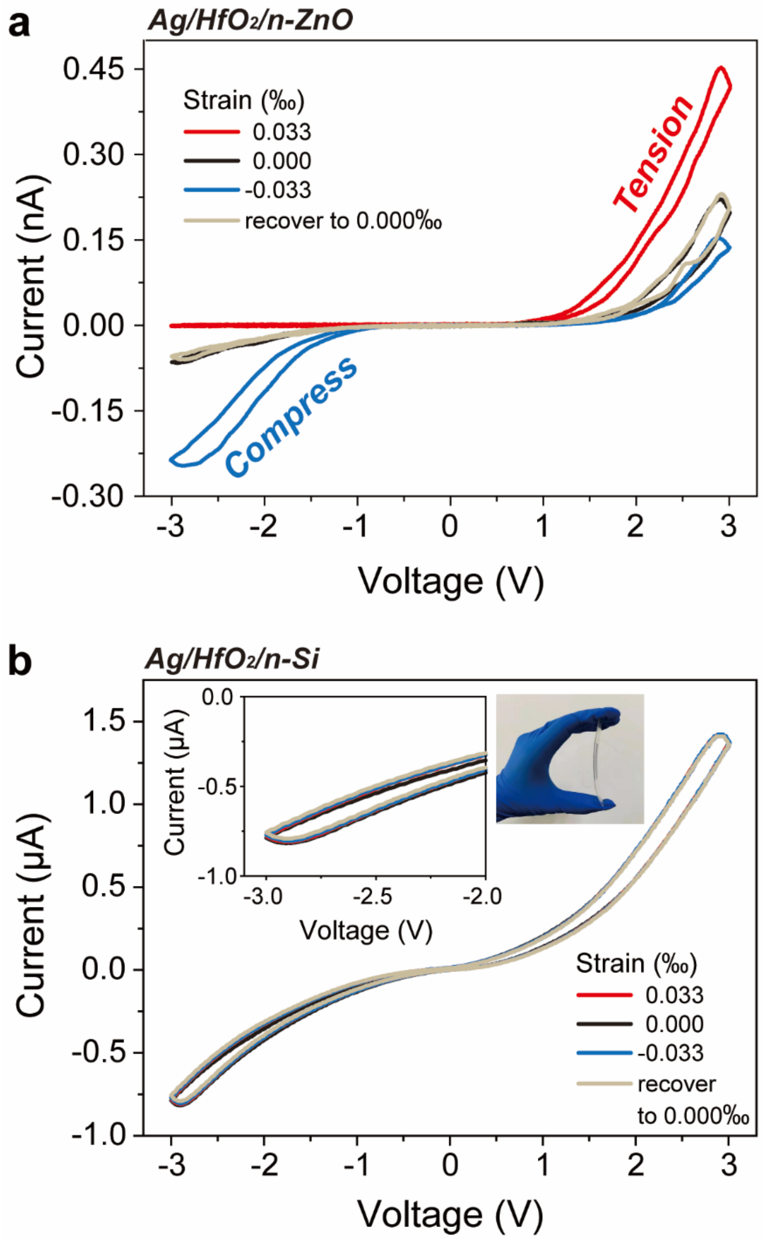

Supplementary Fig. 11 | Comparison of the strain-tuned I-V characteristics of two terminal devices with contact of Ag/HfO_2/n-ZnO and Ag/HfO_2/n-Si. a, Strain-controlled *I-V* curves of Ag/HfO_2/n-ZnO device under a small strain, indicating an asymmetric modulation. **b,** *I-V* curves of Ag/HfO_2/n-Si device under a small strain, indicating an unobvious regulation.

Supplementary Note 7 | Influence of the Ag/n-ZnO Contact Area of the Side Surface on the Performance of the Strain Sensor

As shown in Supplementary Fig. 12a, we assume that the radius of the end surface of the ZnO nanowire is r, and the length of the side contact area between ZnO nanowire and metal electrode is L'. So, the areas of the end surface (S_1) and the side surface (S_2) can be written as,

$$S_1 = \pi r^2$$
$$S_2 = 2\pi r L'$$

(3)

Since the Schottky barriers formed at the end surface and the side surface are in parallel with each other, the equivalent circuit can be illustrated as the Supplementary Fig. 12b, and the total current flowing through the device can be write as

$$I = I_1 + I_2$$

(4)

where I_1 and I_2 represent the currents flowing through the end surface and the side surface, respectively. Based on the Schottky theory[15], we can obtain the device current ($V > 0.026$ V) through the following formula, when the device is in strain-free,

$$I_1 = S_1 A^{**} T^2 e^{-\frac{q\varphi_{\mathrm{Bno}}}{kT}} e^{\frac{qV}{kT}}$$
$$I_2 = S_2 A^{**} T^2 e^{-\frac{q\varphi_{\mathrm{Bno}}}{kT}} e^{\frac{qV}{kT}}$$
$$I = (S_1 + S_2) A^{**} T^2 e^{-\frac{q\varphi_{\mathrm{Bno}}}{kT}} e^{\frac{qV}{kT}}$$

(5)

where, A^{**} is the effective Richardson constant, φ_{Bn0} is the Schottky barrier height under strain-free state, q is the electron charge, k is Boltzmann constant, T is the absolute temperature, and V is the applied voltage.

As a strain is applied to the ZnO nanowire, piezoelectric polarization charges will be generated at the end surface to modulate the Schottky barrier height. It is worth noting that the Schottky barrier formed at the side surface will be less influenced by the piezoelectric polarization charges. Thus, the strain modulated current can be written as[23]

$$I_{1,\mathrm{strain}} = S_1 A^{**} T^2 e^{-\frac{q(\varphi_{\mathrm{Bno}} + \Delta\varphi_{\mathrm{piezo}})}{kT}} e^{\frac{qV}{kT}}$$
$$I_{2,\mathrm{strain}} = S_2 A^{**} T^2 e^{-\frac{q\varphi_{\mathrm{Bno}}}{kT}} e^{\frac{qV}{kT}}$$

$$I_{\text{strain}} = I_{1,\text{strain}} + I_{2,\text{strain}} = \left(S_1 e^{-\frac{\Delta\varphi_{\text{piezo}}}{kT}} + S_2\right) A^{**}T^2 e^{-\frac{q\varphi_{\text{Bn0}}}{kT}} e^{\frac{qV}{kT}}$$

(6)

where $\Delta\varphi_{\text{piezo}}$ represents the actual change of the Schottky barrier height of the ZnO end surface.

However, in the experiment, we did not consider the influence of the side surface and mistakenly thought that the measured current was:

$$I'_{\text{strain}} = (S_1 + S_2)A^{**}T^2 e^{-\frac{q(\varphi_{\text{Bn0}} + \Delta\varphi'_{\text{piezo}})}{kT}} e^{\frac{qV}{kT}}$$

(7)

where $\Delta\varphi'_{\text{piezo}}$ is the effective change of the Schottky barrier height.

By setting I_{strain} equal to I'_{strain}, we can build the relationship between $\Delta\varphi_{piezo}$ (the actual value of the change of the Schottky barrier height) and $\Delta\varphi'_{piezo}$ (the calculated value of the change of the Schottky barrier height from experimental data without consideration of the influence of the side surface), and evaluate $\Delta\varphi_{piezo}$ by $\Delta\varphi'_{\text{piezo}}$.

$$\left(S_1 e^{-\frac{\Delta\varphi'_{\text{piezo}}}{kT}} + S_2\right) A^{**}T^2 e^{-\frac{q\varphi_{\text{Bn0}}}{kT}} e^{\frac{qV}{kT}} = (S_1 + S_2)A^{**}T^2 e^{-\frac{q(\varphi_{Bn0} + \Delta\varphi_{piezo})}{kT}} e^{\frac{qV}{kT}}$$

$$\Rightarrow \; S_1 e^{-\frac{q\Delta\varphi'_{\text{piezo}}}{kT}} + S_2 = (S_1 + S_2) e^{-\frac{q\,\Delta\varphi_{\text{piezo}}}{kT}}$$

$$\Rightarrow \; \frac{S_1}{S_2} e^{-\frac{q\Delta\varphi'_{\text{piezo}}}{kT}} + 1 = \left(\frac{S_1}{S_2} + 1\right) e^{-\frac{q\,\Delta\varphi_{\text{piezo}}}{kT}}$$

(8)

Due to

$$\frac{S_1}{S_2} = \frac{\pi r^2}{2\pi r L'} = \frac{r}{2L'} \approx \frac{4\ \mu\text{m}}{2 \times 100\ \mu\text{m}} = 0.02$$

(9)

we can obtain the following relationship,

$$e^{-\frac{q\Delta\varphi_{\text{piezo}}}{kT}} \approx \left(0.02 \times e^{-\frac{q\Delta\varphi'_{\text{piezo}}}{kT}} + 1\right)/1.02$$

(10)

For the generated positive piezoelectric polarization charge, $\Delta\varphi'_{\text{piezo}} < 0$.

Through analysis, we can estimate the change in the height of the Schottky barrier on the end surface by $\Delta\varphi'_{\text{piezo}}$:

(i) If we obtain the $q\Delta\varphi'_{\text{piezo}} = -kT = -26\,\text{meV}$, the actual change in the height of the Schottky barrier $q\Delta\varphi_{\text{piezo}} \approx -133.8\,\text{meV}$;

(ii) According to the data analyzed in Fig. 2f in the manuscript, under the tensile strain of 0.1%, $\Delta\varphi'_{\text{piezo}} = -7\,\text{meV}$, then the actual change in the height of the Schottky barrier $\Delta\varphi'_{\text{piezo}} \approx -92\,\text{meV}$. This value of approximately $100\,\text{meV}$ is consistent with previous studies in magnitude[24, 25].

In other words, the presence of the side contact reduces the performance of the strain sensor, resulting in an underestimated value of the change of SBH in manuscript. Therefore, in our devices, the piezotronic modification of carrier transport should be stronger.

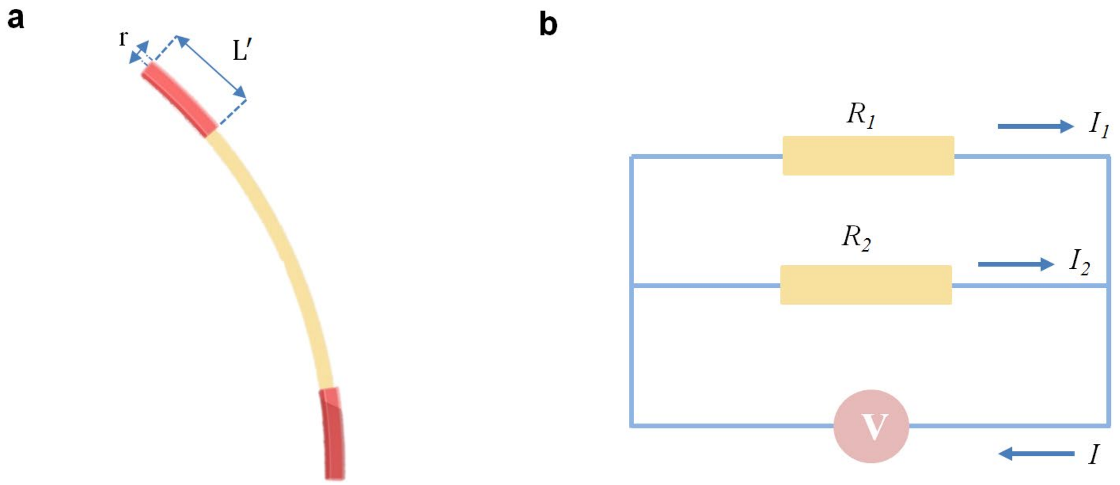

Supplementary Fig. 12 | The Ag/n-ZnO contacts at end/side surfaces and the equivalent circuits. a, The side and end surfaces of ZnO nanowire in contact with metal electrodes. **b,** The equivalent circuit formed by the end surface contact resistance R_1 and the side surface contact resistance R_2.

Supplementary Note 8 | Electrical Transport of the Ag/n-ZnO Schottky-Junction-Based Strain Sensor (SSS)

In order to obtain the carrier transport characteristics of the Schottky-junction-based strain sensor, Poisson's equation, current transports equation, continuity equation, and the piezoelectric equation are used for simple derivation [15, 26-29].

The piezoelectric behavior of the material with piezoelectric properties is described by the polarization vector P. When the piezoelectric material is subjected to a small uniform mechanical strain S_{jk}, the polarization vector P can be expressed as $(P)_{\mathrm{ijk}} = (e)_{\mathrm{ijk}}(S)_{\mathrm{ijk}}$. The third-order tensor $(e)_{\mathrm{ijk}}$ represents the piezoelectric tensor. According to the traditional piezoelectric theory and elastic theory[29, 30], the composition equation can be written as

$$\begin{cases} \sigma = c_{\mathrm{E}}S - e^{T}E \\ D = eS + kE \end{cases} \tag{11}$$

where σ represents the stress tensor, c_{E} is the elasticity tensor, E represents the electric field, D is the electric displacement, and k is the dielectric tensor.

In this work, the device structure of Ag/n-ZnO strain sensor is the conventional metal-semiconductor-metal (MSM) contact piezotronic transistor, consisting of two back-to-back Schottky diodes and a ZnO resistor as shown in Supplementary Fig. 13. In the presence of Schottky barriers, the charge distribution of the MS contact can be simplified as shown in Supplementary Fig. 14a. For simplicity, the surface states and other anomalies are ignored. Upon strain, the piezoelectric charge generated at the interface will change the height of the Schottky barrier. Unlike the method of changing the height of the Schottky barrier by introducing a dopant on the semiconductor side, for the fabricated device, the piezoelectric potential can be continuously adjusted by strain.

For ZnO nanowires grown along the *c*-axis in Ag/n-ZnO contact, the piezoelectric coefficient matrix is expressed as

$$(e)_{\mathrm{ijk}} = \begin{bmatrix} 0 & 0 & 0 & 0 & e_{15} & 0 \\ 0 & 0 & 0 & e_{15} & 0 & 0 \\ e_{31} & e_{31} & e_{33} & 0 & 0 & 0 \end{bmatrix} \tag{12}$$

The current density follows the formula:

$$J = J_{\mathrm{D0}} \exp\left(\frac{q^2 \rho_{\mathrm{piezo}} W_{\mathrm{piezo}}^2}{2\varepsilon_{\mathrm{S}} kT}\right)\left[\exp\left(\frac{qV}{kT}\right) - 1\right]$$

(13)

where J represents the current density, q is the absolute value of the unit electronic charge, ρ_{piezo} is the density of the polarization charge (in the unit of electronic charge), W_{piezo} is the width of the piezoelectric charge distribution on the junction interface, ε_{S} is the permittivity of the piezoelectric semiconductor material, and k is the Boltzmann constant, T is the absolute temperature, and V is the applied voltage. J_{D0} represents the saturation current density of the MSM piezotronic transistor without applied strain, which can be obtained from this formula[23]:

$$J_{\mathrm{D0}} = \frac{q^2 D_{\mathrm{n}} N_{\mathrm{C}}}{kT} \sqrt{\frac{2qN_D(\psi_{\mathrm{bi0}} - V)}{\varepsilon_{\mathrm{S}}}} \exp\left(-\frac{q\varphi_{\mathrm{Bn0}}}{kT}\right)$$

(14)

where D_n is the electron diffusion coefficient, N_C is the effective density of states in the conduction band, N_D is the donor concentration, ψ_{bi0} and φ_{Bn0} are the built-in potential and Schottky barrier height when there is no piezoelectric charge.

The Schottky barrier height under a mechanical deformation can be written as:

$$\varphi_{\mathrm{Bn}} = \varphi_{\mathrm{Bn0}} - \frac{q^2 \rho_{\mathrm{piezo}} W_{\mathrm{piezo}}^2}{2\varepsilon_{\mathrm{S}}}$$

(15)

The *I-V* characteristics of piezotronic transistor based on the MSM structure can be solved by Kirchhoff's law, which have typical nonlinear characteristics and can be tuned by applied strain. Assuming that the resistance of the nanowire is ignored, the carrier transport in the junction region is mainly affected by the height of the Schottky barrier. After setting the cross-sectional area of the MSM piezotronic strain device as S, and the approximate solution of the above equation can be obtained by Taylor series:

$$I(V) \approx I_{\mathrm{D0}} \exp\left(\frac{q^2 \rho_{\mathrm{piezo}} W_{\mathrm{piezo}}^2}{2\varepsilon_{\mathrm{S}} kT}\right)\left[\frac{1}{6}\left(\frac{qV}{kT}\right)^3 + \frac{1}{2}\left(\frac{qV}{kT}\right)^2 + \frac{qV}{kT}\right]$$

(16)

If the strain produced by ZnO nanowires is the strain S_{33} along the *c*-axis, the piezoelectric polarization can be expressed as:

$$P_{33} = e_{33} S_{33} = q\rho_{\mathrm{piezo}} W_{\mathrm{piezo}}$$

(17)

For wurtzite ZnO nanowires with the piezoelectric constant e_{33} is 1.22 C/m^2, the relative dielectric constant is 8.91, the width of piezoelectric charges distribution at the interface is 0.25 nm, and the current density can be written as:

$$I(V) \approx I_{D0}\exp\left(\frac{qe_{33}S_{33}W_{\text{piezo}}}{2\varepsilon_S kT}\right)\left[\frac{1}{6}\left(\frac{qV}{kT}\right)^3+\frac{1}{2}\left(\frac{qV}{kT}\right)^2+\frac{qV}{kT}\right]$$

(18)

where $I_{D0} = J_{D0}S$. I_{D0} is the saturation current without external strain, closely related to the material and structure of the device. And the temperature T is 300 K.

This means that the current transmitted through the Schottky junction has an exponential relationship with the local piezoelectric charge and is closely related to the applied strain. The transported current can not only be adjusted by the magnitude of strain, but also can be effectively tuned by the state of strain (tensile versus compressive). This is the mechanism of the Ag/n-ZnO Schottky-junction-based strain sensor.

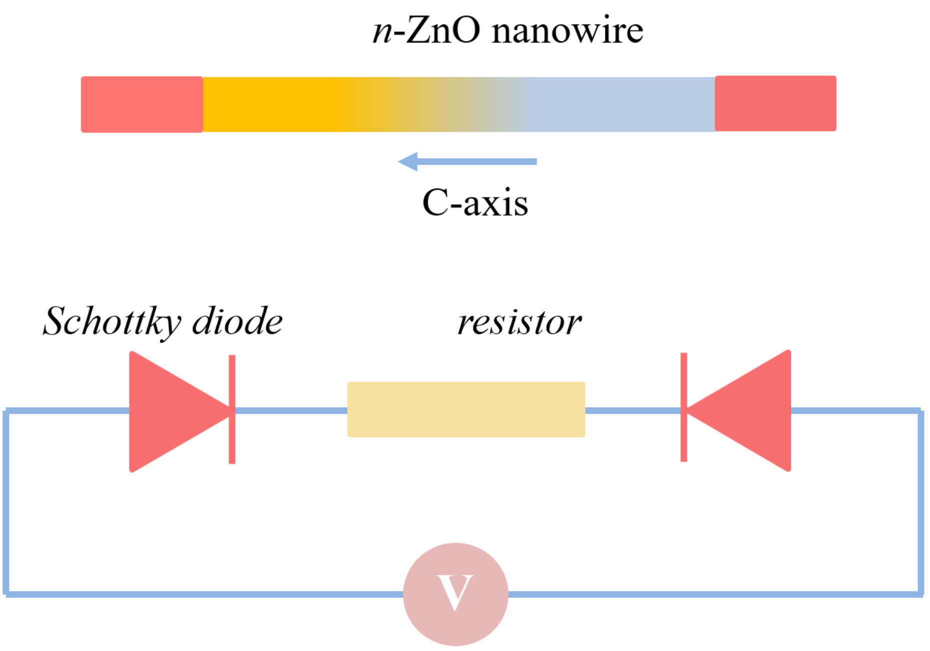

Supplementary Fig. 13 | Schematic of a metal-semiconductor-metal (MSM) piezotronic transistor and equivalent circuit of MSM piezotronic transistor.

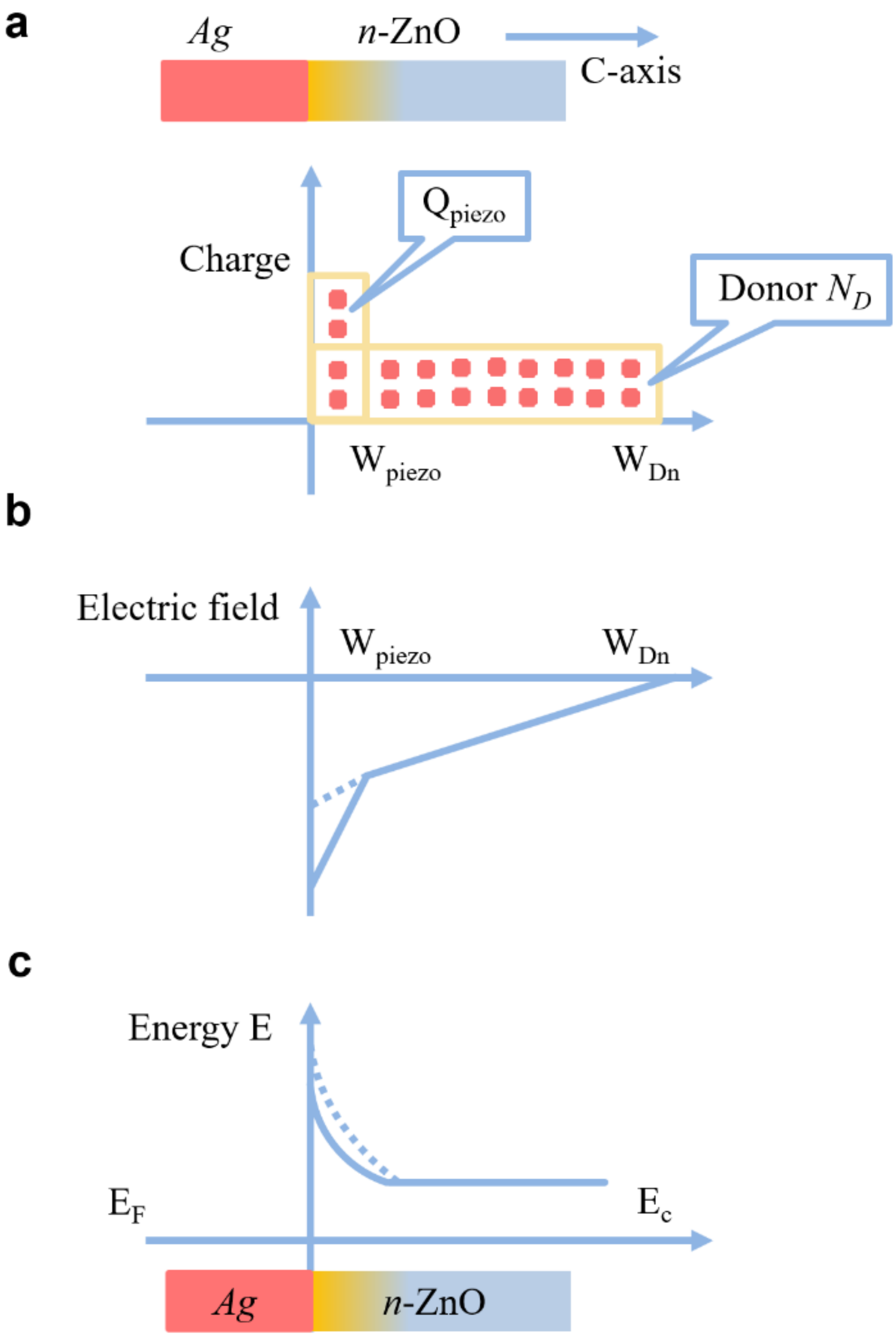

Supplementary Fig. 14 | Ideal metal-semiconductor Schottky contacts with the presence of piezoelectric charges at an applied voltage *V*=0 (thermal equilibrium). **a,** Space charge distribution. **b,** Electric field and **c,** energy band diagram in the presence of piezoelectric charges. Dashed lines indicate the electric field and energy band in the absence of piezoelectric charges and the solid lines indicate the cases when a piezopotential is present in the semiconductor.

Supplementary Note 9 | Calculation of the Change of Schottky Barrier Height and Its Linear Relationship with Strain

The band profile at the interface of the III-V group semiconductor-metal contact can be changed by strain induction. The height of Schottky barrier can be controlled by the piezotronic effect. This is mainly since the piezoelectric polarization charge can move the local Fermi level and change the local conduction band profile. The existence of the Schottky barrier at the MS contact interface plays a vital role in determining the electrical transport properties of the MSM structure.

Studies have shown that most of the potential drop occur at the reverse-biased Schottky barrier[11]. This can determine the I-V characteristic of the piezotronic device. According to the classic thermionic emission-diffusion theory (for $V \gg 3kT/q{\sim}77\ mV$), the current flowing through the reverse biased Schottky barrier is given by the following equation[15]:

$$I = SA^{**}T^2 \exp\left(-\frac{\varphi_\mathrm{S}}{kT}\right) \exp\left(\frac{\sqrt[4]{q^7 N_\mathrm{D}(V + V_\mathrm{bi} - kT/q)/(8\pi^2\varepsilon_\mathrm{S}^3)}}{kT}\right) \tag{19}$$

where S is the area of the Schottky barrier, A^{**} is the effective Richardson constant of the ZnO NW, φ_S is the the reverse-biased Schottky barrier, q is the electron charge, k is Boltzmann constant. N_D is the donor impurity density, V_bi is the build-in potential at the junction barrier, and ε_S is the permittivity of ZnO.

The variation of the Schottky barrier heights $\left(\Delta\varphi_\mathrm{piezo}\right)$ at MS contact induced by the piezoelectric polarization charges is expressed as[31, 32]:

$$\ln[I_\mathrm{strain}/I_\mathrm{free}] \sim \Delta A^{**}/A^{**} - \Delta\varphi_\mathrm{piezo}/kT \tag{20}$$

Under small deformation, we neglect ΔA^{**}, and assume S, A^{**}, T, and N_D are to be known. With consideration that our measurements were made at room temperature, we can have

$$\Delta\varphi_\mathrm{piezo} = -kT\ln[I_\mathrm{strain}/I_\mathrm{free}] \tag{21}$$

where I_strain and I_free are the current measured with and without being strained, respectively.

Based on the previous reports, the strain induced SBH change ($\Delta\varphi_{\text{piezo}}$) is linearly dependent on the piezoelectric charge density ρ_{piezo} .

$$\Delta\varphi_{\text{piezo}} \approx -\frac{q\rho_{\text{piezo}}W_{\text{piezo}}^2}{2\varepsilon_{\text{S}}}$$

(22)

As the normal strain ε_{33} is applied along the *c*-axis of ZnO nanowire in our experiment, the $\Delta\varphi_{\text{piezo}}$ can be also expressed as the equation:

$$\Delta\varphi_{\text{piezo}} \approx -\frac{q\rho_{\text{piezo}}W_{\text{piezo}}^2}{2\varepsilon_{\text{S}}} = -\frac{qe_{33}\varepsilon_{33}\,W_{\text{piezo}}}{2\varepsilon_{\text{S}}}$$

(23)

So,

$$\Delta\ln(I) \propto \Delta\varphi_{\text{piezo}} \propto \rho_{\text{piezo}} \propto \text{strain}$$

(24)

Supplementary Note 10 | Calculation of the Gauge Factor and the Current On-Off Ratio

As the most important parameter, the gauge factor of the ZnO-based strain sensor under compressive and tensile strain can be calculated using the following equation, which can be generally defined as the ratio of relative change in electrical resistance or current to the mechanical strain based on the electromechanical performance[33, 34].

$$\text{guage factor} = \frac{\Delta R/R_{\text{free}}}{\Delta \varepsilon} = \frac{R_{\text{strain}} - R_{\text{free}}}{R_{\text{free}}\Delta \varepsilon} \tag{25}$$

Or, we also write the definition expression with current as output similarly here:

$$\text{guage factor} = \frac{I_{\text{strain}} - I_{\text{free}}}{I_{\text{free}}\Delta \varepsilon} \tag{26}$$

where R_{free} is the initial resistance under no strain, R_{strain} is the resistance at the strain of ε, $\Delta\varepsilon$, I_{strain}, and I_{free} denote the strain applied to the sensor, the final current at the strain of ε, and the initial current with no strain, respectively.

The current on-off ratio reflects the ability of the device to regulate the current, which was defined as the ratio of the current in the on state to the current in the off state of the sensor.

$$\text{on/off ratio} = I_{\text{strain}}/I_{\text{free}} \tag{27}$$

Supplementary Note 11 | Theory of Piezotronic Effect on the Metal-Insulator-Semiconductor Junction

According to the theoretical model coupling with piezotronics, as schematically shown in Supplementary Fig. 15. Along with the applied voltage, there will be a voltage drop on the insulator, and the potential difference of the insulator can be written as

$$\Delta = \frac{E_g}{q} + \chi_S - \phi_m - \phi_p - \psi_S + V \tag{28}$$

here E_g is the semiconductor bandgap, χ_S is the affinity of the semiconductor, ϕ_m represents the metal work function, ϕ_p is the potential difference between the majority-carrier Fermi level and the valence band, ψ_S is the potential across the semiconductor and V represents the applied voltage.

For the sake of simplicity, surface conditions, work function differences and other anomalies are ignored. The semiconductor is grounded, and a positive voltage is applied to the metal side. It is assumed that the semiconductor works under thermal equilibrium, and direct tunneling is the main tunneling mechanism at the interface. Therefore, the difference between the quasi-Fermi levels of electrons and holes is ignored. Thus,

$$\Delta = -\psi_S + V \tag{29}$$

Based on the Gauss's law, we can have

$$\Delta = E_i d_i = d_i \frac{Q_M}{\varepsilon_i} \tag{30}$$

where E_i is the electric field inside the insulator, Q_M is the charge on the metal, d_i is the insulator thickness and ε_i is the permittivity of the insulator. For charge neutrality, it is required that

$$Q_M + Q_S + Q_{piezo} = 0 \tag{31}$$

where Q_S is the charge on the semiconductor surface due to the ionized acceptor and Q_{piezo} is the piezoelectric charge. Thus, equations can be given by

$$\Delta = -d_{\mathrm{i}} \frac{\left(Q_{\mathrm{S}} + Q_{\mathrm{piezo}}\right)}{\varepsilon_{\mathrm{i}}}$$

(32)

$$\mathrm{Q}_S = -qN_{\mathrm{A}}W_{\mathrm{DP}}$$

(33)

$$\mathrm{Q}_{\mathrm{piezo}} = q\rho_{\mathrm{piezo}}W_{\mathrm{piezo}}$$

(34)

According to the assumption of the depletion approximation and completed ionization inside the depletion region, the field and potential distribution can be calculated. By solving the one-dimensional Poisson's equation, the potential $\psi_{\mathrm{S}}(x)$ inside the semiconductor as a function of distance can be expressed as

$$\frac{d^2\psi_{\mathrm{S}}(x)}{dx^2} = -\frac{dE}{dx} = -\frac{\rho(x)}{\varepsilon} = -\frac{q\left[p(x) - n(x) - N_{\mathrm{A}}(x) + \rho_{\mathrm{piezo}}(x)\right]}{\varepsilon}$$

(35)

where $\rho(x)$ is the charge density, $N_{\mathrm{A}}(x)$ is the density of the acceptor, $\rho_{\mathrm{piezo}}(x)$ is the density of the piezoelectric charges. By integrating the Poisson equation, we can obtain the electric field distribution inside the semiconductor.

$$E(x) = -\frac{qN_{\mathrm{A}}\left(x - W_{\mathrm{Dp}}\right)}{\varepsilon_{\mathrm{S}}} + \frac{q\rho_{\mathrm{piezo}}\left(x - W_{\mathrm{piezo}}\right)}{\varepsilon_{\mathrm{S}}} \quad (0 \le x \le W_{\mathrm{piezo}})$$

(36)

$$E(x) = -\frac{qN_{\mathrm{A}}\left(x - W_{\mathrm{Dp}}\right)}{\varepsilon_{\mathrm{S}}} \quad \left(W_{\mathrm{piezo}} \le x \le W_{\mathrm{Dp}}\right)$$

(37)

By setting $\psi_{\mathrm{S}}(N_{\mathrm{A}}) = 0$, we can get the potential distribution across the MIS contact.

$$\psi(x) = \frac{qN_{\mathrm{A}}\left(x - W_{\mathrm{Dp}}\right)^2}{2\varepsilon_{\mathrm{S}}} - \frac{q\rho_{\mathrm{piezo}}\left(x - W_{\mathrm{piezo}}\right)^2}{2\varepsilon_{\mathrm{S}}} \quad (0 \le x \le W_{\mathrm{piezo}})$$

(38)

$$\psi(x) = \frac{qN_{\mathrm{A}}\left(x - W_{\mathrm{Dp}}\right)^2}{2\varepsilon_{\mathrm{S}}} \quad \left(W_{\mathrm{piezo}} \le x \le W_{\mathrm{Dp}}\right)$$

(39)

Thus, the band bending of the semiconductor valence band can be given by

$$\psi_{\mathrm{S}} = \psi(0) = \frac{q}{2\varepsilon_{\mathrm{S}}}(N_{\mathrm{A}}W_{\mathrm{Dp}}^2 - \rho_{\mathrm{piezo}}W_{\mathrm{piezo}}^2)$$

(40)

where ε_S is the permittivity of the semiconductor, N_A is the acceptor concentration, ρ_{piezo} is the density of the piezoelectric charges, W_{piezo} is the width of the piezoelectric charges distribution region and W_{Dp} is the depletion layer width in the semiconductor.

Based on the depletion assumptions of the analytical model, most of the carriers have been removed in the depletion region. Therefore, the piezotronic charge will not be screened by free carriers. For the piezotronic MIS structure, piezoelectric charges change the energy band and building-in potential in the junction region.

In simplified analysis model, the carrier redistribution is ignored. According to typical tunneling current model, the hole and electron tunneling currents can be written as

$$J_{\mathrm{nt}} = A_{\mathrm{n}}^{*}T^{2}\exp\left(-\alpha_{\mathrm{Tn}}\varphi_{\mathrm{Tn}}^{1/2}d_{\mathrm{i}}\right)\left[\exp\left(-\frac{E_{\mathrm{c0}}-E_{\mathrm{fm}}}{kT}\right)-\exp\left(-\frac{E_{\mathrm{c0}}-E_{\mathrm{fs}}}{kT}\right)\right] = A_{\mathrm{n}}^{*}T^{2}\exp\left(-\alpha_{\mathrm{Tn}}\varphi_{\mathrm{Tn}}^{1/2}d_{\mathrm{i}}\right)\exp\left(-\frac{E_{\mathrm{g}}}{kT}\right)\exp\left(\frac{q\phi_{\mathrm{p}}+q\psi_{\mathrm{S}}}{kT}\right)\left[\exp\left(\frac{qV}{kT}\right)-1\right]$$

(41)

$$J_{\mathrm{pt}} = A_{\mathrm{p}}^{*}T^{2}\exp\left(-\alpha_{\mathrm{Tp}}\varphi_{\mathrm{Tp}}^{1/2}d_{\mathrm{i}}\right)\left[\exp\left(-\frac{E_{\mathrm{fs}}-E_{\mathrm{v0}}}{kT}\right)-\exp\left(-\frac{E_{\mathrm{fm}}-E_{\mathrm{c0}}}{kT}\right)\right] = A_{\mathrm{p}}^{*}T^{2}\exp\left(-\alpha_{\mathrm{Tp}}\varphi_{\mathrm{Tp}}^{1/2}d_{\mathrm{i}}\right)\exp\left(-\frac{q\phi_{\mathrm{p}}+q\psi_{\mathrm{S}}}{kT}\right)\left[1-\exp\left(-\frac{qV}{kT}\right)\right]$$

(42)

where A_{n}^{*} and A_{p}^{*} are effective Richardson constants for electrons and holes, α_{Tn} and α_{Tp} equal to $2\sqrt{2qm_{\mathrm{n}}^{*}}/\hbar$ and $2\sqrt{2qm_{\mathrm{p}}^{*}}/\hbar$ (m_{n}^{*} and m_{p}^{*} are the effective mass of electrons and holes, $\hbar$ is reduced Planck constant), respectively, φ_{Tn} and φ_{Tp} are effective barrier heights for electrons and holes tunneling into metal. The total current density can be given by

$$J_{\mathrm{t}} = J_{\mathrm{pt}} + J_{\mathrm{nt}}$$

(43)

It illustrates that the induced piezoelectric charges can modulate the tunneling current J_{t} by changing the surface potential ψ_{S} of semiconductor upon mechanical stimuli.

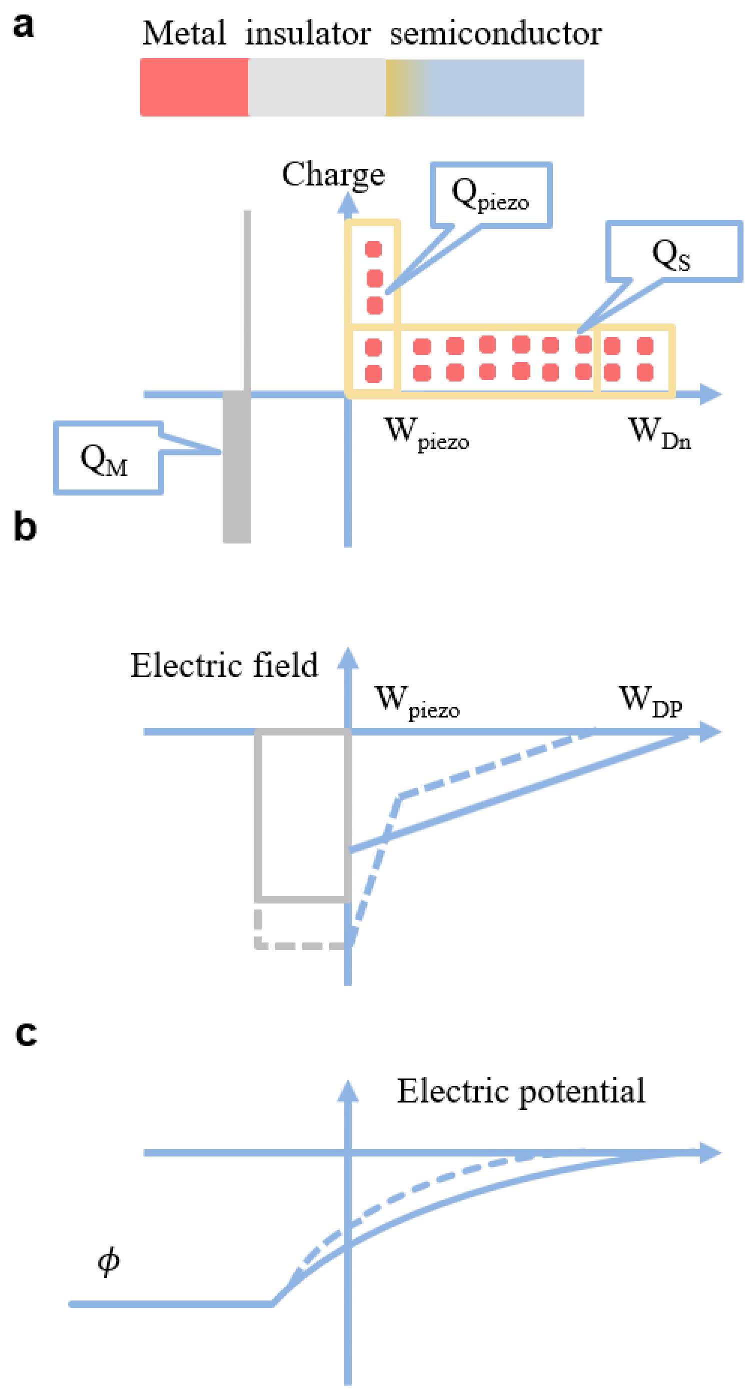

Supplementary Fig. 15 | Ideal metal-insulator-piezoelectric semiconductor contact with the presence of piezoelectric charges when applying positive voltage to metal. a, Charge distribution. **b,** Electric field. **c,** Potential distribution.

Supplementary Note 12 | Piezotronic Effect on the Barrier Width of Ag/HfO_2/n-ZnO Tunneling Junctions

Based on the band theory, the energy profile of the Ag/HfO_2/n-ZnO tunneling junction can be illustrated as Supplementary Fig. 16. A wide depletion layer (yellow area in Supplementary Fig. 16) will be formed in the n-ZnO under strain-free state. The free electrons on the conduction band need high energy to pass through the barrier with a thick width. The thin blue arrow denotes the electron transport characters. When applying a stress/strain, the strain-induced positive piezoelectric polarization charges at the interface of HfO_2/n-ZnO will bend the interface energy band downward (Supplementary Fig. 16b). In this situation, the width of the depletion layer on the n-ZnO side will become narrower, so that the electrons on the conduction band can pass through the decreased barrier with a smaller energy, as indicated by the thick blue arrow in Supplementary Fig. 16b.

In order to verify the above analysis, we use the WKB approximation to simulate the potential distribution of the Ag/HfO_2/n-ZnO tunneling junction[15], as shown in Supplementary Fig. 17. It can be found that when the fixed bias voltage is 0.5 V, as the strain increases, the potential distribution will gradually shift from the semiconductor (n-ZnO) toward the metal (Ag), resulting in the voltage drop gradually concentrates on the insulating layer (HfO_2). The voltage drop of n-ZnO decreases with the increase of strain, indicating that the effective width of the barrier on the semiconductor side is decreasing. Therefore, the strain-induced positive piezoelectric charges will decrease the effective barrier width of the tunneling junction, which can be seen directly from the simulation results. These results are consistent with the band analysis in Supplementary Fig. 16.

To further confirm that the width of the tunneling barrier can be modulated by strain and give a convincing demonstration, we investigate the *C-V* characteristics of the tunneling junction under different strains. Supplementary Fig. 18 shows the regulation mechanism of the *C-V* characteristics by the piezotronic effect. The energy profiles of Metal-Insulator-Semiconductor (MIS) tunneling junction under strain-free and strain conditions in thermal equilibrium state (left), flat-band state (middle) and weak inversion state (right) are exhibited in Supplementary Fig. 18a. The strain-induced positive piezoelectric charges will reduce the barrier height and barrier width in the state of thermal equilibrium. To achieve the same energy band state, the

required bias voltage is different[15]. For example, the relationship of the flat band voltage V_{FB} and the weak inversion voltage $V_{\min}$ under strain and strain-free follows:

$$V_{\mathrm{FB,piezo}} = V_{\mathrm{FB}} + \Delta\varphi_{\mathrm{piezo}}$$
$$V_{\mathrm{min,piezo}} = V_{\min} + \Delta\varphi_{\mathrm{piezo}}$$

(44)

where, $\Delta\varphi_{\mathrm{piezo}}$ is the barrier height change induced by piezoelectric charges. Similar analysis and conclusion can also be done for other energy band states like the strong inversion state, which is omitted here.

When the MIS barrier width changes, the ratio of the capacitance of the tunneling junction C to the capacitance of the insulating layer C_{i} will change[15], which satisfies the relationship:

$$\frac{C}{C_{\mathrm{i}}} = \frac{1}{1 + \frac{\varepsilon_{\mathrm{i}} d_{\mathrm{s}}}{\varepsilon_{\mathrm{s}} d_{\mathrm{i}}}}$$

(45)

where ε_{i} and ε_{s} are the dielectric constants of insulator and semiconductor respectively, d_{i} and d_{s} are the effective width of insulator and semiconductor respectively.

Supplementary Fig. 18b schematically shows the *C*-*V* curves under strain (red) or strain-free (black) corresponding to Supplementary Fig. 18a. The strain-induced positive piezoelectric polarization charges will enable the *C*-*V* characteristics of piezotronic tunneling junction shift to the upper right. As shown in Supplementary Fig. 18b, the barrier height change will cause a shift $\Delta\varphi_{\mathrm{piezo}}$ of the *C*-*V* curve in the horizontal direction; while the change of barrier width, which determines the value of C/C_{i} by the equation (45), will cause a shift $\Delta(C/C_{\mathrm{i}})$ of the *C*-*V* curve in the vertical direction[15]. Supplementary Fig. 18c shows the measured *C*-*V* curves of Ag/HfO_2/n-ZnO with insulator thickness of 1.8 nm under different strains. The *C*-*V* curve shifts to the upper right (red) as the tensile strain increases; while shifts to the lower left (blue) as the compressive strain increases. It can be seen from the shift in capacitance direction of the curve that the tunneling barrier width is effectively modulated by the strain.

Therefore, through the above theory, simulation and experiment, we can confirm that the strain can regulate the effective barrier width of the tunneling junction

(Ag/HfO_2/n-ZnO) through the piezotronic effect.

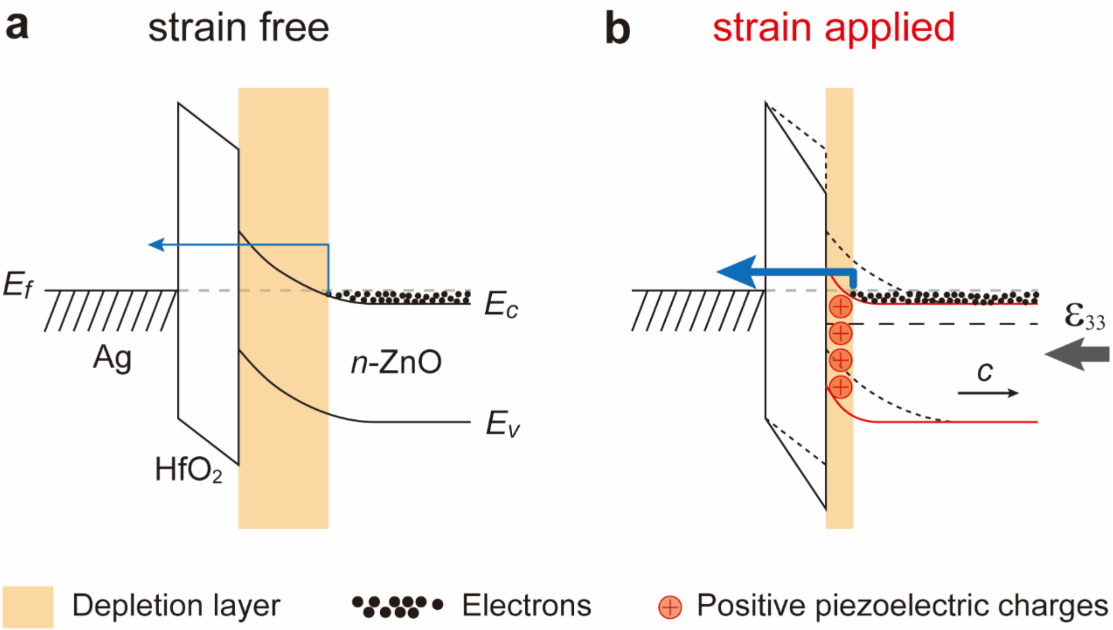

Supplementary Fig. 16 | Barrier profiles. The barrier profiles of the Ag/HfO_2/n-ZnO piezotronic tunneling junction under **(a)** strain-free and **(b)** strain. The yellow areas present the depletion layer and the blue arrows denote the transport characters.

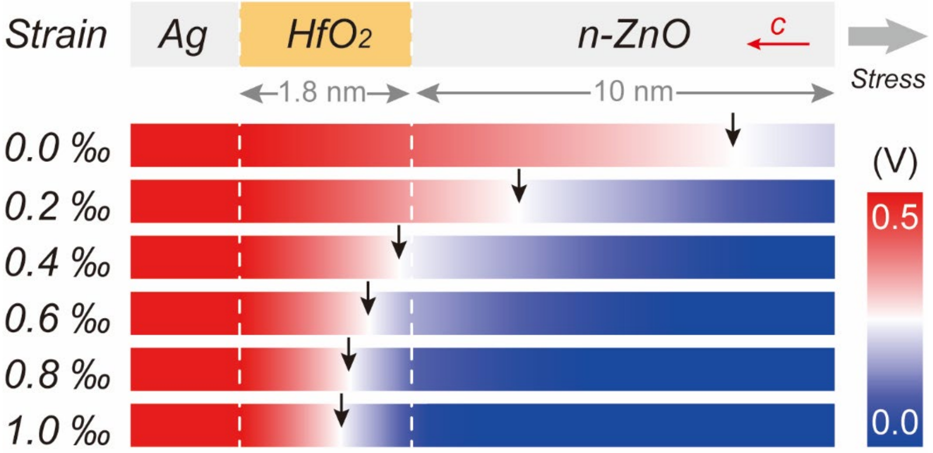

Supplementary Fig. 17 | Potential distribution of the tunneling junction. Calculated potential distribution of the Ag/HfO_2/n-ZnO under different strain ranged from 0.0 to 1.0 ‰ along *c*-axis. The potentials of Ag and n-ZnO are respectively biased to be 0 V and 0.5 V.

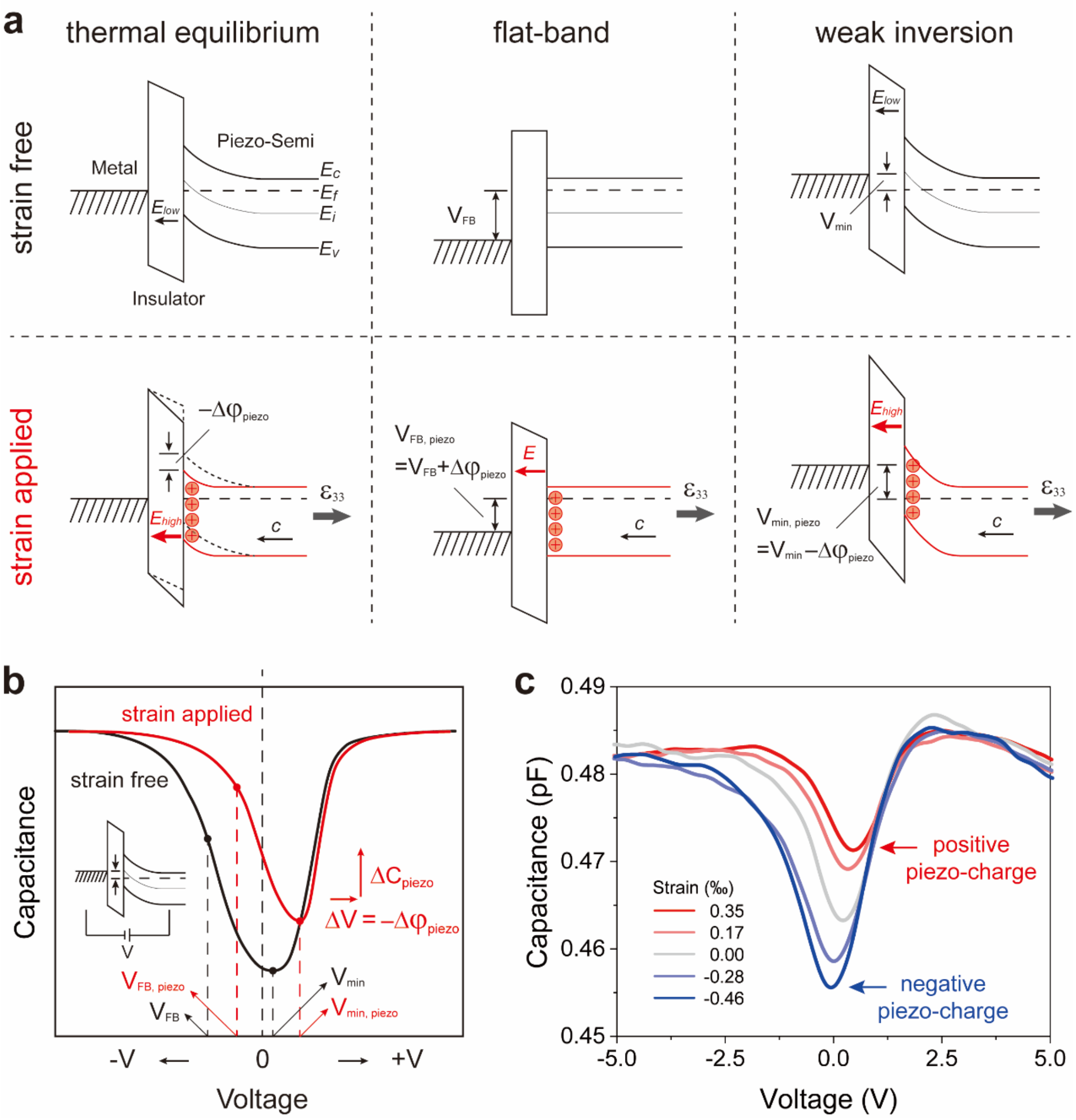

Supplementary Fig. 18 | Piezotronic modification of C-V characteristics of MIS tunneling junction. a, Energy profiles of MIS tunneling junction with strain free (black) and strain applied (red) in thermal equilibrium state (left), flat-band state (middle) and weak inversion state (right). **b,** The schematic diagram of *C*-*V* curves corresponding to the MIS tunneling junction with strain free (black) and strain applied (red). **c,** The *C*-*V* characteristics of Ag/HfO_2/n-ZnO under different strains.

Supplementary Note 13 | How to Decouple Piezotronic Modifications of Barrier Height and Barrier Width on the Current Change

For the MIS tunneling junction like piezotronic tunneling junction, the tunneling current I and the effective barrier height $(BH)_{\text{eff}}$ approximately satisfy the following relationship[15],

$$I \propto \exp[-\frac{(BH)_{\text{eff}}}{kT}] \tag{46}$$

where k is Boltzmann constant, and T is the absolute temperature.

Based on the piezotronic theory[23], the strain-induced piezotronic polarization charges at the interface will linearly change the interface barrier by the following equation,

$$\Delta(BH)_{\text{eff}} = \frac{1}{2}\Delta\varphi_{\text{piezo}} = -\frac{q^2\rho_{\text{piezo}}W_{\text{piezo}}^2}{4\varepsilon_{\text{S}}} \tag{47}$$

where q is the absolute value of the unit electronic charge, ρ_{piezo} is the density of the polarization charge (in the unit of electronic charge), W_{piezo} is the width of the piezoelectric charge distribution on the junction interface, ε_{S} is the permittivity of the piezoelectric semiconductor material.

As the strain ε_{33} is applied along the *c*-axis of ZnO nanowire, the $\Delta(BH)_{\text{eff}}$ can be also expressed as the equation[23]:

$$\Delta(BH)_{\text{eff}} = \frac{1}{2}\Delta\varphi_{\text{piezo}} = -\frac{qe_{33}\varepsilon_{33}\,W_{\text{piezo}}}{4\varepsilon_{\text{S}}} \propto \text{strain} \tag{48}$$

According to the above relation (46) and (48), the current influenced by the change of barrier height satisfies:

$$\Delta[-kT\cdot\ln(I)]_{\Delta BH} = -kT\cdot[\ln(I_{\text{strain}}/I_{\text{free}})]_{\Delta BH} \propto \text{strain} \tag{49}$$

where I_{strain} and I_{free} represent the current under strain and strain-free state, $[\ln(I_{\text{strain}}/I_{\text{free}})]_{\Delta BH}$ represents the part of the current change caused by the barrier height change.

In fact, the effective barrier width $\Delta(BW)_{\text{eff}}$ will also have an effect on the current. Thus, we can obtain a qualitative formula,

$$-ln(I_{\text{strain}}/I_{\text{free}}) = -\{[\ln(I_{\text{strain}}/I_{\text{free}})]_{\Delta BH} + [\ln(I_{\text{strain}}/I_{\text{free}})]_{\Delta BW}\}$$
$$= \alpha_{\Delta BH} \cdot \text{strain} - [\ln(I_{\text{strain}}/I_{\text{free}})]_{\Delta BW}$$

(50)

where $\alpha_{\Delta BH}$ is coefficient, and $[\ln(I_{\text{strain}}/I_{\text{free}})]_{\Delta BW}$ represents the part of the current change caused by the barrier width change.

Therefore, the part in $\ln(I_{\text{strain}}/I_{\text{free}})$ that has a linear relationship with strain is induced by the change of barrier height $\Delta(BH)_{\text{eff}}$; the other part is induced by the change of barrier width $\Delta(BW)_{\text{eff}}$.

From the results of the finite element simulation in Supplementary Fig. 17, we can find that, with the increase of the tensile strain, the electric potential distribution first appears a large shift to the metal (Ag) and gradually reach saturation when the tensile strain increases to a certain degree. It means that the reduction of the effective barrier width will gradually slow down with the continuous increasement of positive piezoelectric charges at the interface. Thus, the effect of barrier width on current mainly occurs at small tensile strain. When the tensile strain is large enough, the change of the barrier height plays a major role.

As shown in the Supplementary Fig. 19, by plotting the curve $\ln(I_{\text{strain}}/I_{\text{free}})$ as a function of strain, we can find that $\ln(I_{\text{strain}}/I_{\text{free}})$ changes approximately linearly with strain in the red region (strain >0.02%) and changes drastically and nonlinearly with strain in the blue region (strain<0.02%, Supplementary Fig. 19b). According to the previous analysis, we can determine that when the strain is large enough (red region in Supplementary Fig. 19a), the effect of the barrier height dominant the regulation; while when the strain is small (blue region in Supplementary Fig. 19a), the effect of the barrier width plays a leading role.

Additionally, as the previous discussion (Supplementary Fig. 18), we can also decouple the effects of barrier height and width by measuring the *C-V* curve of the tunneling junction under different strain. The effect of barrier height will cause the *C-V* curve to shift in the horizontal direction; while the effect of barrier width will cause the *C-V* curve to shift in the vertical direction. In this way, we can intuitively observe the influences of the barrier height change and the barrier width change by piezotronic effect on the MIS tunneling junction.

Through the above analysis, we have respectively given the methods to decouple the effects of barrier height and width based on the *I*-strain curve (Fig. 3 in the manuscript

and Supplementary Fig. 19) and the *C*-*V* curve (Supplementary Fig. 18).

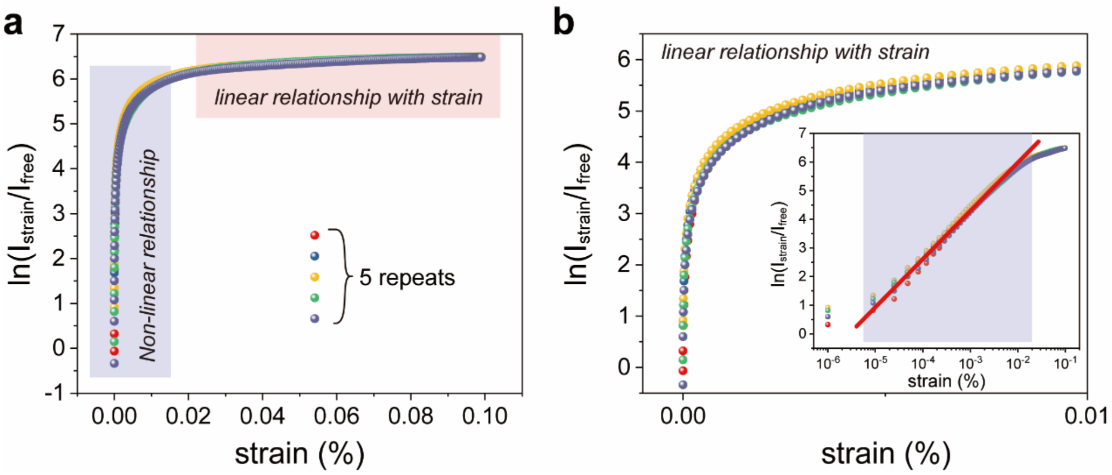

Supplementary Fig. 19 | $\ln(I_{strain}/I_{free})$ as a function of strain. (a) $\ln(I_{strain}/I_{free})$ -strain curves derived from *I-strain* curves. (**b**) Enlarged $\ln(I_{strain}/I_{free})$ of device under a small strain range from 0 to 0.1‰. Inset: $\ln(I_{strain}/I_{free})$ as a function of strain presented in semilog form.

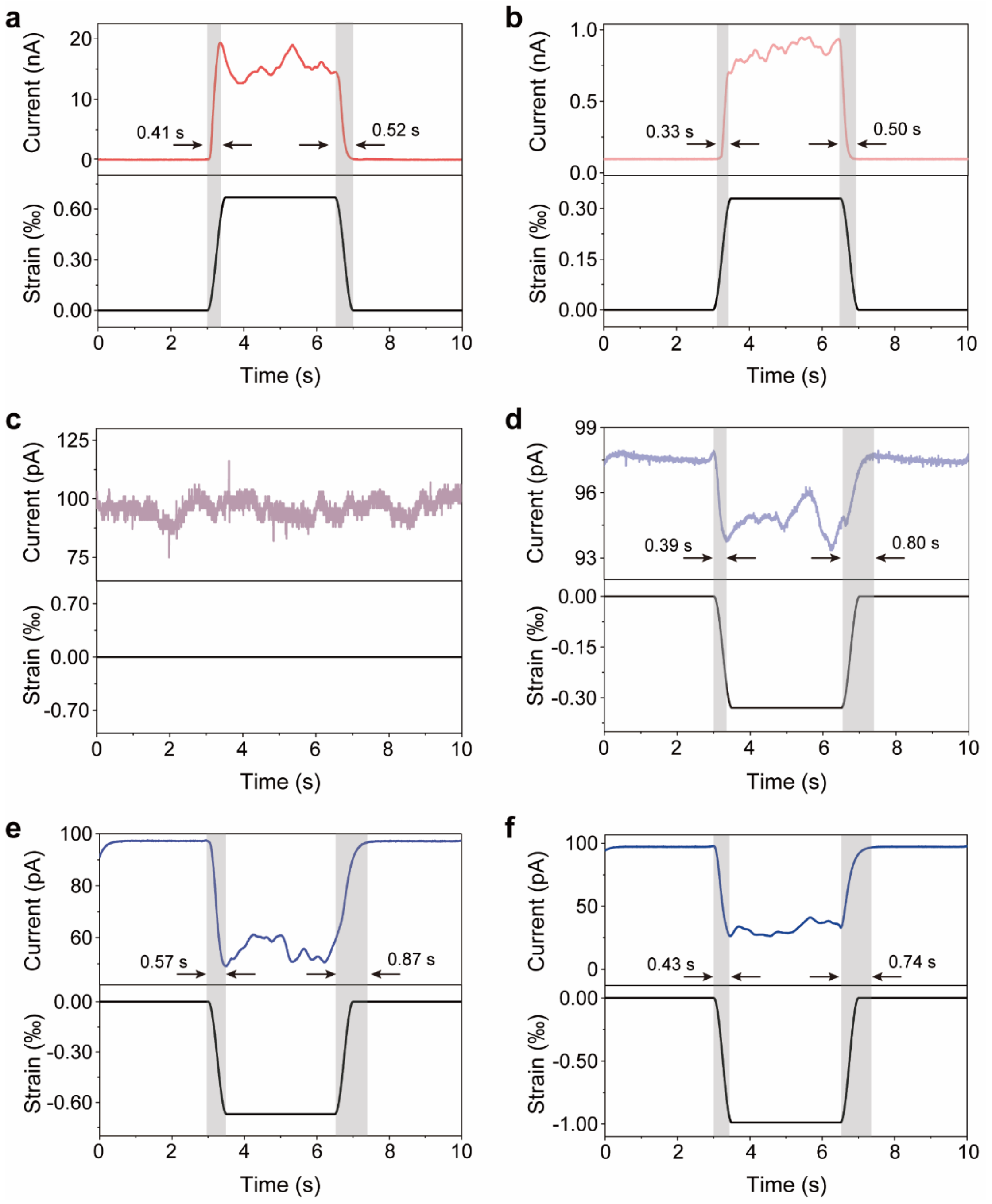

Supplementary Fig. 20 | Current response-recovery time curve for Ag/HfO_2/n-ZnO PTSS. a, b and **c** represent the detailed response current under the tensile strains of 0.067%, 0.033% and 0.00%, respectively. **d, e** and **f** represent the detailed response current under the compressive strains of 0.067%, 0.033% and 0.00%, respectively.

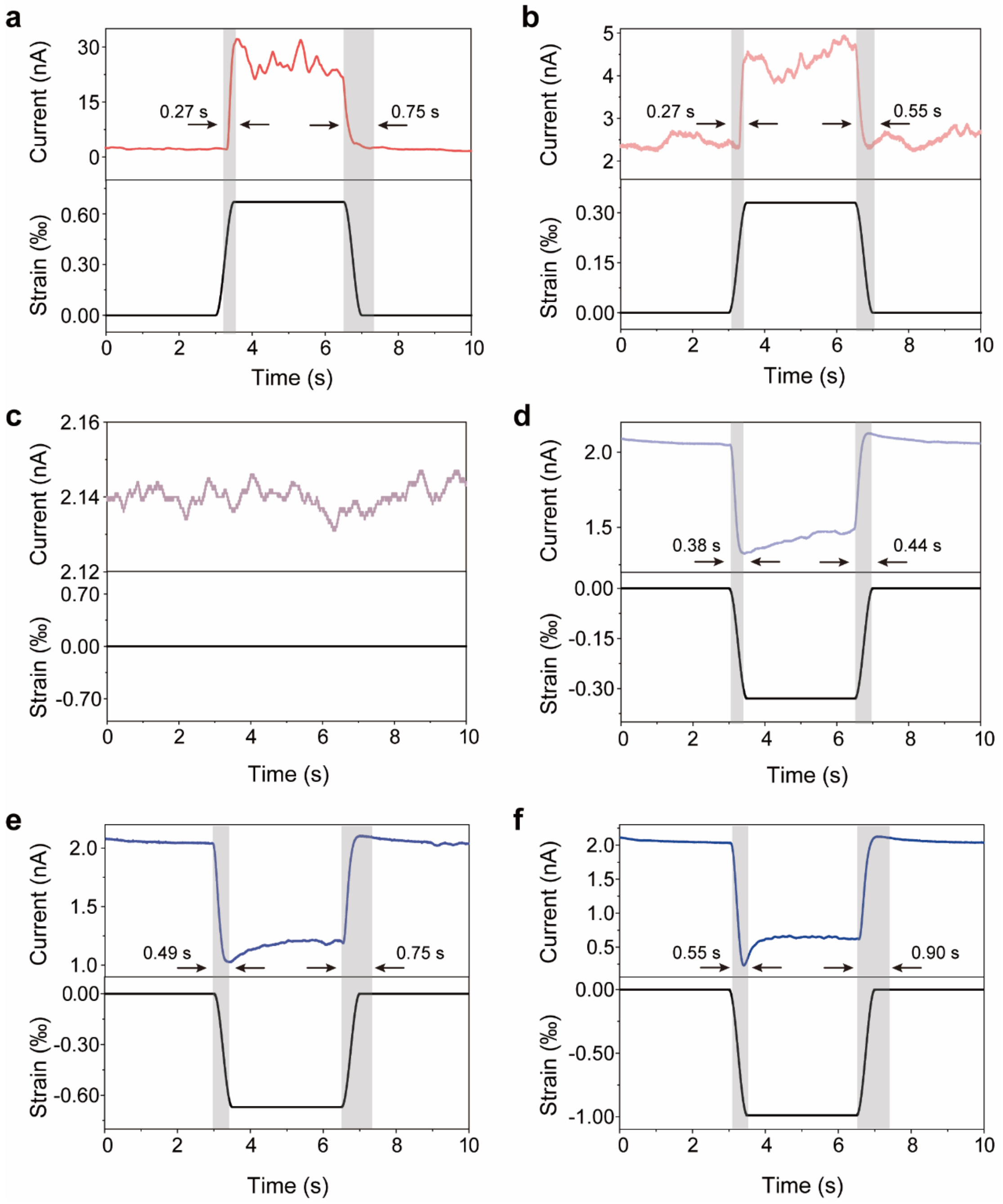

Supplementary Fig. 21 | Current response-recovery time curve for Ag/n-ZnO SSS. a, b and **c** represent the detailed response current under the tensile strains of 0.067%, 0.033% and 0.00%, respectively. **d, e** and **f** represent the detailed response current under the compressive strains of 0.067%, 0.033% and 0.00%, respectively.

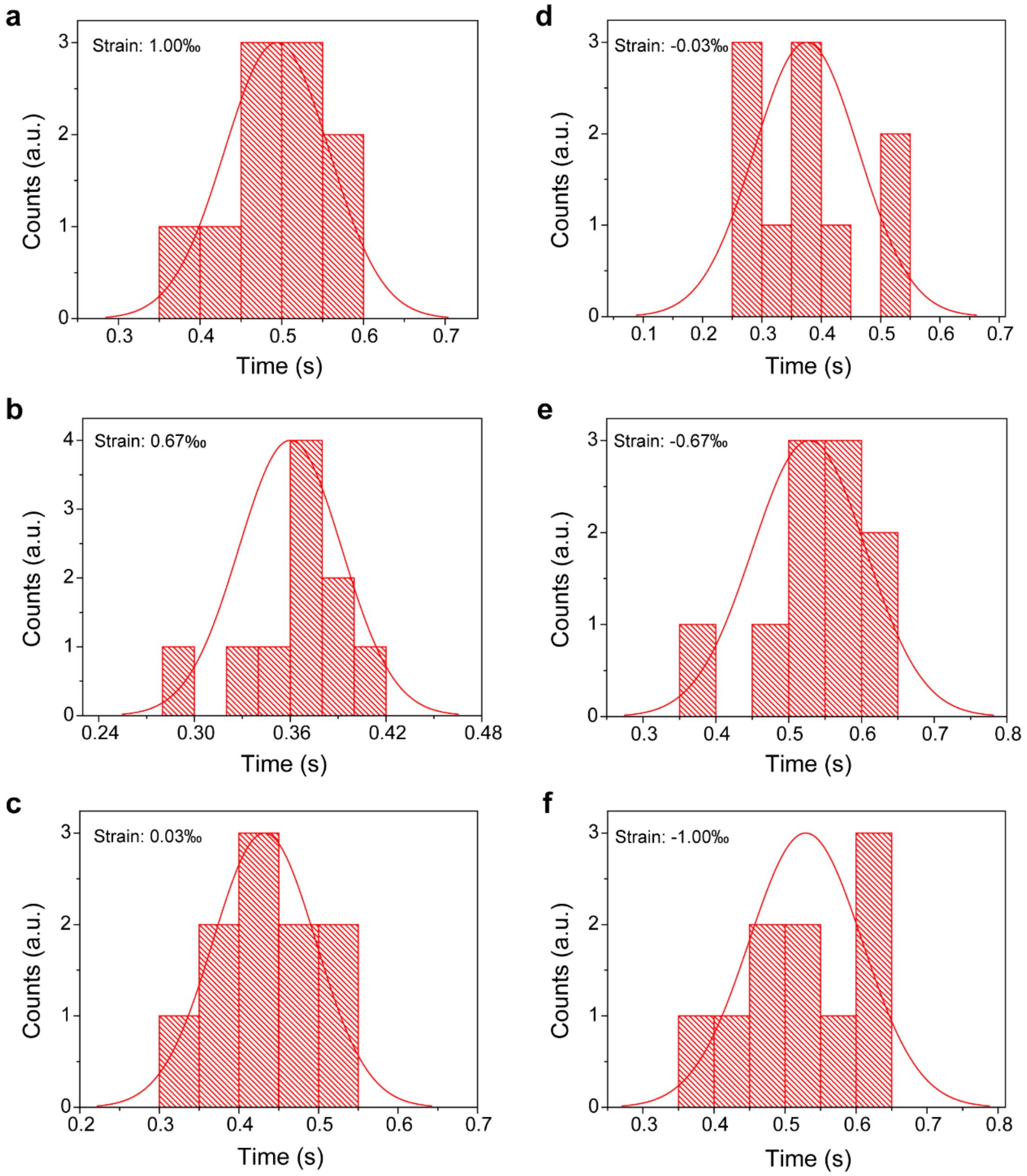

Supplementary Fig. 22 | The statistical distribution of the response time for Ag/HfO_2/n-ZnO PTSS. a, b and **c** represent the response time statistics under the tensile strains of 0.10%, 0.067% and 0.033%, respectively. **d, e** and **f** represent the response time statistics under the compressive strains of 0.033%, 0.067% and 0.10%, respectively.

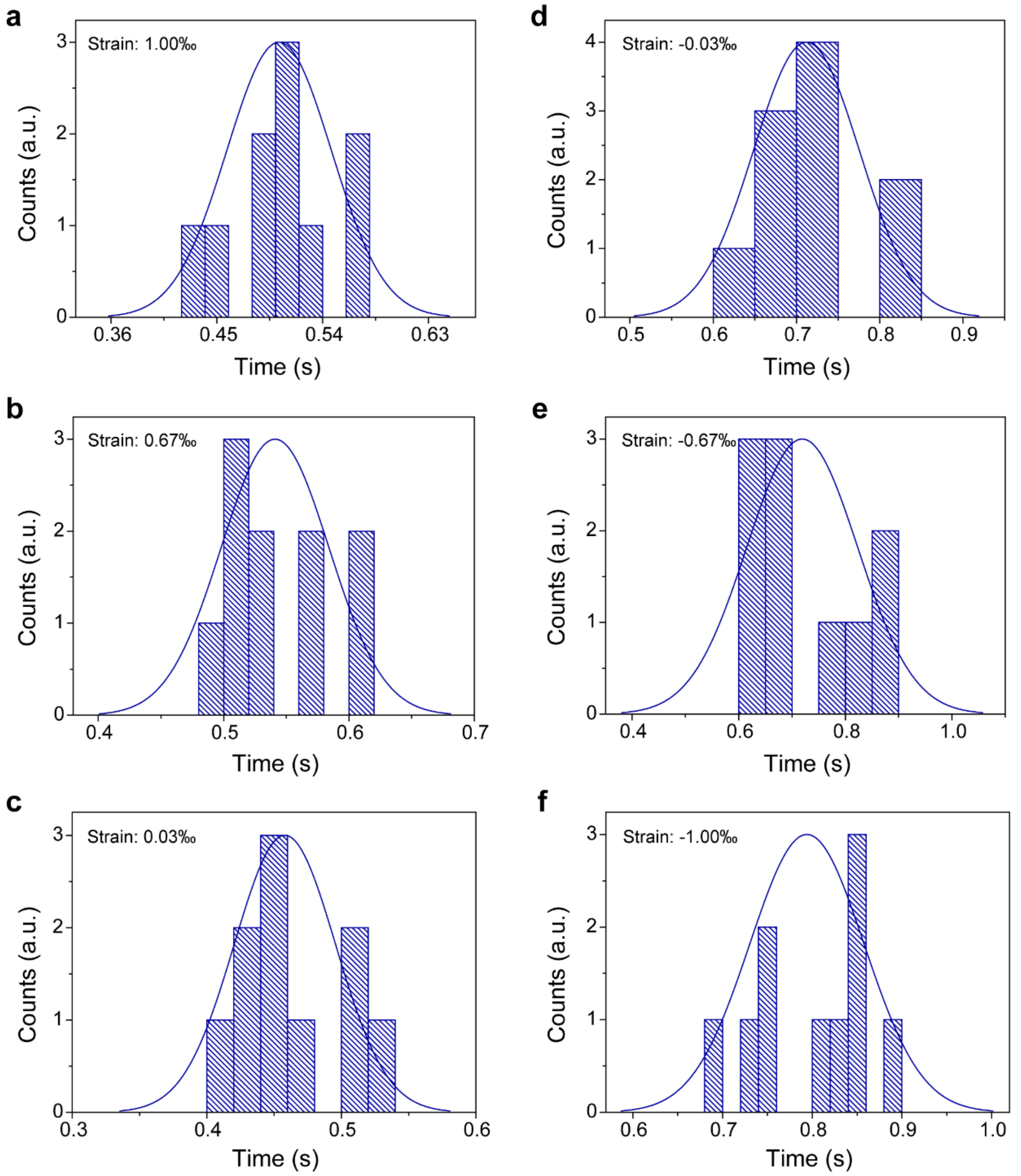

Supplementary Fig. 23 | The statistical distribution of the recovery time for Ag/HfO_2/n-ZnO PTSS. a, b and **c** represent the recovery time statistics under the tensile strains of 0.10%, 0.067% and 0.033%, respectively. **d, e** and **f** represent the recovery time statistics under the compressive strains of 0.033%, 0.067% and 0.10%, respectively.

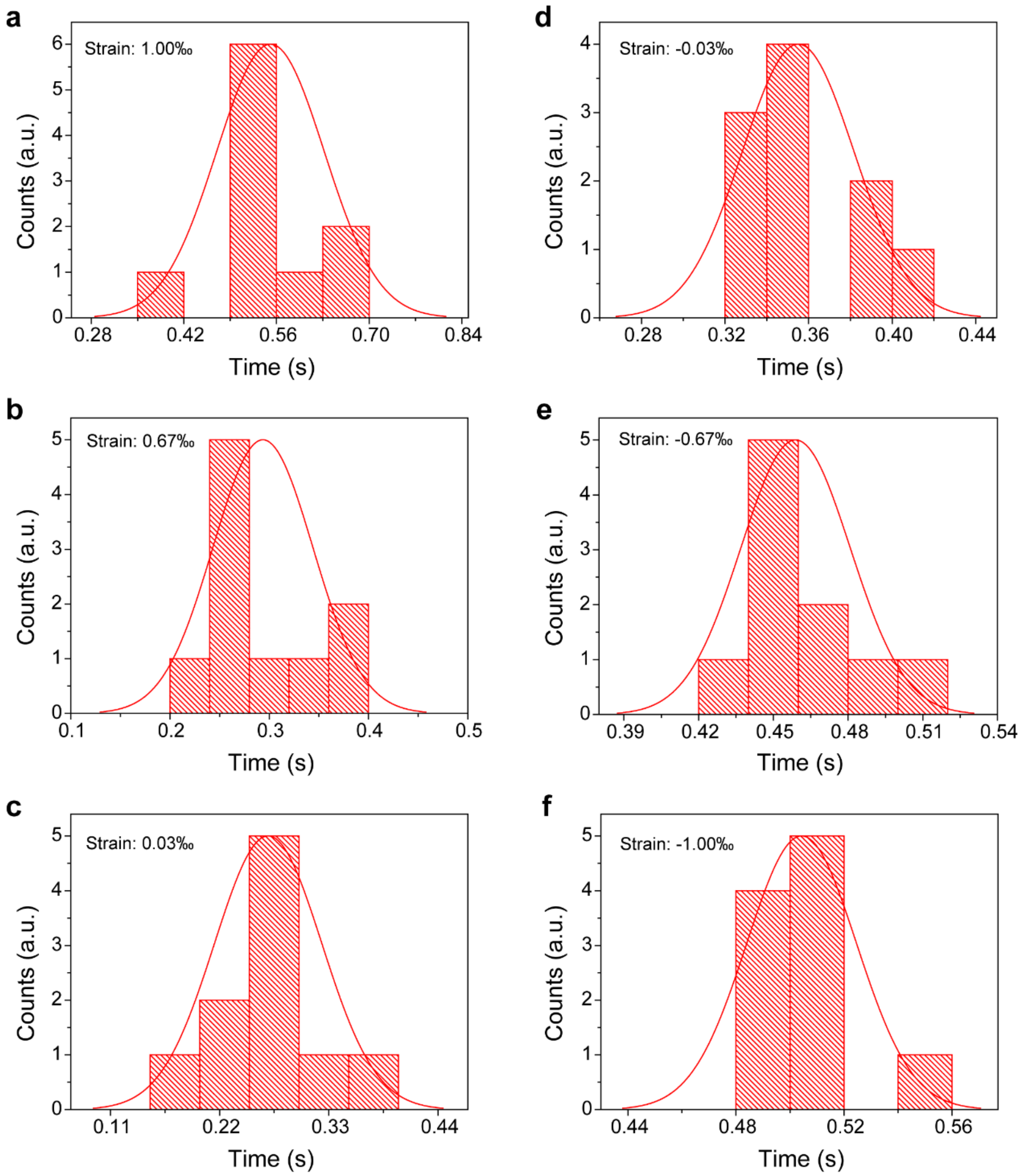

Supplementary Fig. 24 | The statistical distribution of the response time for Ag/n-ZnO SSS. a, b and **c** represent the response time statistics under the tensile strains of 0.10%, 0.067% and 0.033%, respectively. **d, e** and **f** represent the response time statistics under the compressive strains of 0.033%, 0.067% and 0.10%, respectively.

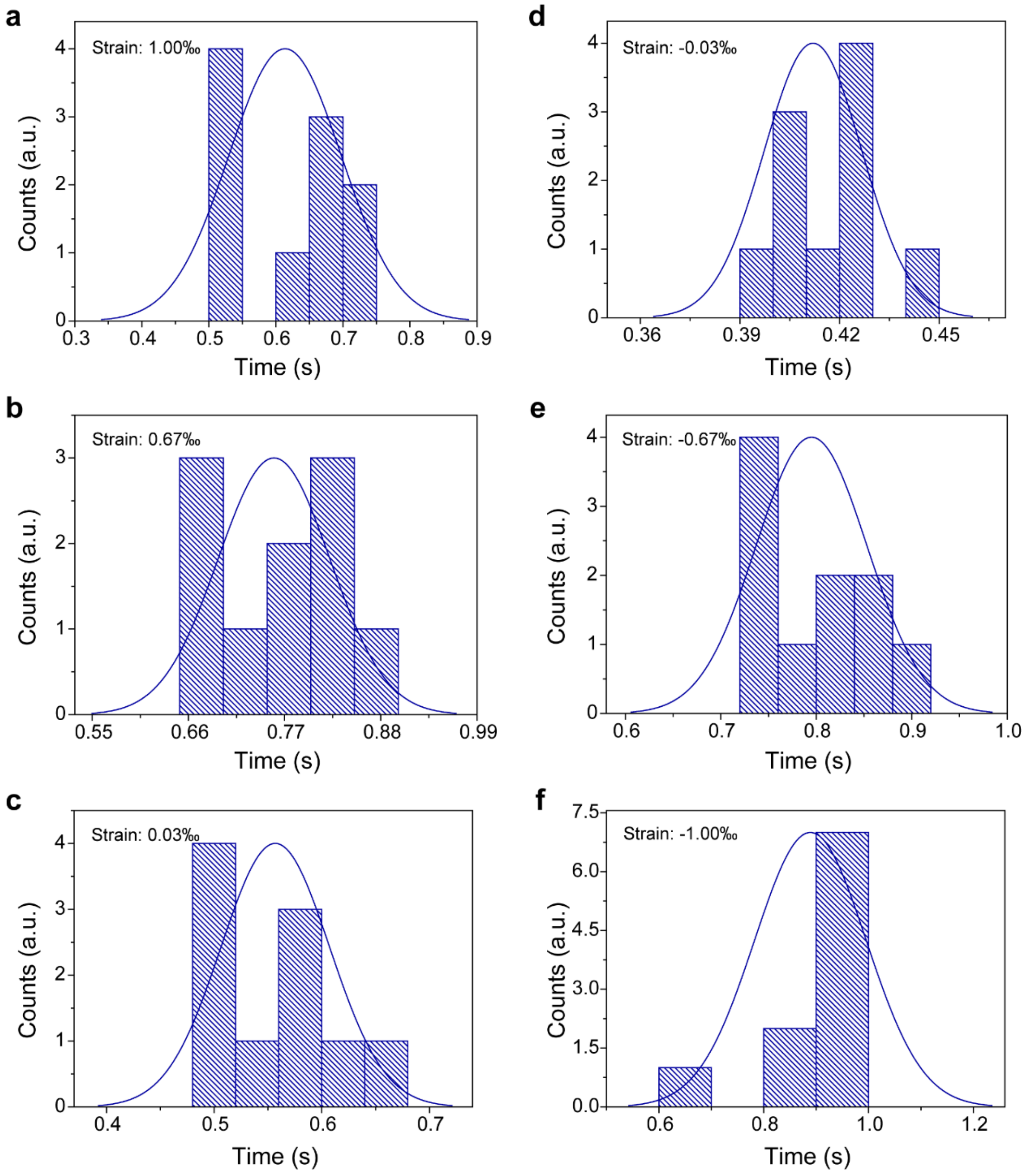

Supplementary Fig. 25 | The statistical distribution of the recovery time for $Ag/HfO_2/n\text{-}ZnO$ PTSS. a, b and **c** represent the recovery time statistics under the tensile strains of 0.10%, 0.067% and 0.033%, respectively. **d, e** and **f** represent the recovery time statistics under the compressive strains of 0.033%, 0.067% and 0.10%, respectively.

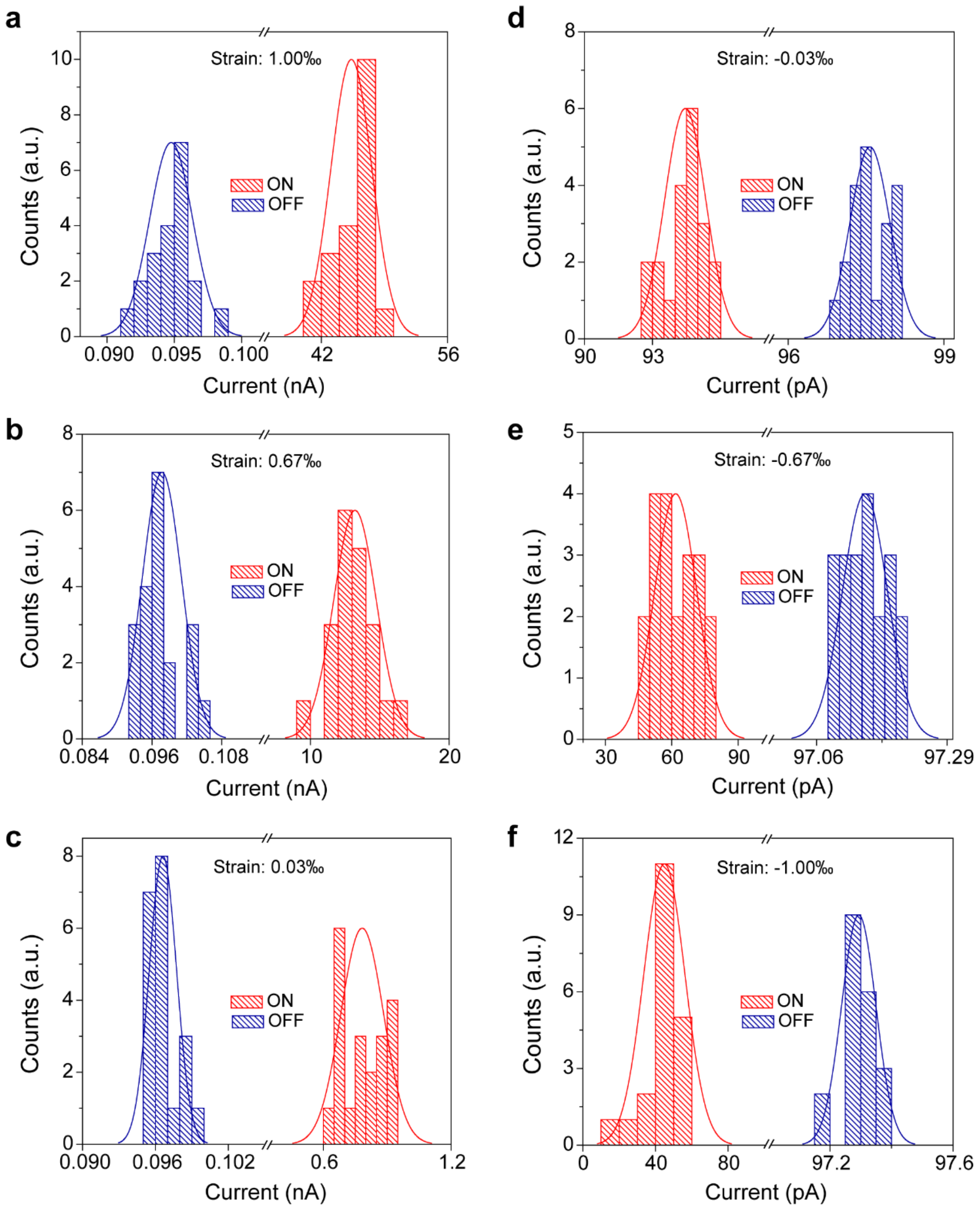

Supplementary Fig. 26 | The statistical distribution of the on-state and off-state current for Ag/HfO_2/n-ZnO PTSS. a, b and **c** represent the on-off current statistics under the tensile strains of 0.10%, 0.067% and 0.033%, respectively. **d, e** and **f** represent the on-off current statistics under the compressive strains of 0.033%, 0.067% and 0.10%, respectively.

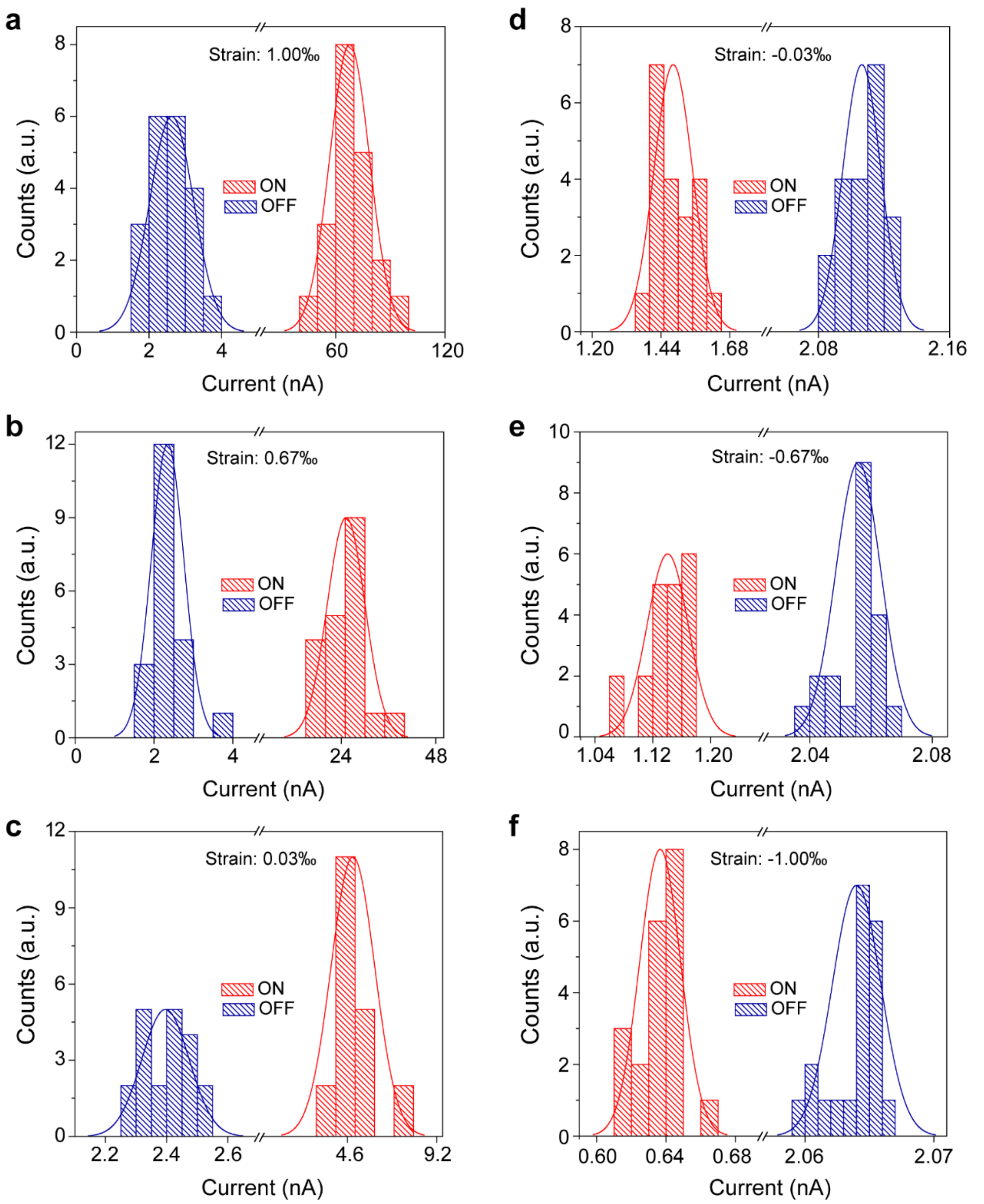

Supplementary Fig. 27 | The statistical distribution of the on-state and off-state current for Ag/n-ZnO SSS. a, b and **c** represent the on-off current statistics under the tensile strains of 0.10%, 0.067% and 0.033%, respectively. **d, e** and **f** represent the on-off current statistics under the compressive strains of 0.033%, 0.067% and 0.10%, respectively.

Supplementary Note 14 | Ag/HfO_2/n-ZnO Tunneling Junctions with Various Insulator Thicknesses

In order to characterize the nature of the Ag/HfO_2/n-ZnO tunneling junctions with various insulator thicknesses, we have measured the barrier height change induced by insulator thickness, and the on/off ratio of the tunneling junction under strain-free and strain.

(1) *C*-*V* of Ag/HfO_2/n-ZnO Tunneling Junctions with Various Insulator Thicknesses

Supplementary Fig. 28 schematically shows the influence of insulator thickness on the *C*-*V* characteristics. The energy profiles of Metal-Insulator-Semiconductor (MIS) tunneling junction with different insulator thickness in thermal equilibrium state (left), flat-band state (middle) and weak inversion state (right) are exhibited in Supplementary Fig. 28a. Different insulating layer thickness will change the potential distribution of the tunneling junction, and thus make the energy profile of the tunneling junction different.

In the thermal equilibrium state (Supplementary Fig. 28a, left), a thick insulating layer will withstand more voltage drop; meanwhile, the semiconductor will bear the smaller voltage drop[15, 35]. As in a weak inversion state (Supplementary Fig. 28a, right), the tunneling junction with a thick insulating layer will need a larger bias voltage $V_{\mathrm{min,thick}}$ to achieve the same semiconductor energy band bending as the one ($V_{\mathrm{min,thin}}$) of tunneling junction with thin insulating layer. These two required bias voltages follow:

$$V_{\mathrm{min,thick}} = V_{\mathrm{min,thin}} + \Delta V_{\mathrm{insulator}} \tag{51}$$

where $\Delta V_{\mathrm{insulator}}$ represents the voltage drop of the insulating layer due to the change in the thickness of the insulating layer. Therefore, as shown in Supplementary Fig. 28b, the change in the thickness of the insulating layer will cause the bottom (corresponding to the weak inversion state) of the *C*-*V* curve to shift in the horizontal direction. According to the MIS energy band theory[35], the barrier height change ($\Delta BH_{\mathrm{weak\ inversion}}$) caused by the insulator thickness of the tunneling junction in the weak inversion state satisfies:

$$\Delta BH_{\text{weak inversion}} \approx -\frac{1}{2}\left(V_{\text{min,thin}} - V_{\text{min,thick}}\right) = -\frac{1}{2}\Delta V_{\text{insulator}}$$

(52)

However, it should be noted that tunneling junctions with different insulating layer thicknesses require the same bias voltage V_{BF} to achieve a flat band state (Supplementary Fig. 28a, middle). The points in the *C*-*V* curves corresponding to the flat belt state should have the same bias voltage as shown in Supplementary Fig. 20b. In this condition, the barrier height change ($\Delta BH_{\text{flat-band}}$) caused by the insulator thickness of the tunneling junction in the weak inversion state satisfies:

$$\Delta BH_{\text{flat-band}} = V_{\text{BF}} - V_{\text{BF}} = 0$$

(53)

Furthermore, the change in the thickness of the insulating layer will change the ratio of the capacitance of the tunnel junction C to the capacitance of the insulating layer C_{i}, which satisfies the following equation (12), thereby causing the *C*-*V* curve to shift up and down as illustrated in Supplementary Fig. 28b.

$$\frac{C}{C_{\text{i}}} = \frac{1}{1 + \frac{\varepsilon_{\text{i}} d_{\text{s}}}{\varepsilon_{\text{s}} d_{\text{i}}}}$$

(54)

where ε_{i} and ε_{s} are the dielectric constants of insulator and semiconductor respectively, d_{i} and d_{s} are the effective width of insulator and semiconductor respectively.

Supplementary Fig. 29 exhibits the measured *C*-*V* curves and the derived barrier height of Ag/HfO_2/n-ZnO tunneling junctions with different insulator thickness (0 nm, 0.4 nm, 1.1 nm, 1.8 nm, 2.5 nm, 3.6 nm and 7.3 nm). It can be found in Supplementary Fig. 29a that as the thickness of the insulating layer increases, the *C*-*V* characteristics gradually shift to the upper right. This trend is consistent with the above theoretical analysis, and also consistent with the typical ideal *C*-*V* curves for silicon-based MIS tunneling junctions[36], which in turn indirectly indicates the good interface quality of Ag/HfO_2/n-ZnO tunneling junction. Basing on the equations (52) and (53), we can derive the barrier height change of the tunneling junction with different HfO_2 thickness, as shown in Supplementary Fig. 29b. It can be seen that the barrier height of MIS tunneling junction gradually decreases as the thickness increases in the weak inversion condition; while remains unchanged in the flat-band

condition. The absolute value (2.26 eV) of the barrier height (the right axis of Supplementary Fig. 29b) is calculated by the electron affinities of Ag (~4.26 eV[37]) and HfO_2 (~2.0 eV[38]). Therefore, the relationship between the barrier height and the thickness of insulating layer is related to the state of the junction.

(2) On/Off Ratio of Ag/HfO_2/n-ZnO Tunneling Junctions with Various Insulator Thicknesses

To explore the influence of insulator thickness on the performance of piezotronic tunneling devices, we have fabricated the Ag/HfO_2/n-ZnO devices with HfO_2 thicknesses of 0 nm, 0.4 nm, 1.1 nm, 1.8 nm, 2.5 nm, 3.6 nm and 7.3 nm, respectively, and studied their piezotronic modifications of electrical transport of 140 devices (20 devices for each thickness) under a tensile strain of 0.1%. Supplementary Fig. 30 shows the statistical distribution of the current on/off ratio of these devices. It can be found that the devices with 1.8 nm HfO_2 exhibit the highest current on-off ratio.

When the thickness of the insulating layer is larger than 1.8 nm, the regulation of the tunneling junction by the piezoelectric polarization charge will be limited. The strain-induced positive piezoelectric bound charges at the HfO_2/ZnO interface can attract electrons to move toward the semiconductor surface and lower the barrier's height and width via piezotronic effect. This profile modification of energy-band redistributes the potential drops across the piezotronic tunneling junction and improves the electric-field inside the insulator layer, which can be seen from the energy band bending of the insulating layer in the weak inversion condition. As the insulator thickness increased, the change of electric-field in the insulating layer will become smaller than the one with thinner insulator thickness. Thus, a thicker insulating layer will have negative effect on the performance of piezotronic tunneling junction.

When the thickness of the insulating layer is smaller than 1.8 nm, the leakage current will become large. Hence the tunneling junction will degenerate into a Schottky junction, and the current on/off ratio will be relatively small. In addition, the tunneling junction may be easily damaged if the insulating layer is too small, since the devices need to be deformed repeatedly under dynamic stress.

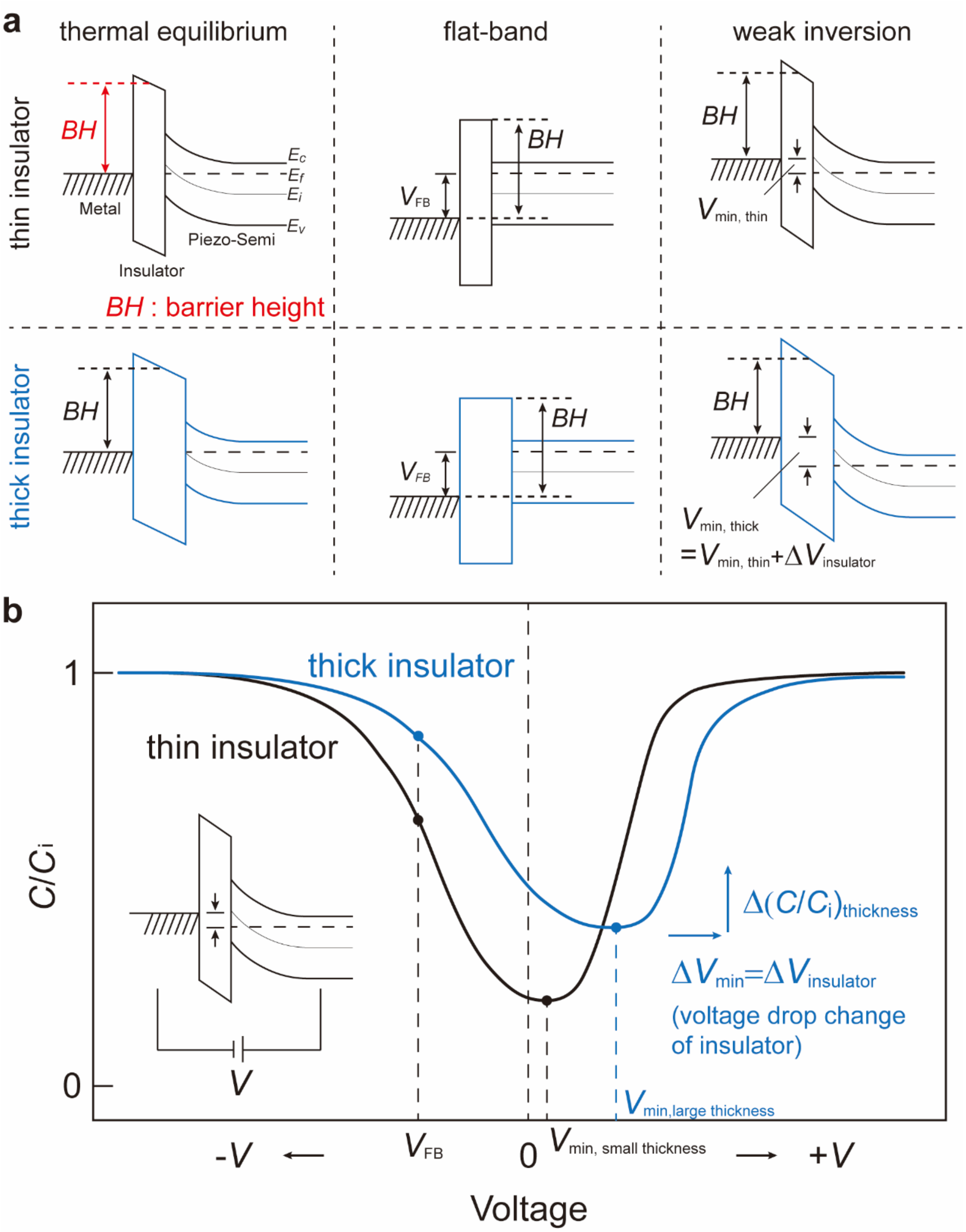

Supplementary Fig. 28 | *C*-*V* characteristics of MIS tunneling junctions with various insulator thicknesses. a, The energy profiles of MIS tunneling junction with a thin insulating layer (black) and a thick insulating layer (blue) in thermal equilibrium state (left), flat-band state (middle) and weak inversion state (right). **b,** The schematic diagram of *C*-*V* curves corresponding to the MIS tunneling junctions with a thin insulator (black) and a thick insulator (blue).

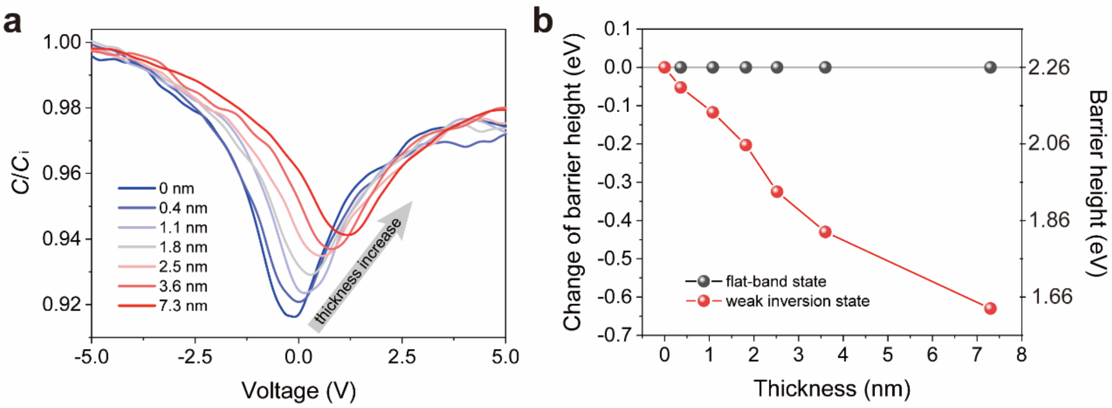

Supplementary Fig. 29 | Measured *C*-*V* characteristics of Ag/HfO_2/n-ZnO tunneling junctions for various insulator thicknesses. a, The C/C_i of the tunneling junction with various insulator thicknesses as a function of bias voltage. C and C_i represent the capacitances of the tunneling junction as a whole and the insulating layer, respectively. **b,** The change of barrier height in flat-band condition and weak inversion condition derived from figure **a**. The absolute value of the barrier height (right axis) is calculated by the electron affinities of Ag and HfO_2.

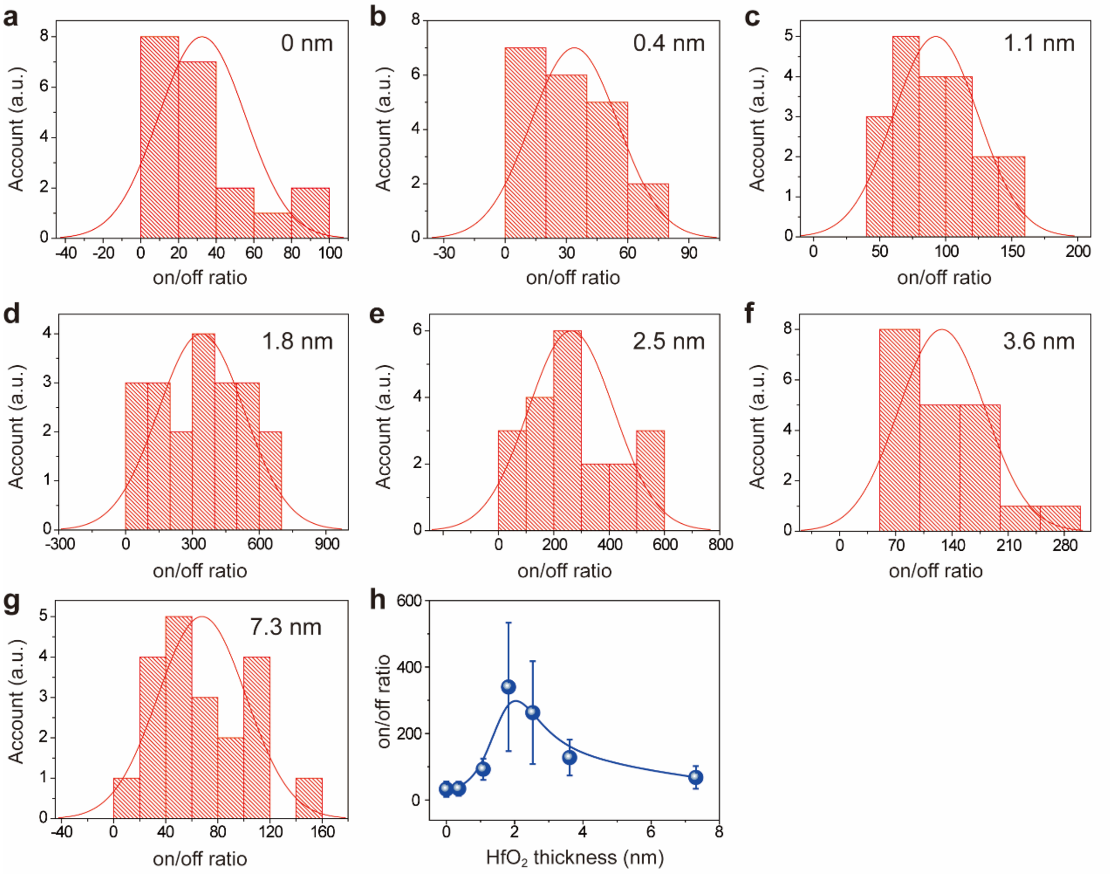

Supplementary Fig. 30 | The on/off ratio of Ag/HfO_2/n-ZnO tunneling junctions with various insulator thicknesses. a-g, The statistical distribution of current on/off ratios of the Ag/HfO_2/ZnO devices with 0 nm, 0.4 nm, 1.1 nm, 1.8 nm, 2.5 nm, 3.6 nm and 7.3 nm thick HfO_2 (20 devices for each thickness). **h,** The on/off ratio as a function of HfO_2 thickness. Error bars denote standard deviations of the mean.

Supplementary Table 1 | Response Time for Some Strain Sensing Works.

The response times of piezotronic devices are shown in Supplementary Table 1 and Supplementary Fig. 31. Since the application of force takes time, and the time of applying force of linear motor equipment is about 500 ms, the response times of our sensor devices are longer than that of ordinary piezotronic devices.

Materials	Morphology	Device Type	Work Type	Response	Ref.
Ag/HfO_2/n-ZnO	NW, single	Device	Piezotronic	250-650 ms	This work
ZnO	NW, single	Device	Piezotronic	250-650 ms	This work
ZnO	NW, single	Device	Piezotronic	10 ms	[10]
ZnO	NW, cluster	Device	Piezotronic	150 ms	[20]
GaN/Al_2O_3/Pt	Film	Based on AFM	Piezotronic	<4.38 ms	[39]
ZnO	Nanobelts	Device	Piezotronic	600 ms	[46]
ZnO	Nanobelts	Device	Piezotronic	120 ms	[47]
ZnO	NW, single	Based on AFM	Piezotronic	10 ms	[48]
GaN	NW, single	Based on AFM	Piezotronic	<5 ms	[51]
ZnO	NW, array	Device	Piezotronic	90 ms	[55]
ZnO	Twin Nanoplatelet	Device	Piezotronic	<5 ms	[56]
Ti_3C_2	Film	Device	Piezoresistive	<30 ms	[62]
Si/Ge and Polyimide		Device	Piezoresistive	<100 ms	[69]
Carbon	Nanotube film	Device	Piezoresistive	14 ms	[72]
Carbon	Nanotube Foam	Device	Piezoresistive	<100 ms	[73]
Carbon Black		Device	Piezoresistive	<20 ms	[74]

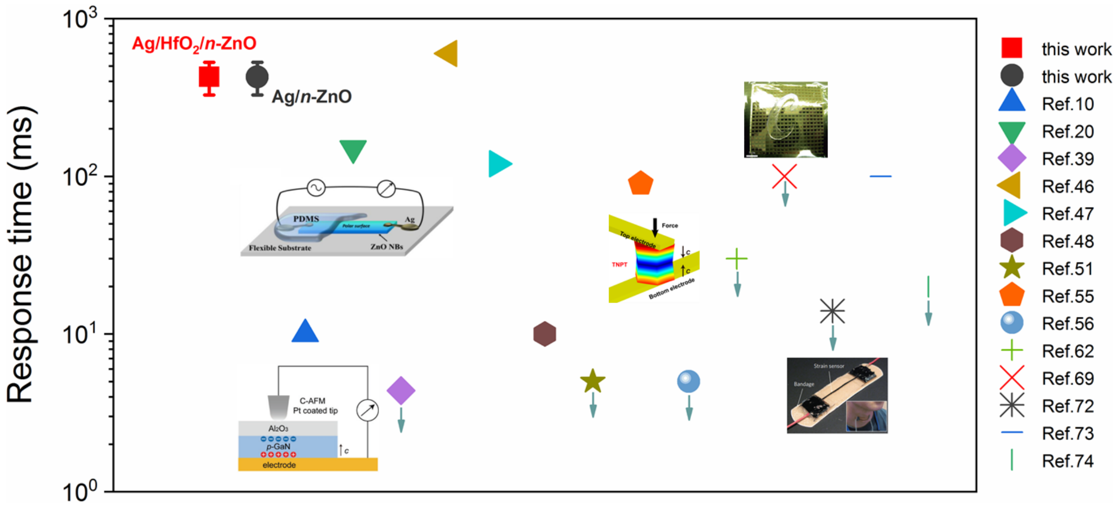

Supplementary Fig. 31 | The statistical distribution of the response time of this work (including Ag/HfO2/n-ZnO PTSS and Ag/n-ZnO SSS) and some other sensors. The downward arrow represents the actual data value less than the point, the purple represents the piezoelectric mechanism, and the yellow represents the piezoresistive mechanism.

Supplementary Table 2 | Comparison of Strain Sensing Works.

The summarized gauge factor and response of PTSS, SSS, some existing ZnO nanowire or nanobelt based sensors, and other material-based sensor shows that the PTSS based on the structure of Ag/HfO_2/n-ZnO shows obvious advantages among other sensors (including SSS), demonstrating its great potential applications in strain sensing. NW: Nanowire; Single: The device is based on a single nanomaterial. Array: The device is based on a nanomaterial array. Here, pressure (Pa), strain (%) and force (N) are used as parameters of devices' work range.

**Materials**	**Morphology**	**Type**	**Gauge factor**	**Work Range**	**Ref.**
Ag/HfO_2/n-ZnO	NW, single	Piezotronic	~4.8×10^5	0.00%~0.10%	This work
ZnO	NW, single	Piezotronic	~2.7×10^4	0.00%~0.10%	This work
GaN/Al_2O_3/Pt	Film	Piezotronic	<2.6×10^8	0.03-0.53‰	[39]
ZnO	NW, single	Piezotronic	~1250	-0.8%~1.2%	[10]
ZnO	NW, single	Piezotronic	~435	0.48%~0.96%	[14]
ZnO	NW, single	Piezotronic	~129.3	0%~0.8%	[40]
ZnO	MW, single	Piezotronic	~1010	1.14%	[41]
ZnO	NW, single	Piezoresistive	~33.8	1%	[42]
ZnO	Nanobelts	Piezotronic	~4.29	1%~5%	[43]
ZnO		Piezoresistive	~29.2	1%~5%	[44]
ZnO	NW, single	Piezoresistive	~19	0.58%~1.75%	[45]
ZnO	Nanobelts	Piezotronic	~1000	0%~0.2%	[46]
ZnO	Nanobelts	Piezotronic	~135	0.4%	[47]
ZnO	NW, single	Piezotronic	~175	0%~5%	[48]
ZnO	NW, single	Piezotronic	~1832	0%~1.31%	[11]
ZnO	NW, single	Piezotronic	~10625	0%~0.12%	[49]

GaN	NW, single	Piezotronic	~1126	0.9~1.3 μN	[50]
GaN	NW, single	Piezotronic		<300 nN	[51]
CdSe	NW, single	Piezotronic	~1590	420~700 nN	[52]
ZnO	NW, cluster	Piezotronic	~784	0~80.33 MPa	[53]
ZnO	NW, cluster	Piezotronic	$\sim 2\times10^6$	0~40 kPa	[20]
ZnO	Nanoplatelet	Piezotronic	$\sim 1.5 \times10^7$	0.02~3.64 MPa	[24]
ZnO	NW, array	Piezotronic	~1813	0%~0.8%	[54]
ZnO	NW, array	Piezotronic	~1803	-0.15%~0%	[55]
ZnO	Twin Nanoplatelet	Piezotronic	$2.9\sim9.4 \times10^9$	24~153 kPa	[56]
ZnO	Bulk	Piezotronic	150~800<br>0.7~1.4 kV	50~250 MPa	[57]
$ZnSnO_3$	NW, single	Piezotronic	~3740	0.08%~0.32%	[58]
InAs	NW, single	Piezotronic/ Piezoresistive	2820	0%~2.71%	[59]
ZnO	NW	Piezoresistive	7.64	3.5%~6.2%	[60]
MoS_2	Thin layer	Piezoresistive	56.5 ~72.5	0~100 kPa<br>-1.98%~1.98%	[61]
Ti_3C_2	Film	Piezoresistive	45.9 ~180.1	0~13 kPa	[62]
SiC	NW	Piezoresistive	~6.9	0%~9.35%	[63]
3C-SiC/Si	Film	Piezoresistive	$\sim 5.8\times10^4$	0.01%~0.07%	[64]
Si	Whisker	Piezoresistive	~225	0%~2%	[65]
p-Si	NW	Piezoresistive	~130	0%~3%	[66]
Si (depleted)	NW	Piezoresistive	~3000		[67]
Ge	NW	Piezoresistive	~5000	0%~2.1%	[68]
Si/Ge and Polyimide		Piezoresistive		0~15 kPa	[69]

Au	NW	Piezoresistive	7.38	0~3 kPa	[70]
Carbon	Nanotube	Piezoresistive	600<br>~1000	0%~0.6%	[71]
Carbon	Nanotube<br>film	Piezoresistive	0.06<br>0.82	0%~40%<br>60%~200%	[72]
Carbon	Nanotube<br>Foam	Piezoresistive	0.49<br>~2.63	1~100(+) kPa	[73]
Carbon Black		Piezoresistive	2.2	91 Pa~64 kPa<br>0.2%~60%	[74]
50wt% Carbon Black<br>+ 50wt%TPE		Piezoresistive	20	0%~80%	[75]
Graphene		Piezoresistive	2~4	0%~30%	[76]
Graphene	Film	Piezoresistive	~300	0%~0.3%	[77]
Graphene	Film	Piezoresistive	15	0%~1.7%	[78]
Graphene	Ribbon	Piezoresistive	~1.9	0%~1%	[67]
Graphene-Nanocellulose		Piezoresistive	7.1<br>~2427		[79]
Graphite	Nanoplatelet	Piezoresistive	~6.1	0%~1%	[80]
SWCNT/Graphite<br>Nanoplatelet		Piezoresistive	~5.44		[81]
PEDOT: PSS–SWCNT		Piezoresistive	0.13<br>~0.26		[82]
PDMS	Film	Piezoresistive	550<br>~5500	0.2~20 kPa	[83]
Polyurethane Acrylate		Piezoresistive	~11.45	0~1.5 kPa	[84]
Carbonized Silk Fabric		Piezoresistive	9.6<br>37.5	0%~250%<br>250%~500%	[85]

References

1. Meng, L. et al. Enhancing the performance of room temperature ZnO microwire gas sensor through a combined technology of surface etching and UV illumination. *Mater. Lett.* **212**, 296-298 (2018).
2. Fu, J. et al. Optical measurement of the converse piezoelectric d_{33} coefficients of bulk and microtubular zinc oxide crystals. *Appl. Phys. Lett.* **90**, 191 (2007).
3. Fan, H. et al. Template-assisted large-scale ordered arrays of ZnO pillars for optical and piezoelectric applications. *Small* **2**, 561-568 (2006).
4. Lee, Y. et al. Control of ZnO nanorod array alignment synthesized via seeded solution growth. *J. Cryst. Growth* **304,** 80-85 (2007).
5. Scrymgeour, D. & Hsu, J. Correlated piezoelectric and electrical properties in individual ZnO nanorods. *Nano Lett.* **8**, 2204-2209 (2008).
6. Minaryjolandan, M. et al. Individual GaN nanowires exhibit strong piezoelectricity in 3D. *Nano Lett.* **12**, 970 (2016).
7. Ke, T. et al. Sodium niobate nanowire and its piezoelectricity. *J. Phys. Chem. C* **112**, 8827-8831 (2008).
8. Wang, J. et al. Piezoresponse force microscopy on doubly clamped $KNbO_3$ nanowires. *Appl. Phys. Lett.* **93**, 223101 (2008).
9. Yang, R., Qin, Y., Dai, L. & Wang, Z. L. Power generation with laterally packaged piezoelectric fine wires. *Nat. Nanotechnol.* **4**, 34-39 (2009).
10. Zhou, J. et al. Flexible piezotronic strain sensor. *Nano Lett.* **8**, 3035-3040 (2008).
11. Wu, W., Wei, Y. & Wang, Z. L. Strain-gated piezotronic logic nanodevices. *Adv. Mater.* **22**, 4711-4715 (2010).
12. Hu, Y. et al. Temperature dependence of the piezotronic effect in ZnO nanowires. *Nano Lett.* **13**, 5026-5032 (2013).
13. Yu, R. et al. Temperature dependence of the piezophototronic effect in CdS nanowires. *Adv. Funct. Mater.* **25**, 5277-5284 (2015).
14. Yang, X. et al. Coupled ion-gel channel-width gating and piezotronic interface gating in ZnO nanowire devices. *Adv. Funct. Mater.* **29**, 1807837 (2019).
15. Sze, S. M. & Ng, K. K. *Physics of Semiconductor Devices*, 3rd ed. John Wiley & Sons (2006).
16. Tamm, I. Uber eine mogliche Art der Elektronenbindung an Kristalloberflachen *Physikalische Zeitschrift Der Sowjetunion* **1**, 733 (1933).

17. Shockley, W. & Pearson, G. Modulation of conductance of thin films of semiconductors by surface charges. *Phys. Rev.* **74**, 232 (1948).
18. Shockley, W. On the surface states associated with a periodic potential. *Phys. Rev.* **56**, 317 (1939).
19. Nicollian, E. & Brews, J. MOS Physics and Technology, John Wiley & Sons (1982).
20. Wu, W., Wen, X. & Wang, Z. L. Taxel-addressable matrix of vertical-nanowire piezotronic transistors for active and adaptive tactile imaging. *Science* **340**, 952-957 (2013).
21. Wu, W. & Wang, Z. L. Piezotronics and piezo-phototronics for adaptive electronics and optoelectronics. *Nat. Rev. Mater.* **7**, 1-17 (2016).
22. Pan, C., Zhai, J. & Wang, Z. L. Piezotronics and piezo-phototronics of third generation semiconductor nanowires. *Chem. Rev.* **119**, 9303-9359 (2019).
23. Zhang, Y., Liu, Y. & Wang, Z. L. Fundamental theory of piezotronics, *Adv. Mater.* **23**, 3004-3013 (2011).
24. Liu, S. et al. Ultrasensitive 2D ZnO piezotronic transistor array for high resolution tactile imaging. *Adv. Mater.* **29**, 1606346 (2017).
25. Liu, S. et al. Double-channel piezotronic transistors for highly sensitive pressure sensing. *ACS Nano* **12**, 1732-1738 (2018).
26. Henisch, H. K. Rectifying semiconductor contacts. Clarendon Press, Oxford (1957).
27. Schottky, W. Halbleitertheoerie der sperrschicht. *Naturwissenschaften* **26**, 843 (1938).
28. Bethe, H. A. Radiation Lab. *Report* **43**, 12 (1942).
29. Hwang, W. S., Park, H. C. & Hwang, W. Vibration control of a laminated plate with piezoelectric sensor/actuator: finite element formulation and modal analysis. *J. Intell. Mater. Syst. Struct.* **4**, 317-329 (1993).
30. Suiker, A. S. J. & Chang, C. S. Application of higher-order tensor theory for formulating enhanced continuum models. *Acta Mech.* **142**, 223-234 (2000).
31. Liu, Y. et al. Effect of hydrostatic pressure on the barrier height of Ni Schottky contacts on *n*-AlGaN. *Appl. Phys. Lett.* **88**, 022109 (2006).
32. Liu, Y. et al. Effects of hydrostatic and uniaxial stress on the Schottky barrier heights of Ga-polarity and N-polarity *n*-GaN. *Appl. Phys. Lett.* **84**, 2112-2114 (2004).

33. Liu, K., Sakurai, M. & Aono, M. Enhancing the humidity sensitivity of Ga_2O_3/SnO_2 core/shell microribbon by applying mechanical strain and its application as a flexible strain sensor. *Small* **8**, 3599-3604 (2012).
34. Tavassolian, M. Textile-based electromagnetic soft strain sensors for fast frequency movement and their application in wearable devices measuring multi-axial hip joint angles during running. *Adv. Intelligent Systems* 2020.
35. Bardeen, J. Surface states and rectification at a metal semi-conductor contact. *Phys. Rev.* 71, 717-727 (1947).
36. Goetzberger, A. Ideal MOS curves for silicon. *Bell System Technical Journal* **20**, 607 (1966).
37. Wang, Z. L. & Song, J. Piezoelectric nanogenerators based on zinc oxide nanowire arrays. *Science* **312**, 242-246 (2006).
38. Monaghan, S., Hurley, P. K., Cherkaoui, K., Negara, M. A. & Schenk, A. Determination of electron effective mass and electron affinity in HfO_2 using MOS and MOSFET structures. *Solid State Electron*. **53**, 438-444 (2009).
39. Liu, S., Wang, L., Feng, X., Liu, J., Qin, Y. & Wang, Z. L. Piezotronic tunneling junction gated by mechanical stimuli. *Adv. Mater.* **31**, 1905436 (2019).
40. Wang, P. et al. Asymmetric behavior in flexible piezoelectric strain sensors made of single ZnO nanowires. *J. Nanosci. Nanotechnol.* **14**, 6084-6088 (2014).
41. Sun, K. et al. Growth of ultralong ZnO microwire and its application in isolatable and flexible piezoelectric strain sensor. *Phys. Status Solidi A* **207**, 488-492 (2010).
42. Nakamura, K. First-principles simulation on wire diameter dependence of piezoresistivity in zinc oxide nanowires. *Jpn. J. Appl. Phys.* **54**, 06FJ11 (2015).
43. Sun, X., Gu, Y., Wang, X., Zhang, Z. & Zhang, Y. Strain-modulated transport properties of Cu/ZnO-nanobelt/Cu nanojunctions. *Phys. Status Solidi B* **252**, 1767-1772 (2015).
44. Zhang, G., Luo, X., Zheng, Y. & Wang, B. Giant piezoelectric resistance effect of nanoscale zinc oxide tunnel junctions: first principles simulations. *Phys. Chem. Chem. Phys.* **14**, 7051-7058 (2012).
45. Shao, R. W et al. Bandgap engineering and manipulating electronic and optical properties of ZnO nanowires by uniaxial strain. *Nanoscale* **6**, 4936-4941 (2014).
46. Yang, Y., Guo, W., Qi, J. & Zhang, Y. Flexible piezoresistive strain sensor based on single Sb-doped ZnO nanobelts. *Appl. Phys. Lett.* **97**, 223107 (2010).

47. Zhang, Z. et al. Highly efficient piezotronic strain sensors with symmetrical Schottky contacts on the monopolar surface of ZnO nanobelts. *Nanoscale* **7**, 1796-1801 (2015).
48. Yang, Y., Qi, J. J., Gu, Y. S., Wang, X. Q. & Zhang, Y. Piezotronic strain sensor based on single bridged ZnO wires. *Phys. Status Solidi RRL* **3**, 269-271 (2009).
49. Xue, F. et al. Influence of external electric field on piezotronic effect in ZnO nanowires. *Nano Res.* **8**, 2390-2399 (2015).
50. Zhao, Z. et al. Piezotronic effect in polarity-controlled GaN nanowires. *ACS Nano* **9**, 8578-8583 (2015).
51. Zhou, Y. S. et al. Nano-newton transverse force sensor using a vertical GaN nanowire based on the piezotronic effect. *Adv. Mater.* **25**, 883-888 (2013).
52. Zhou, Y. S. et al. Vertically aligned CdSe nanowire arrays for energy harvesting and piezotronic devices. *ACS Nano* **6**, 6478-6482 (2012).
53. Han, X., Du, W., Yu, R., Pan, C. & Wang, Z. L. Piezo-phototronic enhanced UV sensing based on a nanowire photodetector array. *Adv. Mater.* **27**, 7963-7969 (2015).
54. Zhang, W., Zhu, R., Nguyen, V. & Yang, R. Highly sensitive and flexible strain sensors based on vertical zinc oxide nanowire arrays. *Sens.* Actuator *A Phys.* **205**, 164-169 (2014).
55. Pan, C. et al. High-resolution electroluminescent imaging of pressure distribution using a piezoelectric nanowire LED array. *Nat. Photonics* **7**, 752-758 (2013).
56. Wang, L. et al. Ultrasensitive vertical piezotronic transistor based on ZnO twin nanoplatelet. *ACS Nano* **11**, 4859-4865 (2017).
57. Baraki, R., Novak, N., Frömling, T., Granzow, T. & Rödel, J. Bulk ZnO as piezotronic pressure sensor. *Appl. Phys. Lett.* **105**, 111604 (2014).
58. Wu, J. M. et al. Ultrahigh sensitive piezotronic strain sensors based on a $ZnSnO_3$ nanowire/microwire. *ACS Nano* **6**, 4369-4374 (2012).
59. Li, X., Wei, X., Xu, T., Pan, D., Zhao, J. & Chen, Q. Remarkable and crystal-structure-dependent piezoelectric and piezoresistive effects of InAs nanowires. *Adv. Mater.* **27**, 2852-2858 (2015).
60. Lee, T., Lee, W., Kim, S. W., Kim, J. J. & Kim, B. S. Flexible textile strain wireless sensor functionalized with hybrid carbon nanomaterials supported ZnO nanowires with controlled aspect ratio. *Adv. Funct. Mater.* **26**, 6206-6214 (2016).
61. Park, M., Park, Y. J., Chen, X., Park, Y. K., Kim, M. S. & Ahn, J. H. MoS_2-Based

tactile sensor for electronic skin applications. *Adv. Mater.* **28**, 2556-2562 (2016).

62. Ma, Y. et al. A highly flexible and sensitive piezoresistive sensor based on MXene with greatly changed interlayer distances. *Nat. Commum.* **8**, 1207 (2017).
63. Shao, R., Zheng, K., Zhang, Y., Li, Y., Zhang, Z. & Han, X. Piezoresistance behaviors of ultra-strained SiC nanowires. *Appl. Phys. Lett.* **101**, 233109 (2012).
64. Nguyen, T. et al. Giant piezoresistive effect by optoelectronic coupling in a heterojunction. *Nat. Commun.* **10**, 4139 (2019).
65. Zheng, K. et al. Observation of enhanced carrier transport properties of Si <100>-oriented whiskers under whiskers under uniaxial strains. *Appl. Phys. Lett.* **104**, 013111 (2014).
66. Lugstein, A., Steinmair, M., Steiger, A., Kosina, H. & Bertagnolli, E. Anomalous piezoresistance effect in ultrastrained silicon nanowires. *Nano Lett.* **10**, 3204-3208 (2010).
67. Huang, M., Pascal, T. A., Kim, H., Goddard, W. A. & Greer, J. R. Electronic-mechanical coupling in graphene from in situ nanoindentation experiments and multiscale atomistic simulations. *Nano Lett.* **11**, 1241-1246 (2011).
68. Greil, J., Lugstein, A., Zeiner, C., Strasser, G. & Bertagnolli, E. Tuning the electro-optical properties of germanium nanowires by tensile strain. *Nano Lett.* **12**, 6230-6234 (2012).
69. Takei, K. et al. Nanowire active-matrix circuitry for low-voltage macroscale artificial skin. *Nat. Mater.* **9**, 821-826 (2010).
70. Gong, S. et al. A wearable and highly sensitive pressure sensor with ultrathin gold nanowires. *Nat. Commun.* **5**, 3132 (2014).
71. Cao, J., Wang, Q. & Dai, H. Electromechanical properties of metallic, quasimetallic, and semiconducting carbon nanotubes under Stretching. *Phys. Rev. Lett.* **90**, 157601 (2003).
72. Yamada, T. et al. A stretchable carbon nanotube strain sensor for human-motion detection. *Nat. Nanotechnol.* **6**, 296-301 (2011).
73. Li, Y., Luo, S., Yang, M. C., Liang, R. & Zeng, C. Poisson ratio and piezoresistive sensing: a new route to high-performance 3D flexible and stretchable sensors of multimodal sensing capability. *Adv. Funct. Mater.* **26**, 2900-2908 (2016).
74. Wu, X., Han, Y., Zhang, X., Zhou, Z. & Lu, C. Large-area compliant, low-cost,

and versatile pressure-sensing platform based on microcrack-designed carbon black@polyurethane sponge for human-machine interfacing. *Adv. Funct. Mater.* **26**, 6246-6256 (2016).

75. Mattmann, C., Clemens, F. & Tröster, G. Sensor for measuring strain in textile. *Sensors* **8**, 3719-3732 (2008).
76. a) Kim, K. S. et al. Large-scale pattern growth of graphene films for stretchable transparent electrodes. *Nature* **457**, 706-710 (2009). b) Wang, Y. et al. Super-elastic graphene ripples for flexible strain sensors. *ACS Nano* **5**, 3645-3650 (2011).
77. Zhao, J. et al. Ultra-sensitive strain sensors based on piezoresistive nanographene films. *Appl. Phys. Lett.* **101**, 063112 (2012).
78. Hempel, M., Nezich, D., Kong, J. & Hofmann, M. A novel class of strain gauges based on layered percolative films of 2D materials. *Nano Lett.* **12**, 5714-5718 (2012).
79. Yan, C. et al. Highly stretchable piezoresistive graphene-nanocellulose nanopaper for strain sensors. *Adv. Mater.* **26**, 2022-2027 (2014).
80. Lee, Y. et al. Wafer-scale synthesis and transfer of graphene films. *Nano Lett.* **10**, 490-493 (2010).
81. Luo, S. & Liu, T. SWCNT/Graphite nanoplatelet hybrid thin films for self-temperature-compensated, highly sensitive, and extensible piezoresistive sensors. *Adv. Mater.* **25**, 5650-5657 (2013).
82. Roh, E., Lee, H. B., Kim, D. I. & Lee, N, E. A solution-processable, omnidirectionally stretchable, and high-pressure-sensitive piezoresistive device. *Adv. Mater.* **29**, 1703004 (2017).
83. Mannsfeld, S. C. B. et al. Highly sensitive flexible pressure sensors with microstructured rubber dielectric layers. *Nat. Mater.* **9**, 859-864 (2010).
84. Pang, C. et al. A flexible and highly sensitive strain-gauge sensor using reversible interlocking of nanofibres. *Nat. Mater.* **11**, 795-801 (2012).
85. Wang, C. et al. Carbonized silk fabric for ultrastretchable, highly sensitive, and wearable strain sensors. *Nat. Mater.* **28**, 6640-6648 (2016).
