# Supplementary material for: Highly sensitive strain sensors based on piezotronic tunneling junction
Source: Nat Commun. 2022 Feb 9;13:778. doi: 10.1038/s41467-022-28443-0 (PMC8828782; doi:10.1038/s41467-022-28443-0)
Supplement: Supplementary file 2 — Inventory of Supporting Information [file 41467_2022_28443_MOESM2_ESM.docx]

**Inventory of Supporting Information for**

**Highly Sensitive Strain Sensors Based on Piezotronic Tunneling Junction**

**Supplementary Notes 1-14:**

Supplementary Note 1 | Material Characterization

Supplementary Note 2 | Piezoelectricity in wurtzite ZnO

Supplementary Note 3 | Calculation of the Strain of the Sensors

Supplementary Note 4 | Asymmetry *I*-*V* Characteristics of the Tunneling Junctions without Strain

Supplementary Note 5 | Interface Traps/Defects in Tunneling Junctions

Supplementary Note 6 | Different Regulation Mechanisms of Piezoelectric Charges and Interface Traps/Defects on Electrical Transport under Strain

Supplementary Note 7 | Influence of the Ag/n-ZnO Contact Area of the Side Surface on the Performance of the Strain Sensor

Supplementary Note 8 | Electrical Transport of the Ag/n-ZnO Schottky-Junction-Based Strain Sensor (SSS)

Supplementary Note 9 | Calculation of the Change of Schottky Barrier Height and Its Linear Relationship with Strain

Supplementary Note 10 | Calculation of the Gauge Factor and the Current On-Off Ratio

Supplementary Note 11 | Theory of Piezotronic Effect on the Metal-Insulator-Semiconductor Junction

**Supplementary Figures 1-31:**

Supplementary Fig. 1 | Scanning electron microscopy (SEM) and transmission electron microscopy (TEM) images of ZnO microwire.

Supplementary Fig. 2 | The XRD spectrum of ZnO nanowires.

Supplementary Fig. 3 | Piezoelectricity in wurtzite ZnO.

Supplementary Fig. 4 | Piezoelectric nanogenerators under tensile strain and compressive strain to determine the polarity of ZnO microwire.

Supplementary Fig. 5 | Schematic of the measurement system.

Supplementary Fig. 6 | *I*-*V* characteristics of Ag/HfO_2_/n-ZnO devices.

Supplementary Fig. 7 | Influence of interface traps on *C*-*V* curves of Ag/HfO_2_/n-ZnO tunneling junctions.

Supplementary Fig. 8 | Strain-induced symmetric modulation of electrical transport by interface traps/defects.

Supplementary Fig. 9 | Strain-induced asymmetric modulation of electrical transport by piezotronic effect.

Supplementary Fig. 10 | Experiment results of *I*-*V* characteristics modulated by strain.

Supplementary Fig. 11 | Comparison of the strain-tuned *I*-*V* characteristics of two terminal devices with contact of Ag/HfO_2_/n-ZnO and Ag/HfO_2_/n-Si.

Supplementary Fig. 12 | The Ag/n-ZnO contacts at end/side surfaces and the equivalent circuits.

Supplementary Fig. 13 | Schematic of a metal-semiconductor-metal (MSM) piezotronic transistor and equivalent circuit of MSM piezotronic transistor.

Supplementary Fig. 14 | Ideal metal-semiconductor Schottky contacts with the presence of piezoelectric charges at an applied voltage *V=*0 (thermal equilibrium).

Supplementary Fig. 15 | Ideal metal-insulator-piezoelectric semiconductor contact with the presence of piezoelectric charges when applying positive voltage to metal.

Supplementary Fig. 16 | Barrier profiles.

Supplementary Fig. 17 | Potential distribution of the tunneling junction.

Supplementary Fig. 18 | Piezotronic modification of *C*-*V* characteristics of MIS tunneling junction.

Supplementary Fig. 19 | $\ln\left( {I_{\mathrm{strain}}}/{I_{\mathrm{free}}} \right)$ as a function of strain.

Supplementary Fig. 20 | Current response-recovery time curve for Ag/HfO_2_/n-ZnO PTSS.

Supplementary Fig. 21 | Current response-recovery time curve for Ag/n-ZnO SSS.

Supplementary Fig. 22 | The statistical distribution of the response time for Ag/HfO_2_/n-ZnO PTSS.

Supplementary Fig. 23 | The statistical distribution of the recovery time for Ag/HfO_2_/n-ZnO PTSS.

Supplementary Fig. 24 | The statistical distribution of the response time for Ag/n-ZnO SSS.

Supplementary Fig. 25 | The statistical distribution of the recovery time for Ag/HfO_2_/n-ZnO PTSS.

Supplementary Fig. 26 | The statistical distribution of the on-state and off-state current for Ag/HfO_2_/n-ZnO PTSS.

Supplementary Fig. 27 | The statistical distribution of the on-state and off-state current for Ag/n-ZnO SSS.

Supplementary Fig. 28 | *C*-*V* characteristics of MIS tunneling junctions with various insulator thicknesses.

Supplementary Fig. 29 | Measured *C*-*V* characteristics of Ag/HfO_2_/n-ZnO tunneling junctions for various insulator thicknesses.

Supplementary Fig. 30 | The on/off ratio of Ag/HfO_2_/n-ZnO tunneling junctions with various insulator thicknesses.

Supplementary Fig. 31 | The statistical distribution of the response time of this work (including Ag/HfO_2_/n-ZnO PTSS and Ag/n-ZnO SSS) and some other sensors.

**Supplementary Tables 1-2:**

Supplementary Table 1 | Response Time for Some Strain Sensing Works.

Supplementary Table 2 | Comparison of Strain Sensing Works.

**References 1-85.**
